# Electrocardiographic abnormalities in Chagas disease in the general population: A systematic review and meta-analysis

**DOI:** 10.1371/journal.pntd.0006567

**Published:** 2018-06-13

**Authors:** Lyda Z. Rojas, Marija Glisic, Laura Pletsch-Borba, Luis E. Echeverría, Wichor M. Bramer, Arjola Bano, Najada Stringa, Asija Zaciragic, Bledar Kraja, Eralda Asllanaj, Rajiv Chowdhury, Carlos A. Morillo, Oscar L. Rueda-Ochoa, Oscar H. Franco, Taulant Muka

**Affiliations:** 1 Department of Paediatrics, Obstetrics & Gynaecology and Preventative Medicine, Universidad Autónoma de Barcelona, Barcelona, Spain; 2 Department of Epidemiology, Erasmus MC, University Medical Center Rotterdam, Rotterdam, the Netherlands; 3 Grupo de Estudios Epidemiológicos y Salud Pública-FCV, Fundación Cardiovascular de Colombia, Floridablanca, Colombia; 4 Heart Failure and Heart Transplant Clinic, Fundación Cardiovascular de Colombia, Floridablanca, Colombia; 5 Medical Library, Erasmus MC, University Medical Center Rotterdam, Rotterdam, the Netherlands; 6 Department of Public Health & Primary Care, Cardiovascular Epidemiology Unit, University of Cambridge, Cambridge, United Kingdom; 7 Department of Cardiac Sciences, Cumming School of Medicine, University of Calgary, Calgary, Alberta, Canada; 8 Department of Medicine, Cardiology Division, McMaster University, PHRI-HHSC, Hamilton, Ontario, Canada; 9 Electrocardiography Research Group, Universidad Industrial de Santander, Bucaramanga, Colombia; 10 Institute of Social and Preventive Medicine (ISPM), University of Bern, Bern, Switzerland; Sacro Cuore Hospital, ITALY

## Abstract

**Background:**

Chagas disease (CD) is a major public health concern in Latin America and a potentially serious emerging threat in non-endemic countries. Although the association between CD and cardiac abnormalities is widely reported, study design diversity, sample size and quality challenge the information, calling for its update and synthesis, which would be very useful and relevant for physicians in non-endemic countries where health care implications of CD are real and neglected. We performed to systematically review and meta-analyze population-based studies that compared prevalence of overall and specific ECG abnormalities between CD and non-CD participants in the general population.

**Methods:**

Six databases (EMBASE, Ovid Medline, Web of Science, Cochrane Central, Google Scholar and Lilacs) were searched systematically. Observational studies were included. Odds ratios (OR) were computed using random-effects model.

**Results:**

Forty-nine studies were selected, including 34,023(12,276 CD and 21,747 non-CD). Prevalence of overall ECG abnormalities was higher in participants with CD (40.1%; 95%CIs=39.2-41.0) compared to non-CD (24.1%; 95%CIs=23.5-24.7) (OR=2.78; 95%CIs=2.37-3.26). Among specific ECG abnormalities, prevalence of complete right bundle branch block (RBBB) (OR=4.60; 95%CIs=2.97-7.11), left anterior fascicular block (LAFB) (OR=1.60; 95%CIs=1.21-2.13), combination of complete RBBB/LAFB (OR=3.34; 95%CIs=1.76-6.35), first-degree atrioventricular block (A-V B) (OR=1.71; 95%CIs=1.25-2.33), atrial fibrillation (AF) or flutter (OR=2.11; 95%CIs=1.40-3.19) and ventricular extrasystoles (VE) (OR=1.62; 95%CIs=1.14-2.30) was higher in CD compared to non-CD participants.

**Conclusions:**

This systematic review and meta-analysis provides an update and synthesis in this field. This research of observational studies indicates a significant excess in prevalence of ECG abnormalities (40.1%) related to *T*. *cruzi* infection in the general population from Chagas endemic regions, being the most common ventricular (RBBB and LAFB), and A-V B (first-degree) node conduction abnormalities as well as arrhythmias (AF or flutter and VE). Also, prevalence of ECG alterations in children was similar to that in adults and suggests earlier onset of cardiac disease.

## Author summary

Chagas disease (CD) is a major public health concern in Latin America and a potentially serious emerging threat in non-endemic countries. Although the association between CD and cardiac abnormalities is widely reported, study design diversity, sample size and quality challenge the information, calling for its update and synthesis, which would be very useful and relevant for physicians in non-endemic countries where health care implications of CD are real and neglected. We systematically reviewed and meta-analyzed population-based studies that compared prevalence of ECG abnormalities between Chagas disease (CD) and non-CD participants. Forty-nine studies were selected, including 34,023 unique participants. Our meta-analysis of observational studies indicates CD presented almost a threefold increase prevalence of ECG abnormalities than non-CD participants in the general population from Chagas endemic regions, being the most common ventricular (RBBB and LAFB), and A-V B (first-degree) node conduction abnormalities as well as arrhythmias (AF or flutter and VE).

## Introduction

Chagas disease (CD), is caused by the protozoan *Trypanosoma cruzi*[1]. It affects individuals from more than 21 countries, being a major public health concern in Latin Americas and a potentially serious emerging threat to a number of non-endemic countries[2]. In Latin American countries, there are currently 8-10 million people having CD, with an additional 300,000 individuals in the United States and 45,000-67,000 in Europe[3–6].

Infection with *T*. *cruzi* has two phases: acute and chronic, separated by an indeterminate period, in which the patient is relatively asymptomatic[7, 8]. Chronically infected patients ultimately develop cardiomyopathy, which is the most important and severe manifestation of CD, and is characterized by left ventricular systolic dysfunction, wall motion abnormalities, brady and tachyarrhythmia, heart failure and sudden cardiac death[9–18]. CD alterations are classified in four stages A, B, C, and D. Stage A corresponds to asymptomatic patients with normal ECG, whereas presence of electrocardiographic abnormalities implies progression towards stage B and deterioration of systolic function is observed in stages C/D, associated with heart failure symptoms. Of note, sudden cardiac death may occur at any moment, including early phases[10, 16].

The association between CD and cardiac abnormalities (stages B or more) is widely reported, however, the information is challenged by the diverse design, sample size and quality of the studies[19–24]. This information is largely based on outdated individual studies, and reports vary substantially among the population based studies making the scientific interpretation challenging[8, 20, 25–28]. Furthermore, it is known that the prevalence of different types of ECG abnormalities such as intraventricular conduction abnormalities, atrioventricular block, and arrhythmias, is higher in subjects with CD as compared to non-CD subjects, calling for its update and synthesis, which would be very useful and relevant for physicians in non-endemic countries where health care implications of CD are real and neglected[29]. Given the spread of CD, a need exists for an adequate, comprehensive assessment of CD in association with ECG abnormalities[6, 29, 30].

We conducted a systematic review and meta-analysis to (i) determine the overall prevalence of ECG abnormalities in seropositive and seronegative CD individuals in the general population; (ii) quantify the prevalence of subtypes of ECG abnormalities in this population; and (iii) characterize these estimates by age, sex and region of origin, and (iv) evaluate the discriminatory capacity of ECG abnormalities, both general and specific to classify patients as either CD or non-CD.

## Methods

### Data sources and search strategy

This review was conducted in accordance with MOOSE guideline[31] and, we followed the protocol strictly without deviation from it. An extensive literature search of articles published up to March 2017 was conducted with the assistance of a medical librarian in six electronic databases (EMBASE, Ovid Medline, Web of Science, Cochrane Central, Google Scholar and Lilacs) without any language restriction. Search combined terms related to CD (Chagas Disease, *Trypanosoma cruzi*), with terms related to seroepidemiology (Seroepidemiologic Studies, Seroprevalence, Seroepidemiology) **(S1 Appendix).** Reference lists of selected studies and reviews identified on the topic were searched to identify additional publications.

### Study selection and eligibility criteria

Studies were eligible if they: (i) selected cases and controls from the general population (surveys and blood donors); (ii) were cross-sectional, case-control and cohort studies; (iii) reported CD status based on any techniques for the detection of antibodies; and (iv) reported the prevalence of ECG abnormalities in CD participants and non-CD participants, based on electrocardiographic diagnosis. To avoid overestimation of the effect estimates, we did not include studies using cases and controls from a clinical setting. Two independent reviewers screened the titles and abstracts of all initially identified studies according to the selection criteria. Full texts were retrieved from studies that fulfilled all selection criteria. Any disagreements were resolved through consensus or consultation with a third independent reviewer.

### Data extraction

The data collection form included questions on qualitative aspects of the studies (such as author, date of publication, country, design, period, setting, area and sample size), participant characteristics (such as age, sex and serological test of CD) and information on the reported exposure/outcome (such as measure definition of ECG abnormalities, number of patients seropositive/seronegative CD, number of patients with general and specific ECG abnormalities and confounders adjustment). For cohort studies, only the information from the baseline assessment was extracted.

### Assessing the risk of bias

Study quality was assessed by two independent reviewers based on the nine-star Newcastle-Ottawa Scale (NOS) using three predefined domains, namely: selection of participants (population representativeness), comparability (adjustment for confounders) and ascertainment of outcomes of interests[32]. The NOS assigns a maximum of four or five points for selection, one or two points for comparability and three points for outcome, depending on study design (cross-sectional or cohort). We used the NOS scale adapted for cross-sectional studies and the NOS for case-control studies while for cohort studies we used the NOS for a cross-sectional design **(S2 Appendix).** Studies that received a score of nine to seven stars were judged to be at low risk of bias; studies that scored five or six stars were considered at medium risk, and those that scored four or less were considered at high risk of bias.

### Statistical analysis

Narrative synthesis and construction of descriptive summary tables were performed for the studies included. For this meta-analysis, we used odds ratios (ORs) and 95% confidence intervals (CIs), to assess the association between presence of CD, and overall and specific ECG abnormalities. Also, we calculated the pooled prevalence (P) of ECG abnormalities for CD and non-CD participants. The inverse variance weighted method was used to combine summary measures using random-effects models to minimize the effect of between-study heterogeneity[33]. Heterogeneity was assessed using the Cochrane χ^2^ statistic and the *I*^*2*^ statistic and was categorized as low (*I*^*2*^ ≤25%), moderate (*I*^*2*^ >25% and <75%), or high (*I*^*2*^ ≥75%)[34]. Sensitivity analyses were performed to assess the influence of study-level characteristics including publication year, geographical location (according to the number of study in each country and by the prevalence of *Trypanosoma cruzi* genotype), design, area, number and age of participants, sex, definition of ECG abnormalities, test for CD diagnoses, level of adjustment and risk of bias, which were pre-specified as characteristics for assessment of heterogeneity and were evaluated using stratified analysis and random-effects meta-regression[35]. Publication bias was evaluated through funnel plots and Egger’s regression symmetry tests[36]. All tests were 2-tailed; *p-value* ≤0.05 was considered statistically significant. Stata release 15 (StataCorp) was used for all analyses. Additionally, we evaluated the discriminatory capacity of ECG abnormalities, both general and specific: sensitivity, specificity, positive predictive value (PPV), negative predictive value (NPV) to classify patients as either CD or non-CD.

## Results

### Study identification and selection

**Fig 1:**
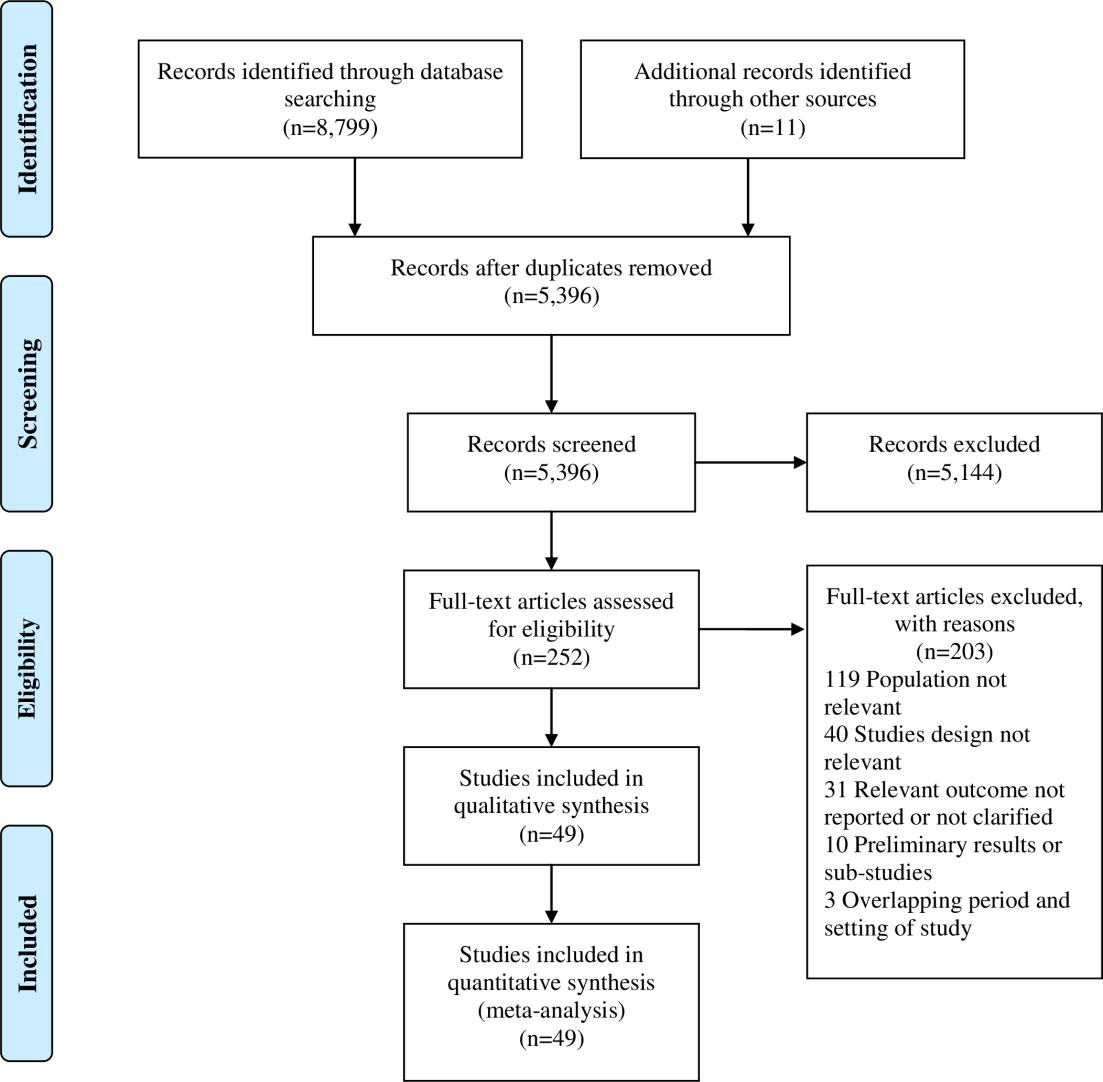
PRISMA flow diagram of selection process of eligible studies. From a total of 5,396 eligible studies, and 49 were included in meta-analysis.

The database searches identified 5,396 citations. After screening the titles and abstracts, 252 articles were selected for detailed evaluation of full texts. Of these, 49 articles met our inclusion criteria and were included in the review **(Fig 1, S3 Appendix and S1–S3 Tables).**

### General characteristics of included studies

General characteristics of included studies are shows in **S1–S3 Tables** summarize the key characteristics of the included studies. Of 49 included studies, 43 were cross-sectional and 6 cohort population-based studies and were published between 1964 and 2015. Studies were based on data from 10 Latin America countries. The majority of studies was conducted in Brazil (38.7%), collected data between 2001 and 2010 (34.6%), and included participants from rural areas (44.9%). In aggregate, 34,023 participants (12,276 CD and 21,747 non-CD) were included in this review. The age range of the participants was 0-97 years and 54.4% of participants were women (reported by 38 articles). The majority of studies (81.6%) used two or more different diagnostic tests for *T*. *cruzi* infection.

### Overall and specific ECG abnormalities

**Fig 2:**
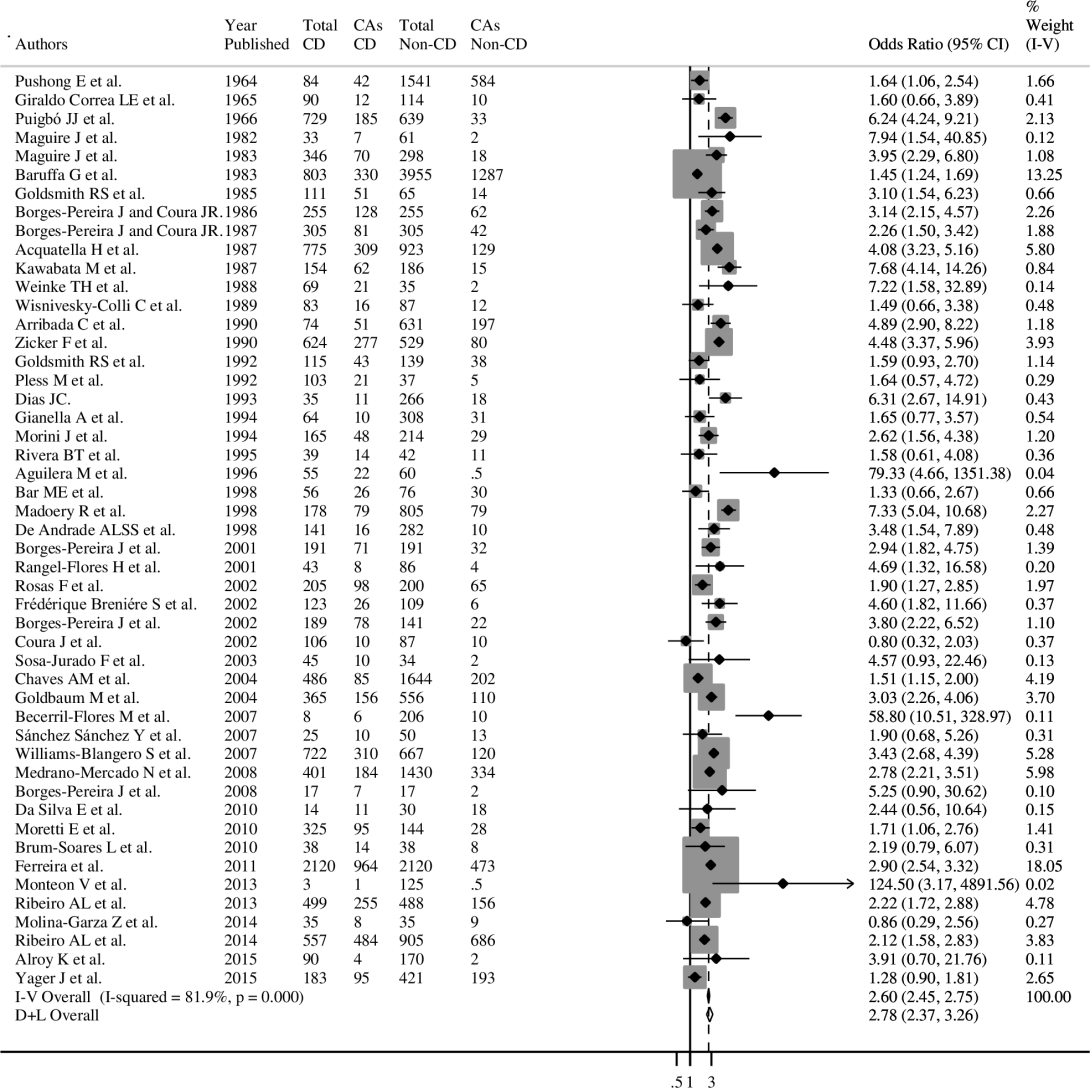
Meta-analyses of overall ECG abnormalities in Chagas and Non-Chagas disease. The **box** are proportional to the weight of each study in the analysis, and the **lines** represent their 95% confidence intervals (CIs). The **open diamond** represents the pooled odds ratio, and its width represents it’s 95% CI. **Abbreviations: CD=**Chagas disease: **CAs=**ECG abnormalities.

In the meta-analysis of 34,023 participants and 12,276 CD participants, overall ECG abnormalities were significantly more prevalent in participants with CD (Prevalence (P)=40.1%; 95%CIs=39.2-41.0) compared to those without CD (P=24.1%; 95%CIs=23.5-24.7) (OR=2.78; 95%CIs=2.37-3.26) **(Fig 2 and Table 1).**

**Table 1:** Meta-analysis of overall ECG abnormalities in CD and Non-CD by characteristics of studies.

Characteristics	Number of studies	Pooled ECG Abnormalities					Heterogeneity		p-value Cochran’s Q	p-value Meta-regression*
		Total CD	CAs in CD	Total Non-CD	CAs in Non-CD	OR (95% CI)	I^2^ (%)	p-value		
**Overall studies**	49	12,276	4,922	21,747	5,243	2.78 (2.37-3.26)	81.9	0.000	0.000	----
**Publication year**										
1964-1989	13	3,837	1,314	8,464	2,210	3.08 (2.12-4.48)	89.3	0.000	0.000	0.480
1990-2000	12	1,649	618	3,389	528	3.10 (2.11-4.57)	78.4	0.000		
2001-2010	17	3,303	1,179	5,630	986	2.64 (2.08-3.36)	68.6	0.000		
2011-2015	7	3,487	1,811	4,264	1,519	2.07 (1.48-2.90)	79.7	0.000		
**Location**										
Brazil	19	7,360	3,280	11,191	3,156	2.82 (2.30-3.46)	81.8	0.000	0.000	0.065
Mexico	7	360	127	690	77	4.12 (1.71-9.91)	76.6	0.000		
Bolivia	6	943	357	2,340	571	2.28 (1.42-3.66)	73.6	0.002		
Argentina	6	891	306	2,867	762	2.24 (1.22-4.12)	88.0	0.000		
Colombia	3	781	195	1,958	277	1.63 (1.30-2.03)	0.0	0.656		
Venezuela	2	1,504	494	1,562	162	4.90 (3.24-7.41)	70.4	0.066		
Chile	2	129	73	691	197	13.7 (0.98-191.4)	72.2	0.058		
Perú	2	115	14	220	15	2.29 (0.95-5.50)	0.0	0.478		
Ecuador	1	154	62	186	15	7.68 (4.14-14.26)	---	---		
Nicaragua	1	39	14	42	11	1.58 (0.61-4.08)	---	---		
**Primarily *T*. *cruzi* by country**										
T. *cruzi* I (Colombia, Mexico, Nicaragua and Venezuela)	13	2,684	830	4,252	527	2.87 (1.88-4.39)	84.6	0.000	0.373	0.549
T. *cruzi* II (Brazil)	19	7,360	3,280	11,191	3,156	2.82 (2.30-3.46)	81.8	0.000		
T. *cruzi* V (Argentina and Bolivia)	12	1,834	663	5,207	1,333	2.29 (1.60-3.30)	82.4	0.000		
**Design**										
Cross-sectional	43	10,475	3,997	18,445	4,146	2.91(2.44-3.47)	82.5	0.000	0.000	0.182
Cohort	6	1,801	925	3,302	1,098	2.01 (1.59-2.54)	47.9	0.087		
**Area**										
Rural	22	5,890	2,160	9,793	2,282	2.92 (2.24-3.80)	80.7	0.000	0.035	0.672
Urban	11	3,382	1,335	6,555	1,119	3.15 (2.40-4.14)	84.1	0.000		
**Number of participants**^†^										
≤100	8	246	81	307	65	2.12 (1.38-3.25)	6.7	0.378	0.410	0.701
101-1000	31	4,729	1,671	7,087	1,250	2.92 (2.34-3.64)	76.0	0.000		
≥1001	10	7,301	3,170	14,353	3,928	2.73 (2.06-3.63)	93.1	0.000		
**Age of participants**										
All ages	28	6,964	2,620	12,243	3,073	2.66 (2.13-3.32)	81.0	0.000	0.433	0.704
≥ 10 years	16	3,990	1,769	6,940	1,706	2.78 (2.08-3.71)	85.9	0.000		
Only children	3	597	222	1,772	344	3.88 (1.69-8.88)	63.9	0.062		
**Sex**										
Women	6	1,104	356	2,863	813	2.51 (1.66-3.81)	72.1	0.003	0.793	0.390
Men	6	893	336	2,213	603	3.77 (1.89-7.50)	84.9	0.000		
**Definition of CAs**										
Specific definitions	40	10,355	4,137	17,130	4,015	2.95 (2.44-3.57)	84.2	0.000	0.394	0.202
No-specified/no clear	9	1,921	785	4,617	1,229	2.44 (1.94-3.08)	54.3	0.025		
**Test for the diagnoses CD**										
One test for CD	9	4,630	1,754	9,402	2,496	2.77 (1.90-4.04)	91.4	0.000	0.006	0.970
More one test for CD	40	7,646	3,168	12,345	2,748	2.78 (2.33-3.32)	76.4	0.000		
**Confounders adjustment**^‡^										
Yes	18	4,550	1,873	5,008	1,021	2.53 (2.14-2.99)	40.2	0.040	0.419	0.370
No	31	7,726	3,049	16,739	4,222	2.97 (2.37-3.73)	87.3	0.000		
**Risk of bias**										
High	11	1,910	514	1,932	179	3.31 (2.14-5.10)	74.4	0.000	0.006	0.400
Medium	28	6,412	2,816	14,486	4,074	2.81 (2.24-3.52)	85.3	0.000		
Low	10	3,954	1,592	5,329	990	2.34 (1.79-3.04)	71.6	0.000		

*p-value for heterogeneity was evaluated using random-effects meta-regression

^†^Total positive and negative for Chagas disease

^‡^Adjusted by confounders as age, sex and others in design

**Abbreviations: CD=**Chagas disease; **CAs=**ECG abnormalities; **OR=**Odds ratio.

**Fig 3:**
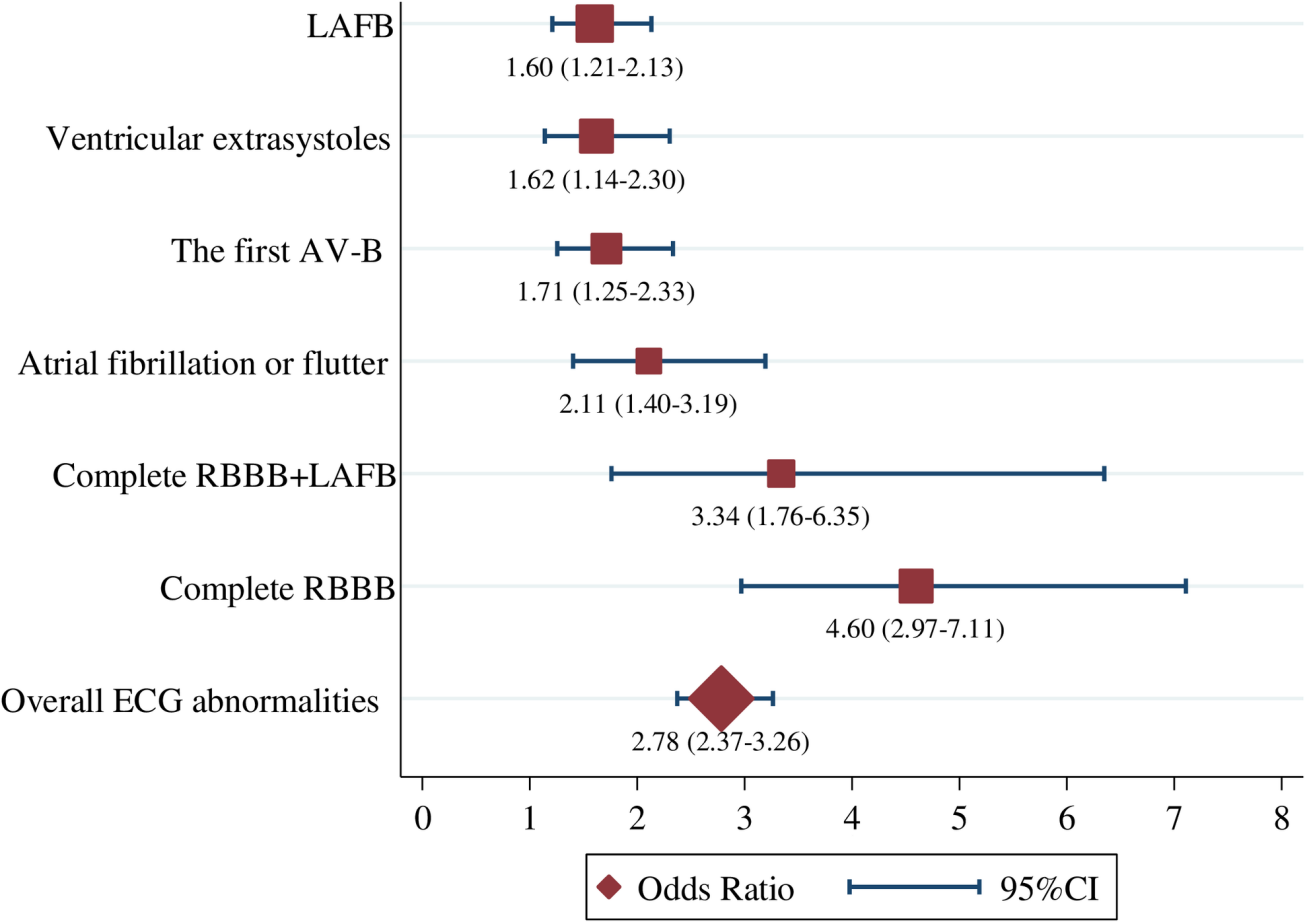
Prevalence of overall and specified ECG abnormalities in CD to compared non-CD participants. Forresplot determined the association between overall and specified ECG abnormalities with CD to compared Non-CD participants. **Abbreviations: CD=**Chagas disease; **RBBB=**Right bundle branch block; **LAFB=**Left anterior fascicular block; **AV-B=**Atrioventricular block.

Up to 30 studies (range 3 to 30), based on 3,451 ± 1,350 participants, examined prevalence of ventricular conduction defects in CD and non-CD participants (**Table 2**). In the pooled analysis, prevalence of complete right bundle branch block (RBBB) (OR=4.60; 95%CIs=2.97-7.11), left anterior fascicular block (LAFB) (OR=1.60; 95%CIs=1.21-2.13) and, the combination of complete RBBB and LAFB (OR=3.34, 95%CIs=1.76-6.35) was higher in CD participants compared to non-CD participants **(Fig 3)**, while no difference in prevalence of incomplete RBBB (OR=0.85; 95%CIs=0.63-1.16), incomplete left bundle branch block (LBBB) (OR=0.63; 95%CIs=0.39-1.01), complete LBBB (OR=0.99; 95%CIs=0.62-1.58) and left posterior fascicular block (LPFB) (OR=0.96, 95%CIs=0.35-2.62) was found between CD and non-CD individuals **(Table 2 and S1–S7 Figs).** Prevalence of AV-B in CD and non-CD participants was examined by 21 studies (range 5 to 21), including 4,438 ± 1,847 participants. Pooled prevalence first degree AV-B (OR=1.71; 95%CIs=1.25-2.33) was higher in CD participants compared to non-CD **(Fig 3)**, while no difference in prevalence of second-degree AV-B (OR=1.15; 95%CIs=0.33-4.06) and third-degree AV-B (OR=2.10; 95% CIs=0.60-7.34) was found between CD and non-CD participants **(Table 2 and S8–S10 Figs).** Twenty-five studies (range 11 to 25 studies), based on 4,848 ± 1,591 participants, compared prevalence of arrhythmias between CD and non-CD participants. Pooled prevalence of AF or flutter (OR=2.11, 95%CIs=1.40-3.19) and ventricular extrasystoles (VE) (OR=1.62; 95%CIs=1.14-2.30) was higher in CD participants **(Table 2, Fig 3 and S11–S14 Figs).** In a pooled analysis of 10 studies, comprising 5,575 individuals, no statistically significant difference in prevalence of other ECG abnormalities (Low voltage QRS, OR=0.84; 95%CIs=0.47-1.50) was found between CD and non-CD **(Table 2 and S15 Fig).**

**Table 2:** Meta-analysis of specified ECG abnormalities in CD and Non-CD.

Specified ECG abnormalities	Number of studies	Pooled specified ECG abnormalities					Heterogeneity		p-value Cochran’s Q
		Total CD	Events CD	Total Non-CD	Events Non-CD	OR (95% CI)	I^2^ (%)	p-value	
**Ventricular conduction defects**									
Incomplete RBBB	19	2,383	269	3,242	394	0.85 (0.63-1.16)	33.8	0.076	0.316
Complete RBBB	24	2,636	710	3,360	169	4.60 (2.97-7.11)	64.5	0.000	0.000
Incomplete LBBB	3	691	27	827	56	0.63 (0.39-1.01)	0.0	0.929	0.055
Complete LBBB	8	1,579	37	2,325	38	0.99 (0.62 -1.58)	19.1	0.278	0.958
LAFB	30	3,919	734	4,040	419	1.60 (1.21-2.13)	54.1	0.000	0.000
LPFB	7	1,317	9	1,002	12	0.96 (0.35-2.62)	6.9	0.375	0.933
Complete RBBB+LAFB	15	1,891	167	1,608	34	3.34 (1.76-6.35)	35.9	0.082	0.000
**Atrioventricular block**									
The first AV-B	21	3,112	161	3,457	77	1.71 (1.25-2.33)	10.9	0.317	0.001
Second degree AV-B	7	1,177	7	2,117	1	1.15 (0.33-4.06)	0.0	0.665	0.822
Third degree AV-B	5	1,275	9	2,176	2	2.10 (0.60-7.34)	0.0	0.923	0.246
**Arrhythmias**									
Atrial fibrillation	11	2,075	44	2,871	22	1.55 (0.86-2.78)	0.0	0.862	0.141
Atrial fibrillation or flutter	13	2,654	78	3,750	40	2.11 (1.40-3.19)	0.0	0.789	0.000
Ventricular extrasystoles	25	3,142	588	3,749	366	1.62 (1.14-2.30)	59.5	0.000	0.000
Supraventricular extrasystoles	12	2,004	73	2,525	91	1.14 (0.82-1.59)	2.8	0.417	0.428
**Other**									
Low voltage QRS	10	2,426	45	3,149	79	0.84 (0.47-1.50)	28.6	0.181	0.421

**Abbreviations: ECG=**Electrocardiogram; **CD=**Chagas disease; **OR=**Odds ratio; **RBBB=**Right bundle branch block; LBBB=Left bundle branch block; **LAFB=**Left anterior fascicular block; **LPFB=**Left posterior fascicular block; **AV-B=**Atrioventricular block.

### Study quality and assessment of bias

Regarding of study quality and assessment bias, among the 49 included studies, 10 studies were judged to be at low risk of bias, 28 were at medium risk, and 11 studies were evaluated to be at high risk of bias **(Table 1, S2 Appendix, S3 Table and S16 Fig).**

### Sensitivity analysis

In the sensitivity analysis, of 16 meta-analyses, while five showed no heterogeneity (*I*^*2*^=0, p>0.05 for the Cochrane χ2 statistic), four showed low (*I*^*2*^=<25%), six moderate and one high between-study heterogeneity, four of which with an *I*^*2*^ estimate exceeding 50% (overall ECG abnormalities, complete RBBB, LAFB and VE) (**Fig 2, Tables 1 and 2 and S4–S6 Tables**). No study characteristics could explain the heterogeneity observed in overall ECG abnormalities (p-value of meta-regression ≥0.05) **(Table 1 and S17–S27 Figs)**. Additionally, **S28 Fig** shows meta-analyses estimates of overall ECG abnormalities excluding single studies one by one, with no evidence of change in the pooled. Similarly, the stratified analysis by sex and age, although showed higher magnitudes of the association in children and men, the effects were not significantly different between sex and age strata: higher OR was observed in children (OR=3.88; 95%CIs=1.69-8.88; I2=63.9%) compared with all ages or ≥10 years categories; however, when a small-sample study was omitted, the OR of children was similar to that of the other categories (OR=2.83; 95%CIs=2.26-3.53; I2=0.0%) without being statistically significant in the meta-regression analysis (p-value of meta-regression=0.704), and comparison of overall ECG abnormalities by sex, could be quantified only in a limited number of studies (12.2%) **(Table 1, S25, S26 and S29 Figs)**. For the studies on the association between CD, complete RBBB, LAFB and VE, the identified heterogeneity was largely explained by geographic location, area, number of participants, number of tests used for diagnoses of CD, level of adjustment and risk of bias **(S4–S6 Tables)**.

Considering a P value of 0.10, rather than the conventional level of 0.05, is sometimes used to determine statistical significance for heterogeneity, we investigated further the sources of heterogeneity across our meta-analyses using a p-value lower than 0.10 as statistical significant. When setting the p-value threshold to 0.10, the findings of meta-regression were similar as when setting the p-value threshold of 0.05, except for the characteristics “location of the study” which on p-value of 0.10, showed to contribute to heterogeneity of the meta-analysis for overall ECG abnormalities.

### Publication bias

Under visual examination, funnel plots for studies assessing the general and specific prevalence of ECG abnormalities were symmetrical, providing evidence for no publication bias. This was further supported by the results of Egger’s test, showing no evidence of publication bias, either graphically from the funnel plot, or quantitatively (p≥0.05 for Egger’s test asymmetry) **(S7 Table and S30 Fig).**

### Discriminatory capacity of the ECG for diagnosis of CD

Finally, Sensitivity, specificity, PPV and NPV of general ECG abnormalities were 40.09%, 75.89%, 48.42% and 69.18%, respectively. PPV of specific ECG abnormalities for diagnosis of CD varied, ranging between 61.64% and 83.08%, with two ECG abnormalities showing values over 80%: complete RBBB (80.77%; 95%CIs=78.17-83.13) and the combination of complete RBBB and LAFB (83.08%; 95%CIs= 77.36-87.59) **(S8 Table).**

## Discussion

We systematically reviewed the current evidence for the prevalence of overall and specific ECG abnormalities in CD individuals in the general population. Overall, we found that subjects with CD, compared to non-CD participants, have almost a threefold higher prevalence of ECG alterations. Namely, a higher prevalence of complete RBBB, LAFB, first degree AV-B, VE, combined AF and flutter and, the combination of RBBB and LAFB, whereas no difference in other ECG abnormalities were observed between CD and non-CD participants. Also, prevalence of ECG alterations in children was similar to that in adults and suggests earlier onset of cardiac disease.

The current study complements and extends findings from a previous systematic review[37] that reported higher prevalence of RBBB and LAFB but no difference in AF in CD participants compared to non-CD participants. However, our review included 34,023 vs. 17,238 participants more with an additional 19 papers (49 vs. 30 studies), overlapping only on 10 studies (20.4%) with the previous review. Further, contrary to the current effort the previous work included clinical and community cases, with controls being mainly from the general population, whereas our study included only population-based studies comparing ECG alterations compatible with CD in seropositive and seronegative subjects recruited from the non-clinical setting. Studies using cases from a clinical setting and controls from the general population, including the mentioned systematic review, tend to overestimate the prevalence of ECG alterations. Furthermore, Cardoso et al.[37] found no data available to calculate prevalence of 1st and 2nd degree AV-B in CD and non-CD individuals, whereas our study quantified the prevalence of these ECG alterations, documenting a higher prevalence of first degree AV-B block. Our findings further extend previous reports by providing the prevalence of overall and specific ECG alterations, reporting a higher prevalence of combined AF/flutter and VE among CD compared to non-CD participants. In contrast, no differences in incomplete and complete LBBB, LPFB, second and third degree AV-B, supraventricular extrasystoles and low voltage QRS were observed.

Finally, even though childhood heart involvement is an uncommon event and cardiac alterations are considered to take decades to ensue in adults with *T*. *cruzi* infection[38–40], our data indicates that CD is associated with a higher prevalence of ECG alterations both in children and adults. Also, our findings on high frequency of ECG alterations in children (5-13 years) suggest earlier manifestations of anomalies progression when *T*. *cruzi* infection is acquired at a younger age [38, 41, 42]. A previous clinicopathologic study based on medical records and autopsies, carried out in 19 children and adolescents (6-17 years) determined a prevalence of ventricular premature contractions 58%, LAFB 45%, first degree A-V block 28%, complete RBBB 11%, AF 11%; the authors concluded that, in this adolescent population, CD shows relevant peculiarities, like rapid evolution toward decease (as short as 128 days), diagnosis difficulty related to occurrence of mitral regurgitation (61% wrong initial diagnosis) and low RBBB frequency, suggesting that CD at this age range can be of a peculiar type, making clarification of the pathogenic mechanism of chronic CD in teens useful to pinpoint the pathogenic mechanism of the disease[43].

Although the etiology of cardiac alterations in CD-patients remains unclear, a number of biologically plausible mechanisms have been suggested. Cardiac alterations are characterized by development of a diffuse cellular infiltrate, microcirculation alterations that lead to fibrosis[44]. Evidence suggests that low grade parasite persistence triggers a parasite-specific immune response to *T*. *cruzi* in the heart, and that at least some of the cells comprising the infiltrate are parasite-specific[44]. Simões-Barbosa et al. reported that *T*. *cruzi* infection may affect gene expression in human host cells via LINE-1 retrotransposon, which may play an important role in the pathogenesis of CD cardiac involvement[45, 46]. *T*. *cruzi* infection also impairs parasympathetic innervations predisposing to sympathetic over-activation which may lead to further cardiac damage and arrhythmogenesis[47, 48]. *T*. *cruzi* persistence may directly initiate vascular endothelial cell damage or lead to microvasculature damage via its effects on the inflammatory infiltrate as well as on several bioactive lipids such as thromboxane A and prostaglandin F2α, potent vasoconstrictors[44]. In summary, multiple mechanisms may be responsible for the development of cardiac alterations in individuals infected with *T*. c*ruzi* that include host and specific parasite characteristics that need further investigation.

There were several strengths and limitations of the present study. Our analysis included more than 34,023 seropositive and seronegative CD participants, and investigated a wide range of ECG abnormalities. We also included only studies based on subjects recruited through community surveys and blood bank screening (population-based), in order to avoid overestimation of effect measures that may arise by inclusion of clinical populations. As with all systematic reviews we are prone to publication bias. However, there was little evidence of publication bias, and therefore, the likelihood that studies with negative findings are not published is low. We performed an extensive search strategy in several databases in order to identify all relevant studies in the topic. Nevertheless, since majority of studies were from Latin America, and we did not use terms in Portuguese and Spanish language in our search, we do not exclude the possibility to have missed some relevant studies. However, the search strategy we used included the term “Chagas” that has no translation, so we believe that it did not introduce selection bias or if it did, it would be minor. Additionally, the low and moderate quality of the individual published studies included in this meta-analysis was limited. Variation in study regarding inclusion and exclusion criteria and the diverse populations included in different studies, contributed to the heterogeneity of findings noted in several of the meta-analyses performed. Other sources of heterogeneity are likely to include different diagnostic methods for CD, and variation in the criteria applied for the definition of ECG abnormalities. However, we did not observe any heterogeneity in 5 out of 16 meta-analyses, as well as in the meta-analysis based only on children.

The cardiac manifestations in *T*. *cruzi* infected subjects, documented by the presence of specific ECG changes such as complete RBBB and LAFB in its initial stages. As disease advances, AV-B, VE and atrial fibrillation/flutter may ensue[49, 50]. These findings may be relevant for diagnostic and epidemiologic purposes as once cardiac alterations are established the prognosis of these subjects might deteriorate rapidly. These findings may have important implications for non-endemic countries, where the prevalence of CD in Latin-American migrants is 4.2% (95% CIs=2.2-6.7%)[6], in which less than 1% of the expected prevalence of CD-cardiomyopathy has been reported, suggesting under diagnosis and suboptimal physician awareness[51]. Specific ECG alterations such as RBBB associated with LAFB, AV-B, atrial fibrillation/flutter and VE, should alert the clinician encountering patients with heart failure or cardiomyopathy of unknown etiology originally form Chagas endemic regions, to consider obtaining serologic tests to rule out *T*. *cruzi* infection[30]. Finally, the increased prevalence of ECG abnormalities in children underscores the need for early diagnosis and trypanocidal treatment should be considered.

CD is recognized by World Health Organization as a neglected tropical disease that historically, has not received sufficient public health interest, or research and governmental resources worldwide. CD costs in Latin America are estimated up to 662,000 disability-adjusted life-years of productivity as of 2008, and the total economic toll attributed to the disease each year is estimated at over $7 billion USD, with more than 10% of this cost being incurred in the United States and Canada. Eminent scientists have labeled Chagas Disease as ‘‘The New HIV/AIDS of the Americas’’, and 20–30% of people infected with Chagas disease will develop potentially fatal cardiomyopathy in their lifetime. Yet, there is no vaccine or highly effective medical treatment available for the approximately 10 million people currently infected with *T*. *cruzi* [52].

In terms of public health, our findings provide an update and synthesis of the burden of cardiac disease in Chagas patients, and complement previous findings on excesses mortality in this individuals. Cucunuba ZM et al. [40] showed that excess mortality exists in CD, both in symptomatic and asymptomatic populations. In addition, our results provide important data that could be useful for futures studies to better estimate the health and economic burden of CD in Latinamerica. Understanding morbidity and mortality in Chagas patients at population level as shown in this study, provides a better understanding of the disease burden and impact, and highlights the need for better detection, diagnosis, treatment and management of affected populations. Improved morbid-mortality Chagas disease estimates at population level will serve to highlight the real need for better detection, diagnostics, treatment and management of affected populations[14].

### Conclusion

This systematic review and meta-analysis provides an update and synthesis in this field. This research of observational studies indicates a significant excess in prevalence of ECG abnormalities (40.1%) related to *T*. *cruzi* infection in the general population from Chagas endemic regions, being the most common ventricular (RBBB and LAFB), and A-V B (first-degree) node conduction abnormalities as well as arrhythmias (AF or flutter and VE). Also, prevalence of ECG alterations in children was similar to that in adults and suggests earlier onset of cardiac disease.

## Supporting information

S1 Checklist PRISMA checklist. (DOC)

S1 Appendix Literature search for studies that investigated the prevalence of ECG abnormalities in Chagas disease. (DOCX)

S2 Appendix Assessments of study quality of the studies included in systematic review and meta-analysis. (DOCX)

S3 Appendix Reference list of included studies in this meta-analysis. (DOCX)

S1 Table General characteristics of the studies included in systematic review and meta-analysis of prevalence of ECG abnormalities in Chagas disease. (DOCX)

S2 Table Definition of ECG abnormalities in each study included in systematic review and meta-analysis. (DOCX)

S3 Table Summary of studies and participants characteristics (49 included studies). (DOCX)

S4 Table Sensitivity analysis: Complete right bundle branch block (CRBBB). (DOCX)

S5 Table Sensitivity analysis: Left anterior fascicular block (LAFB). (DOCX)

S6 Table Sensitivity analysis: Ventricular extrasystoles (VE). (DOCX)

S7 Table Assessment of small study effects by Egger’s test. (DOCX)

S8 Table Discriminatory capacity of the ECG for the diagnosis of Chagas diseases. (DOCX)

S1 Fig Pooled odds ratios for the complete right bundle branch block (CRBBB) in Chagas disease. (TIF)

S2 Fig Pooled odds ratios for the left anterior fascicular block (LAFB) in Chagas disease. (TIF)

S3 Fig Pooled odds ratios for the combination complete right bundle branch block (CRBBB) and left anterior fascicular block (LAFB) in Chagas disease. (TIF)

S4 Fig Pooled odds ratios for the incomplete right bundle branch block (IRBBB) in Chagas disease. (TIF)

S5 Fig Pooled odds ratios for the incomplete left bundle branch block (ILBBB) in Chagas disease. (TIF)

S6 Fig Pooled odds ratios for the complete left bundle branch block (CLBBB) in Chagas disease. (TIF)

S7 Fig Pooled odds ratios for the left posterior fascicular block (LPFB) in Chagas disease. (TIF)

S8 Fig Pooled odds ratios for the first degree left atrioventricular block (A-V B) in Chagas disease. (TIF)

S9 Fig Pooled odds ratios for the second degree left atrioventricular block (A-V B) in Chagas disease. (TIF)

S10 Fig Pooled odds ratios for the third degree left atrioventricular block (A-V B) in Chagas disease. (TIF)

S11 Fig Pooled odds ratios for atrial fibrillation (AF) in Chagas disease. (TIF)

S12 Fig Pooled odds ratios for supraventricular extrasystoles (SE) in Chagas disease. (TIF)

S13 Fig Pooled odds ratios for atrial fibrillation (AF) or flutter in Chagas disease. (TIF)

S14 Fig Pooled odds ratios for ventricular extrasystoles (VE) in Chagas disease. (TIF)

S15 Fig Pooled odds ratios for low voltage QRS (LV-QRS) in Chagas disease. (TIF)

S16 Fig Pooled odds ratios for the prevalence of ECG abnormalities in Chagas disease by assessments of study quality. (TIF)

S17 Fig Pooled odds ratios for the prevalence of ECG abnormalities in Chagas disease by period of publication. (TIF)

S18 Fig Pooled odds ratios for the prevalence of ECG abnormalities in Chagas disease by location. (TIF)

S19 Fig Pooled odds ratios for the prevalence of ECG abnormalities in Chagas disease by design. (TIF)

S20 Fig Pooled odds ratios for the prevalence of ECG abnormalities in Chagas disease by area. (TIF)

S21 Fig Pooled odds ratios for the prevalence of ECG abnormalities in Chagas disease by area (Rural vs Urban). (TIF)

S22 Fig Pooled odds ratios for the prevalence of ECG abnormalities in Chagas disease by test for the diagnoses. (TIF)

S23 Fig Pooled odds ratios for the prevalence of ECG abnormalities in Chagas disease by confounders adjustment. (TIF)

S24 Fig Pooled odds ratios for the prevalence of ECG abnormalities Chagas disease by number of participants. (TIF)

S25 Fig Pooled odds ratios for the prevalence of ECG abnormalities Chagas disease by age of participants. (TIF)

S26 Fig Pooled odds ratios for the prevalence of ECG abnormalities Chagas disease by sex of participants. (TIF)

S27 Fig Pooled odds ratios for the prevalence of ECG abnormalities in Chagas disease by definition of ECG abnormalities. (TIF)

S28 Fig Meta-analysis estimates (Overall ECG abnormalities), given named study is omitted. (TIF)

S29 Fig Pooled odds ratios for the prevalence of ECG abnormalities Chagas disease by age of participants (exclude Aguilera M et al. 1996). (TIF)

S30 Fig Assessment of small study effects by funnel plots. (DOCX)

S1 Diagram PRISMA flow diagram. (DOC)
