# Supplementary material for: Electrocardiographic abnormalities in Chagas disease in the general population: A systematic review and meta-analysis
Source: PLoS Negl Trop Dis. 2018 Jun 13;12(6):e0006567. doi: 10.1371/journal.pntd.0006567 (PMC5999094; doi:10.1371/journal.pntd.0006567)
Supplement: S1 Appendix — (DOCX) [file pntd.0006567.s002.docx]

Relevant studies, published before March 15^th^ 2017 (last date searched) were identified through electronic searches not limited to the English language using EMBASE, Ovid Medline, Web of Science, Cochrane Central, Google Scholar and Lilacs. Electronic searches were supplemented by scanning reference lists of articles identified for all relevant studies (including review articles), by hand searching of relevant journals (Revista de la Sociedad Brasileira de Medicina Tropical and Memórias Do Instituto Oswaldo Cruz). The computer-based searches combined search terms related to population (Chagas disease and *Trypanosoma cruzi*), and to study design without language restriction:

1. **EMBASE strategy to identify relevant studies:**

('Chagas disease'/exp OR 'Trypanosoma cruzi'/de OR trypanosomiasis/de OR 'African trypanosomiasis'/de OR (Chagas* OR (Trypanosoma NEAR/3 infect*) OR 't cruzi' OR trypanosomiasis OR 'Sleeping Sickness'):ab,ti) AND ('prevalence'/de OR 'seroprevalence'/de OR 'seroepidemiology'/de OR 'incidence'/de OR 'epidemiological data'/de OR 'disease surveillance'/de OR (prevalen* OR seroprevalen* OR Seroepidemiolog* OR incidence* OR seropositiv* OR (epidemiolog* NEAR/3 (data* OR monitor* OR surveil*)) OR ((serolog* OR population*) NEAR/3 screen*)):ab,ti) NOT ([animals]/lim NOT [humans]/lim)

1. **Ovid Medline strategy to identify relevant studies:**

(exp "Chagas Disease"/ OR "Trypanosomiasis, African"/ OR "Trypanosoma cruzi"/ OR Trypanosomiasis/ OR (Chagas* OR (Trypanosoma ADJ3 infect*) OR "t cruzi" OR trypanosomiasis OR "Sleeping Sickness").ab,ti.) AND ("Prevalence"/ OR "Seroepidemiologic Studies"/ OR "incidence"/ OR "Epidemiological Monitoring"/ OR "Epidemiologic Methods"/ OR (prevalen* OR seroprevalen* OR incidence* OR seropositiv* OR (epidemiologic* ADJ3 data*) OR ((serolog* OR population*) ADJ3 screen*)).ab,ti.) NOT (exp animals/ NOT humans/)

1. **Web of Science strategy to identify relevant studies:**

TS=(((Chagas* OR (Trypanosoma NEAR/2 infect*) OR "t cruzi" OR trypanosomiasis OR "Sleeping Sickness")) AND ((prevalen* OR seroprevalen* OR Seroepidemiolog* OR incidence* OR seropositiv* OR (epidemiolog* NEAR/2 (data* OR monitor* OR surveil*)) OR ((serolog* OR population*) NEAR/2 screen*))) NOT ((animal* OR dog OR dogs OR canine OR cat OR cats OR feline OR mouse OR mice OR rat OR rats OR murine OR buffalo OR cow OR cows OR cattle OR livestock OR bovine OR sheep OR ovine OR primate* OR nonhuman OR "non human" OR bird OR birds OR fish OR horse OR camel* OR turtle* OR ranch OR game OR monkey* OR bear OR bears OR pig OR pigs OR swine OR porcine) NOT (human* OR patient*))) AND DT=(article)

1. **Cochrane Central strategy to identify relevant studies:**

((Chagas* OR (Trypanosoma NEAR/3 infect*) OR 't cruzi' OR trypanosomiasis OR 'Sleeping Sickness'):ab,ti) AND ((prevalen* OR seroprevalen* OR Seroepidemiolog* OR incidence* OR seropositiv* OR (epidemiolog* NEAR/3 (data* OR monitor* OR surveil*)) OR ((serolog* OR population*) NEAR/3 screen*)):ab,ti)

1. **Google Scholar strategy to identify relevant studies:**

Chagas|"Trypanosoma infection"|trypanosomiasis|"Sleeping Sickness" prevalence|seroprevalence|Seroepidemiology|incidence|seropositivity|"epidemiologic|epidemiological data|monitoring|surveillance"|"serological|population screening"

**(vi) Lilacs 1884 strategy to identify relevant studies:**

(Chagas OR "Trypanosoma infection" OR trypanosomiasis OR "Sleeping Sickness") AND (prevalence OR seroprevalence OR Seroepidemiology OR incidence OR seropositivity OR epidemiologic OR epidemiological OR serological OR "population screening" )
