# Supplementary material for: Electrocardiographic abnormalities in Chagas disease in the general population: A systematic review and meta-analysis
Source: PLoS Negl Trop Dis. 2018 Jun 13;12(6):e0006567. doi: 10.1371/journal.pntd.0006567 (PMC5999094; doi:10.1371/journal.pntd.0006567)
Supplement: S2 Appendix — (DOCX) [file pntd.0006567.s003.docx]

| **Authors/Publication Year** | **Design** | **Stars** | | | |
| --- | --- | --- | --- | --- | --- |
|  |  | **Selection** | **Comparability** | **Outcome/Exposure** | **Total** |
| Pushong E et al. 1964 | Cross-sectional | ******** |  | ****** | 6 |
| Giraldo Correa LE et al. 1965 | Cross-sectional | ******* |  | ***** | 4 |
| Puigbó JJ et al. 1966 | Cross-sectional | ******* |  | ***** | 4 |
| Maguire J et al. 1982 | Prospective cohort* | ******** |  | ***** | 5 |
| Maguire J et al. 1983 | Cross-sectional | ******* |  | ***** | 4 |
| Baruffa G et al. 1983 | Cross-sectional | ******* |  | ****** | 5 |
| Goldsmith RS et al. 1985 | Prospective cohort* | ******** |  | ******* | 7 |
| Borges-Pereira and Coura JR. 1986 | Cross-sectional | ******** | ***** | ***** | 6 |
| Borges-Pereira J and Coura JR. 1987 | Cross-sectional | ******** | ***** | ***** | 6 |
| Acquatella H et al. 1987 | Cross-sectional | ******* |  | ****** | 5 |
| Kawabata M et al. 1987 | Cross-sectional | ***** |  | ***** | 2 |
| Weinke TH et al. 1988 | Cross-sectional | ****** |  | ****** | 4 |
| Wisnivesky-Colli C et al. 1989 | Cross-sectional | ******** |  | ***** | 5 |
| Arribada C et al. 1990 | Cross-sectional | ******* |  | ****** | 5 |
| Zicker F et al. 1990 | Cross-sectional | ******* | ***** | ****** | 6 |
| Pless M et al. 1992 | Cross-sectional | ******** |  | ******* | 7 |
| Goldsmith RS et al. 1992 | Prospective cohort* | ****** |  | ******* | 5 |
| Dias JC. 1993 | Cross-sectional | ****** |  | ***** | 3 |
| Morini J et al. 1994 | Cross-sectional | ****** |  | ******* | 5 |
| Gianella A et al. 1994 | Cross-sectional | ******* |  | ****** | 5 |
| Rivera BT et al. 1995 | Cross-sectional | ******** | ***** | ******* | 8 |
| Aguilera M et al. 1996 | Cross-sectional | ****** | ****** | ******* | 7 |
| Bar ME et al. 1998 | Cross-sectional | ******* |  | ***** | 4 |
| De Andrade ALSS et al. 1998 | Cross-sectional | ******** | ****** | ******* | 9 |
| Madoery et al. 1998 | Cross-sectional | ******* |  | ****** | 5 |
| Rangel-Flores H et al. 2001 | Cross-sectional | ****** | ***** | ***** | 4 |
| Borges-Pereira J et al. 2001 | Cross-sectional | ******* | ***** | ****** | 6 |
| Borges-Pereira J et al. 2002 | Cross-sectional | ******* |  | ****** | 5 |
| Frédérique Breniére S et al. 2002 | Cross-sectional | ******** |  | ****** | 6 |
| Coura J et al. 2002 | Cross-sectional | ******* | ***** | ***** | 5 |
| Rosas F et al. 2002 | Cross-sectional | ******* | ***** | ***** | 5 |
| Sosa-Jurado F et al. 2003 | Cross-sectional | ****** | ***** | ****** | 5 |
| Goldbaum M et al. 2004 | Cross-sectional | ******** | ***** | ******* | 8 |
| Chaves AM et al. 2004 | Prospective cohort* | ******* | ****** | ******* | 8 |
| Becerril-Flores M et al. 2007 | Cross-sectional | ******** |  | ****** | 6 |
| Sánchez Sánchez Y et al. 2007 | Cross-sectional |  | ***** | ******* | 4 |
| Williams-Blangero S et al. 2007 | Cross-sectional | ******** |  | ****** | 6 |
| Medrano-Mercado N et al. 2008 | Cross-sectional | ******** |  | ***** | 5 |
| Borges-Pereira J et al. 2008 | Cross-sectional | ******** | ***** | ***** | 6 |
| Da Silva E et al. 2010 | Cross-sectional | ******** | ***** | ***** | 6 |
| Brum-Soares L et al. 2010 | Cross-sectional | ****** |  | ****** | 4 |
| Moretti E et al. 2010 | Cross-sectional | ****** |  | ***** | 3 |
| Ferreira et al. 2011 | Cross-sectional | ******* | ***** | ******* | 7 |
| Monteon V et al. 2013 | Cross-sectional | ******** |  | ***** | 5 |
| Ribeiro AL et al. 2013 | Prospective cohort^†^ | ******* | ****** | ****** | 7 |
| Ribeiro AL et al. 2014 | Prospective cohort* | ******** |  | ****** | 6 |
| Molina-Garza Z et al. 2014 | Cross-sectional | ******** | ***** | ******* | 8 |
| Yager J et al. 2015 | Cross-sectional | ******* |  | ******* | 6 |
| Alroy K et al. 2015 | Cross-sectional | ******* | ***** | ****** | 6 |

*****ECG abnormalities were reported as base characteristic in a cross-sectional study and it assessed as cross-sectional study; ^†^It was assessed as case-control study.
