# Supplementary material for: Electrocardiographic abnormalities in Chagas disease in the general population: A systematic review and meta-analysis
Source: PLoS Negl Trop Dis. 2018 Jun 13;12(6):e0006567. doi: 10.1371/journal.pntd.0006567 (PMC5999094; doi:10.1371/journal.pntd.0006567)
Supplement: S3 Appendix — (DOCX) [file pntd.0006567.s004.docx]

[1] Pushong E, Lopez F, Pellegrini C, Gracia J. Epidemiological investigation of the prevalence of Chagas Disease. Bol Oficina Sanit Panam. 1964;56:357-67.

[2] Giraldo Correa LE, Bernal Tirado J, Corredor Arjona A. Comparative study of the electrocardiographic changes in 2 regions with different incidences of chagasic infections. Rev Fac Med Univ Nac Colomb. 1965;33:115-25.

[3] Puigbo JJ, Rhode JR, Barrios HG, Suarez JA, Yepez CG. Clinical and epidemiological study of chronic heart involvement in Chagas' disease. Bull World Health Organ. 1966;34:655-69.

[4] Maguire JH, Mott KE, Hoff R. A three-year follow-up study of infection with Trypanosoma cruzi and electrocardiographic abnormalities in a rural community in Northeast Brazil. AM J TROP MED HYG. 1982;31:42-7.

[5] Maguire JH, Mott KE, Lehman JS. Relationship of electrocardiographic abnormalities and seropositivity to Trypanosoma cruzi within a rural community in Northeast Brazil. AM HEART J. 1983;105:287-94.

[6] Baruffa Giovanni, Alcantara Filho Alcino, JO AN. Correlação sorológica eletrocardiográfica para a doença de Chagas em populações rurais não selecionadas do Rio Grande do Sul. Rev Soc Bras Med Trop. 1983;16:130-8.

[7] Goldsmith RS, Zarate RJ, Zarate LG, Kagan I, Jacobson LB. Clinical and epidemiologic studies of Chagas' disease in rural communities in Oaxaca State, Mexico, and a seven-year follow-up: I. Cerro del Aire. Bull Pan Am Health Organ. 1985;19:120-38.

[8] Pereira J, Coura J. Morbidade da doença de Chagas: estudo seccional em uma área endêmica, Virgem da Lapa, Minas Gerais. Rev Soc Bras Med Trop. 1986;19:139-48.

[9] Pereira J, Coura J. Morbidade da doença de Chagas em populações urbanas do sertão da Paraíba. Rev Soc Bras Med Trop. 1987;20:101-7.

[10] Acquatella H, Catalioti F, Gomez-Mancebo JR. Long-term control of Chagas disease in Venezuela: Effects on serologic findings, electrocardiographic abnormalities, and clinical outcome. CIRCULATION. 1987;76:556-62.

[11] Kawabata M, Uchiyama T, Mimori T, Hashiguchi Y, de Coronel VV. Association of electrocardiographic abnormalities with seropositivity to Trypanosoma cruzi in Ecuador. Trans R Soc Trop Med Hyg. 1987;81:7-10.

[12] Weinke T, Ueberreiter K, Alexander M. Cardiac morbidity due to Chagas' disease in a rural community in Bolivia. EPIDEMIOL INFECT. 1988;101:655-60.

[13] Wisnivesky-Colli C, Ruiz AM, Gurtler RE, Solarz ND, Lazzari J, Ledesma O, et al. Dynamics of transmission of Trypanosoma cruzi in a rural area of Argentina. IV. Serologic, parasitologic and electrocardiographic study of the human population. Medicina (B Aires). 1989;49:341-50.

[14] Arribada A, Apt W, Aguilera X, Solari A, Arribada A, Sandoval J. Chagas cardiopathy in the first region of Chile. Clinical, epidemiologic, and parasitologic study. Rev Med Chil. 1990;118:846-54.

[15] Zicker F, Caetano de Almeida Netto J, Zicker EMS, Oliveira RM, Smith PG. Trypanosoma cruzi infection and electrocardiographic findings among active manual workers. A population-based study in central Brazil. INT J EPIDEMIOL. 1990;19:182-6.

[16] Pless M, Juranek D, Kozarsky P, Steurer F, Tapia G, Bermudez H. The epidemiology of Chagas' disease in a hyperendemic area of Cochabamba, Bolivia: A clinical study including electrocardiography, seroreactivity to Trypanosoma cruzi, xenodiagnosis, and domiciliary triatomine distribution. AM J TROP MED HYG. 1992;47:539-46.

[17] Goldsmith RS, Zarate RJ, Zarate LG, Morales G, Kagan I, Drickey R, et al. Clinical and epidemiologic studies of Chagas' disease in rural communities of Oaxaca, Mexico, and an eight-year followup: II. Chila. Bull Pan Am Health Organ. 1992;26:47-59.

[18] Dias JC. The clinical, social and occupational aspects of Chagas disease in an endemic area under the control of the state of Minas Gerais, Brazil. Rev Soc Bras Med Trop. 1993;26:93-9.

[19] Morini JC, Berra H, Dávila HO, Pividori JF, Bottasso OA. Electrocardiographic alteration among first degree relatives with serologic evidence of Trypanosoma cruzi infection. A sibship study. Mem Inst Oswaldo Cruz. 1994;89:371-5.

[20] Gianella A, von Poser B, Zamora P. Chagas' infection in university students of Santa Cruz de la Sierra, Bolivia. A serologic-electrocardiographic study. Rev Inst Med Trop Sao Paulo. 1994;36:515-8.

[21] Rivera T, Palma-Guzman R, Morales W. Seroepidemiological and clinical study of Chagas' disease in Nicaragua. Rev Inst Med Trop Sao Paulo. 1995;37:207-13.

[22] Aguilera Martín X, Arribada Contreras A, Apt Baruch W, Zulantay Alfaro I, Rodríguez T J. Detección de cardiopatía en escolares chagásicos y no chagásicoa de Combarbalá, IV región, Chile. Rev Chil Pediatr. 1996;67:104-8.

[23] Bar ME, Pozzer DL, Alvarez BM, Vallejos JA, Storino RA. Clinical cross-sectional and epidemiologic study of Chagas disease in a rural area of the Argentinian northeast. Rev Soc Bras Med Trop. 1998;31:199-206.

[24] De Andrade ALSS, Zicker F, Rassi A, Rassi AG, Oliveira RM, Silva SA, et al. Early electrocardiographic abnormalities in Trypanosoma cruzi- seropositive children. Am J Trop Med Hyg. 1998;59:530-4.

[25] Madoery RJ, Lúquez H, De Loredo L, Reyes ME, De Roiter H, Lombardelli S, et al. Enfermedad de Chagas: indicador serológico y electrocardiografía convencional en un área urbana de región endémica (Deán Funes, Córdoba, Argentina). Rev Argent Cardiol. 1998;66:413-22.

[26] Rangel-Flores H, Sánchez B, Mendoza-Duarte J, Barnabé C, Brenière FS, Ramos C, et al. Serologic and parasitologic demonstration of Trypanosoma cruzi infections in an urban area of central Mexico: Correlation with electrocardiographic alterations. Am J Trop Med Hyg. 2001;65:887-95.

[27] Borges-Pereira J, Zauza PL, Galhardo MC, Nogueira J, Pereira GR, Cunha RV. Chagas' disease in a urban population of the health district of Rio Verde, Mato Grosso do Sul State, Brazil. Rev Soc Bras Med Trop. 2001;34:459-66.

[28] Borges-Pereira J, De Castro JAF, Furtado Campos JH, De Souza Nogueira J, Lago Zauza P, Marques P, et al. Study of the infection and morbidity of Chagas' disease in municipality of Jaão Costa - National Park Serra da Capivara, Piauí, Brazil. Rev Soc Bras Med Trop. 2002;35:315-22.

[29] Brenière SF, Bosseno MF, Noireau F, Yacsik N, Liegeard P, Aznar C, et al. Integrate study of a Bolivian population infected by Trypanosoma cruzi, the agent of Chagas disease. Mem Inst Oswaldo Cruz. 2002;97:289-95.

[30] Coura JR, Junqueira AC, Boia MN, Fernandes O, Bonfante C, Campos JE, et al. Chagas disease in the Brazilian Amazon: IV. a new cross-sectional study. Rev Inst Med Trop Sao Paulo. 2002;44:159-65.

[31] Rosas F, Guhl F, Velasco V, Jumbo L, Jaramillo C, Rodriguez D, et al. Morbilidad de la enfermedad de Chagas en fase crónica en Colombia. Detección de paceintes chagásicos con cardiopatía en un área endémica del departamento de Boyacá. Rev Col Cardiol. 2002;9:349-59.

[32] Sosa-Jurado F, Mazariego-Aranda M, Hernández-Becerril N, Garza- Murillo V, Cárdenas M, Reyes PA, et al. Electrocardiographic findings in Mexican chagasic subjects living in high and low endemic regions of Trypanosoma cruzi infection. Mem Inst Oswaldo Cruz. 2003;98:605-10.

[33] Goldbaum M, Ajimura FY, Litvoc J, De Carvalho SA, Eluf-Neto J. American trypanosomiasis and electrocardiographic alterations among industrial workers in São Paulo, Brazil. Rev Inst Med Trop Sao Paulo. 2004;46:299-302.

[34] Chávez AM, Villar JC, Herrera M, Martínez LX, Cardona SR, Casadiego G. Electrocardiographic study in subjects with positive and negative serology for Tripanosoma Cruzi. Rev Col Cardiol. 2004;11:246-50.

[35] Becerril-Flores MA, Rangel-Flores E, Imbert-Palafox JL, Gómez-Gómez JV, Figueroa-Gutiérrez AH. Human infection and risk of transmission of Chagas disease in Hidalgo State, Mexico. Am J Trop Med Hyg. 2007;76:318-23.

[36] Sánchez Sánchez Y, Córdova Benzaquen E, Vásquez Huerta L, Bocangel Bravo C, Velásquez Talavera R, Delgado Díaz F, et al. Estudio comparativo de alteraciones electrocardiográficas, frecuencia cardiaca y presión arterial entre seropositivos y seronegativos para Trypanosoma cruzi en el valle de Vítor, Arequipa-Perú. Acta méd peru. 2007;24:6-10.

[37] Williams-Blangero S, Magalhaes T, Rainwater E, Blangero J, Corrêa-Oliveira R, VandeBerg JL. Electrocardiographic characteristics in a population with high rates of seropositivity for Trypanosoma cruzi infection. Am J Trop Med Hyg. 2007;77:495-9.

[38] Medrano-Mercado N, Ugarte-Fernandez R, Butrón V, Uber-Busek S, Guerra H, Araújo-Jorge T, et al. Urban transmission of Chagas disease in Cochabamba, Bolivia. Mem Inst Oswaldo Cruz. 2008;103:423-30.

[39] Borges-Pereira J, Sarquis O, Zauza PL, Britto C, Lima MM. Epidemiology of Chagas disease in four rural localities in Jaguaruana, State of Ceará. Seroprevalence of infection, parasitemia and clinical characteristics. Rev Soc Bras Med Trop. 2008;41:345-51.

[40] Da Silva EM, Rocha MOC, Silva RC, Paixão GC, Buzzati H, Santos AN, et al. Clinic and epidemiological study on Chagas disease in the Serra Azul district of Mateus Leme, central-western region of the State of Minas Gerais, Brazil. Rev Soc Bras Med Trop. 2010;43:178-81.

[41] Brum-Soares LM, Xavier SS, Silvestre de Sousa A, Borges-Pereira J, Marcos Bemfica Barbosa Ferreira J, Costa IR, et al. Morbidity of Chagas disease among autochthonous patients from the Rio Negro microregion, State of Amazonas. Rev Soc Bras Med Trop. 2010;43:170-7.

[42] Moretti E, Castro I, Franceschi C, Basso B. Chagas disease: Serological and electrocardiographic studies in Wichi and Creole communities of Misión Nueva Pompeya, Chaco, Argentina. Mem Inst Oswaldo Cruz. 2010;105:621-6.

[43] Ferreira Gonçalves J, Prata A, Dias JCP, Macêdo V. O inquérito eletrocardiográfico. Rev Soc Bras Med Trop. 2011;44:40-6.

[44] Monteon V, Alducin C, Hernandez J, Ramos-Ligonio A, Lopez R. High frequency of human blood in Triatoma dimidiata captured inside dwellings in a rural community in the Yucatan Peninsula, Mexico, but low antibody seroprevalence and electrocardiographic findings compatible with Chagas disease in humans. Am J Trop Med Hyg. 2013;88:566-71.

[45] Ribeiro AL, Sabino EC, Marcolino MS, Salemi VMC, Ianni BM, Fernandes F, et al. Electrocardiographic Abnormalities in Trypanosoma cruzi Seropositive and Seronegative Former Blood Donors. PLoS Negl Trop Dis. 2013;7.

[46] Ribeiro AL, Marcolino MS, Prineas RJ, Lima-Costa MF. Electrocardiographic abnormalities in elderly Chagas disease patients: 10-year follow-up of the Bambui Cohort Study of Aging. J Am Heart Assoc. 2014;3:e000632.

[47] Molina-Garza ZJ, Rosales-Encina JL, Mercado-Hernández R, Molina-Garza DP, Gomez-Flores R, Galaviz-Silva L. Association of Trypanosoma cruzi infection with risk factors and electrocardiographic abnormalities in northeast Mexico. BMC Infect Dis. 2014;14.

[48] Yager JE, Lozano Beltran DF, Torrico F, Gilman RH, Bern C. Prevalence of Chagas Heart Disease in a Region Endemic for Trypanosoma Cruzi Evidence from a Central Bolivian Community. Glo Heart. 2015;10:145-50.

[49] Alroy KA, Huang C, Gilman RH, Quispe-Machaca VR, Marks MA, Ancca-Juarez J, et al. Prevalence and Transmission of Trypanosoma cruzi in People of Rural Communities of the High Jungle of Northern Peru. PLoS Negl Trop Dis. 2015;9.
