# Supplementary material for: Electrocardiographic abnormalities in Chagas disease in the general population: A systematic review and meta-analysis
Source: PLoS Negl Trop Dis. 2018 Jun 13;12(6):e0006567. doi: 10.1371/journal.pntd.0006567 (PMC5999094; doi:10.1371/journal.pntd.0006567)
Supplement: S1 Table — (DOCX) [file pntd.0006567.s005.docx]

| **Lead author, Publication date** | **Country** | **Study design*** | **Study period** | **Setting study** | **Area** | **Sample size** | **Age group (Years)** ^†^ | **Women (%)** | **Diagnosis of Chagas disease** | **Adjusted by confounder** |
| --- | --- | --- | --- | --- | --- | --- | --- | --- | --- | --- |
| Pushong E et al. 1964 [1] | Argentina | Cross-sectional | ---- | Population | Rural/ Periurban | 1625 | 6-89 | ---- | CFT | No |
| Giraldo Correa LE et al.1965 [2] | Colombia | Cross-sectional | November 1962 to August 1964 | Population | Rural | 204 | 1-61+ | 50.4 | Blood Smear/XD/ Serology | No |
| Puigbó JJ et al. 1966 [3] | Venezuela | Cross-sectional | 1961 to 1964 | Population | Rural | 1368 | 5-65+ | 52.0 | CFT | No |
| Maguire J et al. 1982 [4] | Brazil | Prospective cohort* | July to August 1976 | Population | Rural | 94 | <5-45+ | ---- | CFT/IFI/XD/ Culture for T. cruzi | No |
| Maguire J et al. 1983 [5] | Brazil | Cross-sectional | December 1973 to February 1974 | Population | Rural | 644 | 10-75+ | 55.1 | CFT | No |
| Baruffa G et al. 1983 [6] | Brazil | Cross-sectional | July 1970 to July 1975 | Population | Rural | 4758 | 5-70+ | 53.1 | CFT | No |
| Goldsmith RS et al. 1985 [7] | Mexico | Prospective cohort* | 1971 to 1980 | Population | ---- | 176 | 0-60+ | 50.8 | IHA/DA/ CFT/XD/ Culture for T. cruzi | No |
| Borges-Pereira and Coura JR. 1986 [8] | Brazil | Cross-sectional | January to April 1982 | Population | Urban | 510 | 6-60+ | 59.9 | CFT/IFI/ IHA/XD | Design: sex, age |
| Borges-Pereira J and Coura JR. 1987 [9] | Brazil | Cross-sectional | November 1984 to January 1985 | Population | Urban | 610 | 1-60+ | 64.2 | IFI | Design: age, sex, city |
| Acquatella H et al.1987 [10] | Venezuela | Cross-sectional | June 1981 to June 1984 | Blood Donors/ Clinical/ Population | Rural/ Urban | 1698 | 0-59+ | ---- | CFT/IF/IHA | No |
| Kawabata M et al. 1987 [11] | Ecuador | Cross-sectional | ---- | Population/ Clinical | ---- | 340 | 10-60+ | 60.9 | IHA | No |
| Weinke TH et al. 1988 [12] | Bolivia | Cross-sectional | ---- | Population | Rural | 104 | 15-53 | 100 | CFT/IFI | No |
| Wisnivesky-Colli C et al. 1989 [13] | Argentina | Cross-sectional | August 1980 to November 1982 | Population | Rural | 170 | 0-45+ | ---- | IHA/IFI/DA/CFT/ ELISA | No |
| Arribada C et al. 1990 [14] | Chile | Cross-sectional | ---- | Population | Rural | 705 | 0-90 | 55.7 | IHA/IFI/XD | No |
| Zicker F et al. 1990 [15] | Brazil | Cross-sectional | 1988 to 1989 | Population | Urban | 1153 | 16-73 | 22.8 | IF/ELISA/HA | Analysis: age, sex, institutions |
| Pless M et al. 1992 [16] | Bolivia | Cross-sectional | June to July 1988 | Population | Rural | 140 | 5-85 | ---- | IFI/CFT | No |
| Goldsmith RS et al. 1992 [17] | Mexico | Prospective cohort* | 1971 to 1980 | Population | ---- | 254 | 0-60+ | 50.4 | IHA/DA/ CFT/XD/ Culture for T. cruzi | No |
| Dias JC. 1993 [18] | Brazil | Cross-sectional | 1984 to 1986 | Population | Urban | 301 | 18-57 | 0 | IHA/IFI | No |
| Morini J et al. 1994 [19] | Argentina | Cross-sectional | ---- | Population | Rural | 379 | 33±19.0 | 56.2 | IFI/IHA | No |
| Gianella A et al. 1994 [20] | Bolivia | Cross-sectional | First semester of 1993 | Population | Rural/ Urban | 372 | 19.3±2.6 | 48.6 | IHA | No |
| Rivera BT et al. 1995 [21] | Nicaragua | Cross-sectional | 1989 to 1992 | Population | Rural | 81 | 0-50+ | ---- | IFI/IHA | Design: age, sex |
| Aguilera M et al. 1996 [22] | Chile | Cross-sectional | ---- | Population | ---- | 115 | 9-18 | 48.7 | ELISA/IHA/IFFI | Design: age, sex, level of education |
| Bar ME et al. 1998 [23] | Argentina | Cross-sectional | 1993 | Population | Rural | 132 | 2-79 | 56.1 | IFI/IHA | No |
| De Andrade ALSS et al. 1998 [24] | Brazil | Cross-sectional | March to September 1991 | Population | Rural | 423 | 10.4±1.6 | 39.7 | IFI/IHA/ELISA | Design: age, sex, school |
| Madoery R et al. 1998 [25] | Argentina | Cross-sectional | July to October 1994 | Population | Urban | 983 | 10-69 | ---- | IHA/IF/ELISA | No |
| Rangel-Flores H et al. 2001 [26] | Mexico | Cross-sectional | ---- | Population | Urban | 129 | 2-66 | ---- | ELISA/Western-Blotting | Design: age, sex, same geographic region |
| Borges-Pereira J et al. 2001 [27] | Brazil | Cross-sectional | 1998 | Population | Urban | 382 | 1-60+ | 56.5 | IFI/IHA/ELISA | Design: age, sex |
| Borges-Pereira J et al. 2002 [28] | Brazil | Cross-sectional | ---- | Population | Rural/ Urban | 330 | 1-60+ | 48.1 | IFI/IHA/ELISA/XD/ PCR | No |
| Frédérique Breniére S et al. 2002 [29] | Bolivia | Cross-sectional | 1994 to 1995 | Population | Rural | 232 | 1-45+ | 43.1 | IF/ELISA/PCR | No |
| Coura J et al. 2002 [30] | Brazil | Cross-sectional | ---- | Population | ---- | 193 | 0-60+ | 55.6 | IFI/ELISA | Design: age and sex |
| Rosas F et al. 2002 [31] | Colombia | Cross-sectional | ---- | Population | Rural/ Urban | 405 | >15 | 58.0 | EISA/IFI | Design: age and sex |
| Sosa-Jurado F et al. 2003 [32] | Mexico | Cross-sectional | ---- | Population | Rural | 79 | 6-73 | 82.0 | IFI/ELISA/PCR | Design: age |
| Goldbaum M et al. 2004 [33] | Brazil | Cross-sectional | 1980 | Population | Urban | 921 | 15-50+ | ---- | IF/HA/CFT | Design: age, occupation |
| Chaves AM et al. 2004 [34] | Colombia | Prospective Cohort* | 1999 to 2004 | Blood Donors | Urban | 2130 | 18-50 | 35.9 | ELISA/IHA/IFI | Analysis: age, socioeconomic status, diastolic blood pressure, total cholesterol and glucose intolerance or diabetes |
| Becerril-Flores M et al. 2007 [35] | Mexico | Cross-sectional | ---- | Population | ---- | 214 | 7-64 | 52.7 | ELISA/IHA | No |
| Sánchez Sánchez Y et al. 2007 [36] | Peru | Cross-sectional | 2004 to 2005 | population | Rural | 75 | >15 | 64 | Serology | Design: age and sex |
| Williams-Blangero S et al. 2007 [37] | Brazil | Cross-sectional | ---- | Population | Rural | 1389 | 41.9 | 50.3 | ELISA/HA/IF | No |
| Medrano-Mercado N et al. 2008 [38] | Bolivia | Cross-sectional | 1995 to 1999 | Population | Urban | 1831 | 5-13 | 48.0 | IHA/ELISA | No |
| Borges-Pereira J et al. 2008 [39] | Brazil | Cross-sectional | March 2000 to April 2002 | Population | Rural | 34 | 0-70 | 52.9 | IF/IHA/ELISA/XD/ PCR | Design: age and sex |
| Da Silva E et al. 2010 [40] | Brazil | Cross-sectional | May to August 2007 | Population | Rural | 44 | 2-97 | 56.0 | IHA/ELISA | Design: not specify characteristics |
| Brum-Soares L et al. 2010 [41] | Brazil | Cross-sectional | ---- | Population | ---- | 76 | 7-81 | ---- | IFI/ELISA/ Immunoblot/DX/PCR | No |
| Moretti E et al. 2010 [42] | Argentina | Cross-sectional | ---- | Population | Rural/ Urban | 469 | 0-61+ | ---- | ELISA/IHA | No |
| Ferreira et al. 2011[43] | Brazil | Cross-sectional | 1977 to 1981 | Population | Rural | 4240 | 0-59+ | 60.3 | IFI | Design: age and sex |
| Monteon V et al. 2013 [44] | Mexico | Cross-sectional | ---- | Population | Rural | 128 | 47 | 85.9 | ELISA/IFI | No |
| Ribeiro AL et al. 2013 [45] | Brazil | Prospective cohort* | July 2008 to October 2010 | Population/ Blood Donors | Urban | 987 | 20-60+ | 49.1 | ELISA/HA/IF | Design: site, sex, age, year of blood donation |
| Ribeiro AL et al. 2014 [46] | Brazil | Prospective cohort* | February to May 1997 | Population | ---- | 1462 | 63-74 | 60.9 | IHA/ELISA | Analysis: age, sex, conventional risk factors |
| Molina-Garza Z et al. 2014 [47] | Mexico | Cross-sectional | April 2007 to September 2011 | Population | Rural/ Suburban/Urban | 70 | 17-79 | 59.3 | ELISA/IHA | Design: age, sex, residents of the same localities |
| Yager J et al. 2015 [48] | Bolivia | Cross-sectional | January 2009 to May 2010 | Population | Rural/ Periurban | 604 | 20-60+ | 64.1 | Immunochromatograp-hic/ ELISA/IHA | Analysis: age, serostatus |
| Alroy K et al. 2015 [49] | Peru | Cross-sectional | December 2009 to October 2010 | Population | Rural | 260 | 2-60+ | 58.5 | ELISA/  Inmmunoblot  (TESA-blot) | Design: age and sex |

*ECG abnormalities were reported as base characteristic in a cross-sectional study; ^†^Age of range or mean and standard deviation; ----=Missing data; IFI=Inmunofluorescence Indirect; IHA=Indirect Haemagglutination; IF=Immuofluorescense; DA= Direct Agglutination; CFT= Complement Fixation Test; HA= Haemagglutination; ELISA=Enzyme Linked Immunosorbent Assay; XD=Xenodiagnostic; PCR=Polymerase Chain Reaction.
