# Supplementary material for: Electrocardiographic abnormalities in Chagas disease in the general population: A systematic review and meta-analysis
Source: PLoS Negl Trop Dis. 2018 Jun 13;12(6):e0006567. doi: 10.1371/journal.pntd.0006567 (PMC5999094; doi:10.1371/journal.pntd.0006567)
Supplement: S2 Table — (DOCX) [file pntd.0006567.s006.docx]

| **Lead author, Publication date** | **ECG Abnormalities Definition** |
| --- | --- |
| Pushong E et al. 1964 [1] | Sokolow and Lyon criteria were used to ventricular hypertrophies, and the Goldberger criteria for QTc and QT intervals, considering the ones above 1.09 and 1.08 to be pathological for males and females, respectively. |
| Giraldo Correa LE et al.1965 [2] | ECGs with one or more of the following findings were considered abnormal: extrasystoles, left and right cavity enlargement; complete or incomplete atrioventricular block; complete and incomplete right and left bundle branch block, myocardial and subendocardial lesion, ischemia, overload and infarction. |
| Puigbó JJ et al. 1966 [3] | The following criteria were used in the interpretation of ECGs: ventricular repolarization disorders, cardiac rhythm disorders, disorders of intraventricular conduction, QRS voltage alterations, ventricular hypertrophy, incomplete left bundle branch block, unclassified intraventricular block. The population was divided into three groups: no evidence of heart disease, definite heart disease and doubtful heart disease (ECG with slight abnormalities). |
| Maguire J et al. 1982 [4] | ECGs interpreted according to a modification of the Minnesota Code. ECGs were classified as normal, borderline or abnormal according to criteria. |
| Maguire J et al. 1983 [5] | An amplification of the Minnesota Code was used to classify the ECGs. The amplified code includes criteria for complex rhythm and conduction disturbances adapted from the New York Heart Association and separate criteria for young patients. ECGs with one or more of the following alterations were classified as abnormal: large Q or QS waves, pattern of ventricular hypertrophy (tall precordial R waves) with ST segment and T wave alterations, ST and T alterations of ischemic type, atrioventricular block, ventricular conduction defects; arrhythmias (supraventricular or ventricular tachycardia. multifocal or repetitive ventricular extrasystoles. junctional rhythm. atrial fibrillation or flutter). |
| Baruffa G et al. 1983 [6] | Interpretation of the ECGs was made according to the American Heart Association criteria. ECG abnormalities were classified and grouped as follows: I) alterations of the stimulus formation: sinusal tachycardia (HR ≥100 bpm), sinusal bradycardia (HR < 60 bpm), supraventricular extrasystoles, unifocal and/or multifocal ventricular extrasystoles, flutter and atrial fibrillation and nodal rhythm. II) Alterations of the stimulus conduction: atrioventricular blocks, complete or incomplete branch blocks and intraventricular conduction disturbances. III) ST and T alterations. IV) Overload of cavities. V) Alterations suggestive of necrosis and/or fibrosis and VI) Low voltage QRS. |
| Goldsmith RS et al. 1985 [7] | A cardiologist recorded the rhythm, rate, PQRSTU contours, PR interval, and QRS axis, duration, and configuration of each ECG in accordance with standard criteria but without knowledge of the patient's serologic status. |
| Borges-Pereira and Coura JR. 1986 [8] | ECGs interpretation was based on the New York Heart Association – NYHA, considering normal heart frequencies from 60 to 120 beats per minute. To define the degree of severity of the cardiac lesion among the subjects positive to Chagas, World Health Organization (WHO)/ American Health Organization (PAHO) criteria were used. All ECGs were considered abnormal. |
| Borges-Pereira J and Coura JR. 1987 [9] | ECGs interpretation was based on the New York Heart Association (NYHA), considering as normal heart frequencies between 60 and 120 beats per minute. |
| Acquatella H et al.1987 [10] | Electrocardiographic criteria for abnormality were classified according to the New York Heart Association (NYHA) and the electrocardiographic classifications of normal, borderline, and abnormal for the study of Chagas disease of Maguire et al, were used |
| Kawabata M et al. 1987 [11] | ECGs were analyzed according to a modification of the Minnesota Code. Classification of ECGS as abnormal was based on the following criteria: abnormal Q or QR waves, pattern of ventricular hypertrophy with ST segment and T wave alterations, ST and T alterations of ischemic type, atrioventricular block, ventricular conduction defects and arrhythmias (supraventricular or ventricular tachycardia, multifocal or repetitive ventricular extrasystoles, junctional rhythm, atrial fibrillation or flutter). |
| Weinke TH et al. 1988 [12] | ECGs were coded independently by two cardiologically experienced physicians, by a modified Minnesota Code. Classification of ECGs as abnormal was based on the following criteria: ventricular conduction defects, arrhythmias (repetitive or multifocal extrasystoles. bradycardia. supraventricular or ventricular tachycardia), ST and T alterations of ischemic type, atrioventricular block, abnormal Q or QS waves, pattern of ventricular hypertrophy with ST segment and T wave alterations. |
| Wisnivesky-Colli C et al. 1989 [13] | Electrocardiographic abnormalities were classified as: intraventricular blocks (complete or incomplete; isolated ones or associated with other disturbances), arrhythmias and disturbances in ventricular repolarization. Diagnosis of right bundle branch block (RBBB) and left anterior hemiblock (LAH) were done according to classical criteria and Rosenbaum´s description, respectively. Frequencies lower than 60 bpm were considered as sinusal bradycardia. |
| Arribada C et al. 1990 [14] | The electrocardiographic diagnosis was made according to the “diagnostic criteria for chronic Chagasic cardiopathy”. Any case with symptomatology and ECG alterations was considered as Chronic Chagasic cardiopathy, excluding other cardiopathies with the epidemiologic questionnaires, physical exam, and laboratory findings. |
| Zicker F et al. 1990 [15] | Interpretation of the ECGs traces was conducted independently by two of us (FZ and JCAN) using a classification system based on the Minnesota code adapted for Chagas's disease. Traces were classified as normal, borderline or abnormal, according to the criteria of Maguire et al. An ECG was classified as abnormal in the presence of one or more of: large Q or QS waves; pattern of ventricular hypertrophy (tall precordial R waves) with ST segment and T wave alteration; A-V block; ventricular conduction defects; complex arrhythmias; ventricular premature beats when present in 10% or more of recorded cycles or when multifocal or bigeminy; and sinus bradycardia (<50 bpm) associated with extrasystoles or primary and diffuse changes in ventricular repolarization. |
| Pless M et al. 1992 [16] | Electrocardiographic criteria for abnormalities were based on standard definitions. |
| Goldsmith RS et al. 1992 [17] | A cardiologist recorded the rhythm, rate, PQRSTU contours, PR interval, and QRS axis, duration and configuration of each ECG in accordance with standard criteria but without knowledge of the patient's serologic status. |
| Dias JC. 1993 [18] | ECGs were analyzed according to Laranja et all criteria, and Faria systematization. |
| Morini J et al. 1994 [19] | Non-specified. |
| Gianella A et al. 1994 [20] | ECGs were interpreted following the criteria of the New York Heart Association (cardiac rhythm alterations, ventricular repolarization changes, and intraventricular conduction disorders). |
| Rivera BT et al. 1995 [21] | Non-specified. |
| Aguilera M et al. 1996 [22] | ECGs were blind-interpreted by criteria of Panamerican Health Organization for chronic cardiomyopathy Chagas. |
| Bar ME et al. 1998 [23] | ECGs alterations were classified according to Tranchesi. |
| De Andrade ALSS et al. 1998 [24] | ECGs traces were interpreted by two independent cardiologist readers following a coding system adapted for Chagas’ disease. An ECG was classified as abnormal in the presence of at least one of the following: large Q or QS waves, pattern of ventricular hypertrophy (tall precordial R waves) with ST segment and T wave alteration, A-V block, ventricular conduction defects, complex arrhythmia, ventricular premature beats when present in 10% or more of recorded cycles or when multifocal or bigeminy, and sinus bradycardia (<50 bpm) associated with extrasystoles or primary and diffuse changes in ventricular repolarization. Complete bundle branch block was defined as rR’ or R waves in V1 with a duration ≥ 0.12 sec. |
| Madoery R et al. 1998 [25] | ECGs were interpreted by Consejo Argentino de la Enfermedad de Chagas y Cardiopatías Infecciosas. |
| Rangel-Flores H et al. 2001 [26] | ECGs were classified as normal or abnormal according to the New York Heart Association criteria. |
| Borges-Pereira J et al. 2001 [27] | ECGs analysis was made by two observers, considering heart frequencies 60 to 120 bpm and sinusal rhythm as normal. |
| Borges-Pereira J et al. 2002 [28] | ECGs analysis was made by two observers, considering heart frequencies 60 to 120 bpm and sinusal rhythm as normal. |
| Frédérique Breniére S et al. 2002 [29] | ECGs showing atrioventricular block, incomplete and complete right bundle branch block, left anterior hemiblock, multifocal ventricular extrasystole, or junctional rhythm were considered abnormal. |
| Coura J et al. 2002 [30] | Non-specified. |
| Rosas F et al. 2002 [31] | ECGs were interpreted by criteria of group of experts in epidemiological studies of Chagas diseases in 1998, according methodology proposed by Lazzari. |
| Sosa-Jurado F et al. 2003 [32] | The criteria for ECGs interpretation were those utilized by the Department of Electrocardiography and Electrophysiology of the Instituto Nacional de Cardiología based on the deductive method of ECG interpretation. Special attention was given to the presence of arrhythmias, A-V heart block, bundle branch blocks, fascicular block, atrial and ventricular hypertrophy, and repolarization abnormalities with change in the ventricular gradient. |
| Goldbaum M et al. 2004 [33] | Non-specified. |
| Chaves AM et al. 2004 [34] | ECGs were initially classified as normal (Yes/No). Whenever abnormality was detected, at least one of the following findings had to be classified: presence/absence of “definitive” rhythm or conduction abnormalities or other findings, as has been defined for the context of Chagas Disease. These included RBBB, ventricular extrasystoles, atrioventricular blocks, and auricular fibrillation. Traces suspected (or “with a pattern”), of intraventricular conduction alterations (with complex QRS of compatible morphology, but duration below 120 ms, or PR intervals consistently not exceeding 200ms) or ectopic supraventricular heart beats. Findings reported as “other abnormalities” included signs of ventricular hypertrophy, alterations of T wave or Q wave pathological. |
| Becerril-Flores M et al. 2007 [35] | Classification of ECGs results as normal or abnormal was made according to the deductive method of ECG interpretation and the criteria of our medical personnel. |
| Sánchez Sánchez Y et al. 2007 [36] | Non-specified. |
| Williams-Blangero S et al. 2007 [37] | Non-specified. |
| Medrano-Mercado N et al. 2008 [38] | Non-specified. |
| Borges-Pereira J et al. 2008 [39] | Reading of ECGs traces used Minessota code adapted for chronic Chagas cardiopathy. |
| Da Silva E et al. 2010 [40] | Non-specified. ECGs rhythm and frequencies were analyzed. |
| Brum-Soares L et al. 2010 [41] | ECGs were interpreted according to the New York Heart Association criteria. ECGs analyses were made by two blinded cardiologists, according to Minnesota code, adapted for Chagas disease. The following alterations were considered as suggestive of Chagas disease: right complete branch block associated or not with anterior left hemiblock, ventricular extrasystoles, sinusal bradycardia (HR <50 bpm), second degree atrioventricular block, primary alterations of ventricular repolarization, presence of electrically inactive areas, sinusal node dysfunction, non-sustained ventricular tachycardia, atrial fibrillation, total atrioventricular block and left branch block. |
| Moretti E et al. 2010 [42] | Electrocardiographic abnormalities were reported according to criteria established by Lazzari et al 1998. |
| Ferreira et al. 2011 [43] | ECGs were interpreted according to the Buenos Aires Code. |
| Monteon V et al. 2013 [44] | ECGs were analyzed by a cardiologist; special care was given to rhythm, conduction, and isquemia findings. |
| Ribeiro AL et al. 2013 [45] | ECGs were classified by Minnesota Code Criteria. In this study, major and minor ECG abnormalities were defined as set out in Prineas et al, modified to include ECG abnormalities typical of Chagas cardiomyopathy with prognostic significance, as frequent supraventricular or ventricular premature beats. |
| Ribeiro AL et al. 2014 [46] | Classified by the Minnesota Code Criteria. In this study. major and minor ECG abnormalities were defined as set out in Prineas et al. modified to include ECG abnormalities typical of Chagas cardiomyopathy with prognostic significance, as frequent supraventricular or ventricular premature beats. |
| Molina-Garza Z et al. 2014 [47] | ECGs results (normal or abnormal) were analyzed and interpreted by blinded evaluators, and ECG results were classified according to the deductive method of ECG interpretation and criteria by our medical personnel. |
| Yager J et al. 2015 [48] | ECGs were coded and interpreted according to established criteria. |
| Alroy K et al. 2015 [49] | An ECG was considered to have abnormalities consistent with Chagas cardiomyopathy in the presence of one or more of the following: atrial fibrillation/flutter, junctional rhythm, ventricular tachycardia (sustained or non-sustained), ventricular extrasystoles (multiform, paired, or salvos), sinus node dysfunction, sinus bradycardia (<50 bpm), second degree AV block (type I or type II), third degree AV block, AV disassociation, left or right bundle branch block, left anterior or left posterior fascicular block, or trifascicular block. |
