# Supplementary material for: Electrocardiographic abnormalities in Chagas disease in the general population: A systematic review and meta-analysis
Source: PLoS Negl Trop Dis. 2018 Jun 13;12(6):e0006567. doi: 10.1371/journal.pntd.0006567 (PMC5999094; doi:10.1371/journal.pntd.0006567)
Supplement: S3 Table — (DOCX) [file pntd.0006567.s007.docx]

| **Characteristics** | **Number or n (%)** |
| --- | --- |
| **Eligible studies**  No. of unique studies | 49 |
| **Participants**  Total  CD (Positive)  Non-CD (Negative)  Range of age (years)  Women proportion (n=38) | 34,023  12,276  21,747  0-97  54.4% |
| **Publication Year**  1964-1989  1990-2000  2001-2010  2011-2015 | 13 (26.53)  12 (24.49)  17 (34.69)  7 (14.29) |
| **Location**  Brazil  Mexico  Argentina  Bolivia  Colombia  Chile  Peru  Venezuela  Others (Ecuador y Nicaragua)  **Design**  Cross-sectional  Cohort  **Area**  Rural  Urban  Rural/Urban  Rural/Periurban  Rural/Suburban/Urban  Non-specified  **Risk of Bias**  High  Medium  Low | 19 (38.78)  7 (14.29)  6 (12.24)  6 (12.24)  3 (6.12)  2 (4.08)  2 (4.08)  2 (4.08)  2 (4.08)  43 (87.76)  6 (12.24)  22 (44.90)  11 (22.45)  5 (10.20)  2 (4.08)  1 (2.04)  8 (16.33)  11 (22.45)  28 (57.14)  10 (20.41) |

Values are number of studies (%) or range. CD=Chagas disease.
