# Supplementary material for: Electrocardiographic abnormalities in Chagas disease in the general population: A systematic review and meta-analysis
Source: PLoS Negl Trop Dis. 2018 Jun 13;12(6):e0006567. doi: 10.1371/journal.pntd.0006567 (PMC5999094; doi:10.1371/journal.pntd.0006567)
Supplement: S4 Table — (DOCX) [file pntd.0006567.s008.docx]

| **Characteristics** | **Number of studies** | **Total CD with CAs** | **CRBBB CD** | **Total Non-CD**  **with CAs** | **CRBBB**  **Non-CD** | **OR (95% CI)** | **Heterogeneity** | | **p-value Cochran’s Q** | **p-value Meta-regression*** |
| --- | --- | --- | --- | --- | --- | --- | --- | --- | --- | --- |
|  |  |  |  |  |  |  | **I^2^ (%)** | **p-value** |  |  |
| **Publication year** |  |  |  |  |  |  |  |  |  |  |
| 1983-2000 | 14 | 1,525 | 409 | 2,027 | 76 | 4.97 (2.68-9.24) | 61.3 | 0.001 | 0.766 | 0.723 |
| 2001-2015 | 10 | 1,111 | 301 | 1,333 | 96 | 4.20 (2.11-8.37) | 71.1 | 0.000 |  |  |
| **Location** |  |  |  |  |  |  |  |  |  |  |
| Brazil | 10 | 1,691 | 466 | 2,349 | 77 | 8.43 (5.21-13.62) | 42.6 | 0.074 | 0.000 | 0.012 |
| Argentina | 4 | 169 | 21 | 150 | 11 | 1.31 (0.22-7.73) | 69.8 | 0.019 |  |  |
| Mexico | 3 | 102 | 23 | 56 | 2 | 5.02 (1.25-20.10) | 0.0 | 0.562 |  |  |
| Bolivia | 2 | 121 | 6 | 199 | 1 | 4.44 (0.51-38.89) | 0.0 | 0.328 |  |  |
| Colombia | 2 | 183 | 58 | 267 | 56 | 1.80 (1.15-2.81) | 0.0 | 0.591 |  |  |
| Chile | 1 | 51 | 5 | 197 | 12 | 1.68 (0.56-4.99) | ---- | ---- |  |  |
| Peru | 1 | 10 | 1 | 13 | 0 | 2.78 (0.08-92.10) | ---- | ---- |  |  |
| Venezuela | 1 | 309 | 130 | 129 | 12 | 7.08 (3.75-13.37) | ---- | ---- |  |  |
| **Design** |  |  |  |  |  |  |  |  |  |  |
| Cross-sectional | 20 | 1,973 | 530 | 2,420 | 98 | 4.61 (2.74-7.78) | 61.0 | 0.000 | 0.479 | 0.993 |
| Cohort | 4 | 663 | 180 | 940 | 74 | 4.65 (1.68-12.84) | 80.8 | 0.001 |  |  |
| **Area** |  |  |  |  |  |  |  |  |  |  |
| Rural | 9 | 593 | 74 | 1,602 | 56 | 2.09 (0.95-4.60) | 45.2 | 0.067 | 0.044 | 0.024 |
| Urban | 8 | 885 | 294 | 611 | 54 | 9.01 (3.45-23.55) | 72.6 | 0.001 |  |  |
| **Number of participants**^†^ |  |  |  |  |  |  |  |  |  |  |
| ≤100 | 1 | 10 | 1 | 13 | 0 | 2.78 (0.08-92.10) | ---- | ---- | 0.001 | 0.048 |
| 101-1000 | 13 | 627 | 113 | 695 | 42 | 2.49 (1.28-4.83) | 37.8 | 0.082 |  |  |
| >1000 | 10 | 1,999 | 596 | 2,652 | 129 | 6.90 (4.12-11.55) | 71.0 | 0.000 |  |  |
| **Age of participants** |  |  |  |  |  |  |  |  |  |  |
| All ages | 14 | 1,702 | 448 | 2,561 | 101 | 5.04 (3.26-7.80) | 42.2 | 0.048 | 0.007 | 0.721 |
| ≥ 10 years | 9 | 918 | 253 | 789 | 69 | 4.36 (1.78-10.66) | 75.4 | 0.000 |  |  |
| Only children | 1 | 16 | 9 | 10 | 1 | 11.57 (1.2-114.3) | ---- | ---- |  |  |
| **Definition of CAs** |  |  |  |  |  |  |  |  |  |  |
| Specific definitions | 21 | 2,422 | 652 | 3,208 | 163 | 4.76 (3.11-7.27) | 59.1 | 0.000 | 0.682 | 0.553 |
| Non-specified/no clear | 3 | 214 | 58 | 152 | 8 | 2.87 (0.17-48.48) | 87.4 | 0.000 |  |  |
| **Test for the diagnoses CD** |  |  |  |  |  |  |  |  |  |  |
| One test for CD | 4 | 491 | 76 | 1,360 | 34 | 4.07 (2.49-6.67) | 11.6 | 0.335 | 0.672 | 0.610 |
| More one test for CD | 20 | 2,145 | 634 | 2,000 | 137 | 4.41 (2.67-7.29) | 69.0 | 0.000 |  |  |
| **Confounders adjustment**^‡^ |  |  |  |  |  |  |  |  |  |  |
| Yes | 8 | 568 | 190 | 338 | 23 | 7.29 (2.55-20.88) | 66.7 | 0.004 | 0.861 | 0.251 |
| No | 16 | 2,068 | 520 | 3,022 | 148 | 3.92 (2.39-6.43) | 65.7 | 0.000 |  |  |
| **Risk of bias** |  |  |  |  |  |  |  |  |  |  |
| High | 4 | 114 | 22 | 65 | 4 | 1.70 (0.24-11.99) | 31.4 | 0.224 | 0.148 | 0.521 |
| Medium | 16 | 2,214 | 584 | 2,959 | 120 | 4.76 (2.92-7.79) | 66.1 | 0.000 |  |  |
| Low | 4 | 308 | 104 | 336 | 47 | 5.99 (1.52-23.64) | 75.7 | 0.006 |  |  |

*****p-value for heterogeneity was evaluated using random-effects meta-regression; ^†^Total positive and negative for Chagas disease; ^‡^Adjusted by confounders as age, sex and others in design. CD= Chagas disease; CAs= ECG abnormalities; CRBBB= complete right bundle branch block; OR=odds ratio.
