# Supplementary material for: Electrocardiographic abnormalities in Chagas disease in the general population: A systematic review and meta-analysis
Source: PLoS Negl Trop Dis. 2018 Jun 13;12(6):e0006567. doi: 10.1371/journal.pntd.0006567 (PMC5999094; doi:10.1371/journal.pntd.0006567)
Supplement: S5 Table — (DOCX) [file pntd.0006567.s009.docx]

| **Characteristics** | **Number of studies** | **Total CD with CAs** | **LAFB CD** | **Total Non-CD**  **with CAs** | **LAFB Non-CD** | **OR (95% CI)** | **Heterogeneity** | | **p-value Cochran’s Q** | **p-value Meta-regression*** |
| --- | --- | --- | --- | --- | --- | --- | --- | --- | --- | --- |
|  |  |  |  |  |  |  | **I^2^ (%)** | **p-value** |  |  |
| **Publication year** |  |  |  |  |  |  |  |  |  |  |
| 1983-2000 | 17 | 1,572 | 428 | 2,056 | 270 | 1.37 (0.92-2.04) | 50.7 | 0.009 | 0.595 | 0.277 |
| 2001-2015 | 13 | 2,347 | 306 | 1,984 | 149 | 1.89 (1.22-2.92) | 60.5 | 0.002 |  |  |
| **Location** |  |  |  |  |  |  |  |  |  |  |
| Brazil | 15 | 2,946 | 506 | 3,022 | 273 | 1.90 (1.24-2.90) | 69.8 | 0.000 | 0.245 | 0.538 |
| Argentina | 4 | 169 | 20 | 150 | 12 | 1.43 (0.54-3.79) | 26.5 | 0.253 |  |  |
| Bolivia | 2 | 121 | 23 | 199 | 9 | 1.91 (0.56-6.50) | 26.9 | 0.242 |  |  |
| Colombia | 2 | 183 | 39 | 267 | 43 | 1.93 (1.16-3.22) | 0.0 | 0.773 |  |  |
| Chile | 2 | 73 | 11 | 197 | 37 | 0.77 (0.34-1.77) | 0.0 | 0.404 |  |  |
| Mexico | 2 | 94 | 4 | 52 | 6 | 0.29 (0.07-1.23) | 0.0 | 0.680 |  |  |
| Nicaragua | 1 | 14 | 1 | 11 | 1 | 0.77 (0.04-13.87) | ---- | ---- |  |  |
| Peru | 1 | 10 | 2 | 13 | 3 | 0.83 (0.11-6.26) | ---- | ---- |  |  |
| Venezuela | 1 | 309 | 128 | 129 | 36 | 1.83 (1.17-2.85) | ---- | ---- |  |  |
| **Design** |  |  |  |  |  |  |  |  |  |  |
| Cross-sectional | 25 | 3,001 | 585 | 2,944 | 332 | 1.50 (1.11-2.01) | 42.4 | 0.014 | 0.026 | 0.404 |
| Cohort | 5 | 918 | 149 | 1,096 | 88 | 1.78 (0.83-3.84) | 75.7 | 0.002 |  |  |
| **Area** |  |  |  |  |  |  |  |  |  |  |
| Rural | 12 | 1,582 | 169 | 2,104 | 228 | 0.95 (0.63-1.42) | 35.4 | 0.107 | 0.000 | 0.001 |
| Urban | 9 | 1,143 | 339 | 781 | 101 | 3.11 (1.73-5.58) | 67.2 | 0.002 |  |  |
| **Number of participants**^†^ |  |  |  |  |  |  |  |  |  |  |
| ≤100 | 1 | 10 | 2 | 13 | 3 | 0.83 (0.11-6.26) | ---- | ---- | 0.786 | 0.428 |
| 101-1000 | 18 | 946 | 164 | 902 | 91 | 1.20 (0.66-2.16) | 57.0 | 0.002 |  |  |
| >1000 | 11 | 2,963 | 568 | 3,125 | 326 | 1.78 (1.32-2.39) | 56.8 | 0.010 |  |  |
| **Age of participants** |  |  |  |  |  |  |  |  |  |  |
| All ages | 16 | 2,213 | 380 | 2,381 | 267 | 1.45 (0.96-2.17) | 53.8 | 0.006 | 0.247 | 0.447 |
| ≥ 10 years | 12 | 1,668 | 350 | 1,649 | 151 | 1.83 (1.20-2.78) | 59.8 | 0.004 |  |  |
| Only children | 2 | 38 | 4 | 10 | 1 | 0.40 (0.03-5.88) | 0.0 | 0.483 |  |  |
| **Definition of CAs** |  |  |  |  |  |  |  |  |  |  |
| Specific definitions | 25 | 3,680 | 713 | 3,859 | 403 | 1.70 (1.25-2.32) | 60.5 | 0.000 | 0.167 | 0.229 |
| Non-specified/no clear | 5 | 239 | 21 | 181 | 17 | 1.01 (0.50-2.03) | 0.0 | 0.972 |  |  |
| **Test for the diagnoses CD** |  |  |  |  |  |  |  |  |  |  |
| One test for CD | 5 | 1,455 | 145 | 1,833 | 175 | 1.13 (0.58-2.20) | 73.4 | 0.005 | 0.066 | 0.213 |
| More one test for CD | 25 | 2,464 | 589 | 2,207 | 245 | 1.78 (1.29-2.44) | 46.3 | 0.006 |  |  |
| **Confounders adjustment**^‡^ |  |  |  |  |  |  |  |  |  |  |
| Yes | 12 | 1,826 | 238 | 992 | 71 | 1.98 (0.98-4.00) | 70.0 | 0.000 | 0.910 | 0.259 |
| No | 18 | 2,093 | 496 | 3,048 | 349 | 1.50 (1.14-1.96) | 35.6 | 0.067 |  |  |
| **Risk of bias** |  |  |  |  |  |  |  |  |  |  |
| High | 5 | 131 | 21 | 87 | 15 | 0.88 (0.30-2.57) | 35.2 | 0.187 | 0.056 | 0.258 |
| Medium | 17 | 2,225 | 529 | 2,977 | 311 | 1.79 (1.36-2.37) | 34.7 | 0.079 |  |  |
| Low | 8 | 1,563 | 184 | 976 | 94 | 1.38 (0.66-2.91) | 73.7 | 0.000 |  |  |

*****p-value for heterogeneity was evaluated using random-effects meta-regression; ^†^Total positive and negative for Chagas disease; ^‡^ Adjusted by confounders as age, sex and others in design. CD= Chagas disease; CAs=ECG abnormalities; LAFB=left anterior fascicular block; OR=odds ratio.
