# Supplementary material for: Electrocardiographic abnormalities in Chagas disease in the general population: A systematic review and meta-analysis
Source: PLoS Negl Trop Dis. 2018 Jun 13;12(6):e0006567. doi: 10.1371/journal.pntd.0006567 (PMC5999094; doi:10.1371/journal.pntd.0006567)
Supplement: S6 Table — (DOCX) [file pntd.0006567.s010.docx]

| **Characteristics** | **Number of studies** | **Total CD with CAs** | **VE CD** | **Total Non-CD**  **with CAs** | **VE Non-CD** | **OR (95% CI)** | **Heterogeneity** | | **p-value Cochran’s Q** | **p-value**  **Meta-regression*** |
| --- | --- | --- | --- | --- | --- | --- | --- | --- | --- | --- |
|  |  |  |  |  |  |  | **I^2^ (%)** | **p-value** |  |  |
| **Publication year** |  |  |  |  |  |  |  |  |  |  |
| 1964-2000 | 16 | 1,576 | 386 | 2,630 | 278 | 1.57 (0.95-2.59) | 65.5 | 0.000 | 0.721 | 0.798 |
| 2001-2015 | 9 | 1,566 | 202 | 1,119 | 89 | 1.66 (1.01-2.74) | 48.8 | 0.048 |  |  |
| **Location** |  |  |  |  |  |  |  |  |  |  |
| Brazil | 11 | 2,177 | 378 | 2,162 | 264 | 1.39 (0.85-2.29) | 74.6 | 0.000 | 0.166 | 0.672 |
| Argentina | 5 | 211 | 34 | 734 | 33 | 3.01 (0.90-10.06) | 47.6 | 0.106 |  |  |
| Mexico | 3 | 102 | 16 | 56 | 3 | 3.43 (0.92-12.79) | 0.0 | 0.964 |  |  |
| Colombia | 2 | 183 | 16 | 267 | 20 | 1.32 (0.48-3.60) | 40.7 | 0.194 |  |  |
| Chile | 1 | 51 | 2 | 197 | 5 | 1.57 (0.30-8.32) | ---- | ---- |  |  |
| Bolivia | 1 | 95 | 1 | 193 | 0 | 4.10 (0.14-123.18) | ---- | ---- |  |  |
| Nicaragua | 1 | 14 | 0 | 11 | 2 | 0.17 (0.01-4.14) | ---- | ---- |  |  |
| Venezuela | 1 | 309 | 141 | 129 | 40 | 1.87 (1.21-2.89) | ---- | ---- |  |  |
| **Design** |  |  |  |  |  |  |  |  |  |  |
| Cross-sectional | 22 | 2,963 | 562 | 3,495 | 349 | 1.54 (1.06-2.26) | 63.1 | 0.000 | 0.202 | 0.403 |
| Cohort | 3 | 179 | 26 | 254 | 18 | 2.23 (1.11-4.47) | 0.0 | 0.731 |  |  |
| **Area** |  |  |  |  |  |  |  |  |  |  |
| Rural | 9 | 1,530 | 191 | 2,075 | 217 | 1.14 (0.61-2.14) | 62.8 | 0.006 | 0.018 | 0.225 |
| Urban | 9 | 896 | 214 | 629 | 73 | 1.98 (1.09-3.62) | 65.3 | 0.003 |  |  |
| **Number of participants**^†^ |  |  |  |  |  |  |  |  |  |  |
| 101-1000 | 14 | 621 | 90 | 726 | 47 | 1.65 (0.66-4.12) | 64.7 | 0.000 | 0.989 | 0.809 |
| >1000 | 11 | 2,521 | 498 | 3,023 | 320 | 1.60 (1.16-2.20) | 55.4 | 0.013 |  |  |
| **Age of participants** |  |  |  |  |  |  |  |  |  |  |
| All ages | 16 | 2,223 | 415 | 2,955 | 294 | 1.65 (1.15-2.38) | 45.8 | 0.024 | 0.535 | 0.561 |
| ≥ 10 years | 9 | 919 | 173 | 794 | 73 | 1.37 (0.62-3.06) | 74.4 | 0.000 |  |  |
| **Definition of CAs** |  |  |  |  |  |  |  |  |  |  |
| Specific definitions | 20 | 2,871 | 542 | 2,997 | 326 | 1.53 (1.06-2.22) | 61.0 | 0.000 | 0.092 | 0.468 |
| Non-specified/no clear | 5 | 271 | 46 | 752 | 41 | 2.13 (0.67-6.79) | 47.6 | 0.106 |  |  |
| **Test for the diagnoses CD** |  |  |  |  |  |  |  |  |  |  |
| One test for CD | 5 | 1,487 | 179 | 2,404 | 239 | 0.84 (0.47-1.50) | 68.0 | 0.014 | 0.000 | 0.010 |
| More one test for CD | 20 | 1,655 | 409 | 1,345 | 128 | 2.14 (1.44-3.18) | 44.7 | 0.017 |  |  |
| **Confounders adjustment**^‡^ |  |  |  |  |  |  |  |  |  |  |
| Yes | 9 | 1,531 | 212 | 817 | 80 | 1.66 (0.89-3.09) | 63.1 | 0.005 | 0.513 | 0.010 |
| No | 16 | 1,611 | 376 | 2,932 | 287 | 1.62 (1.03-2.56) | 59.6 | 0.001 |  |  |
| **Risk of bias** |  |  |  |  |  |  |  |  |  |  |
| High | 4 | 115 | 35 | 70 | 27 | 0.57 (0.08-3.93) | 71.9 | 0.014 | 0.009 | 0.036 |
| Medium | 16 | 1,757 | 410 | 2,869 | 269 | 1.94 (1.30-2.89) | 52.7 | 0.007 |  |  |
| Low | 5 | 1,270 | 143 | 810 | 70 | 1.58 (0.89-2.81) | 47.0 | 0.110 |  |  |

*****p-value for heterogeneity was evaluated using random-effects meta-regression; ^†^Total positive and negative for Chagas disease; ^‡^ Adjusted by confounders as age, sex and others in design. CD= Chagas disease; CAs=ECG abnormalities; VE=ventricular extrasystoles; OR=odds ratio.
