# Supplementary material for: Electrocardiographic abnormalities in Chagas disease in the general population: A systematic review and meta-analysis
Source: PLoS Negl Trop Dis. 2018 Jun 13;12(6):e0006567. doi: 10.1371/journal.pntd.0006567 (PMC5999094; doi:10.1371/journal.pntd.0006567)
Supplement: S7 Table — (DOCX) [file pntd.0006567.s011.docx]

| **Variable** | **p-value of Egger´s Test** |
| --- | --- |
| **Overall** |  |
| Prevalence of ECG abnormalities | 0.202 |
| **Ventricular conduction defects** |  |
| Incomplete right bundle branch block | 0.478 |
| Complete right bundle branch block | 0.962 |
| Incomplete left bundle branch block | 0.990 |
| Complete left bundle branch block | 0.565 |
| Left anterior fascicular block | 0.601 |
| Left posterior fascicular block | 0.552 |
| Complete Right bundle branch block and left anterior fascicular block | 0.671 |
| **Atrioventricular block** |  |
| First degree left atrioventricular block | 0.300 |
| Second degree left atrioventricular block | 0.766 |
| Third degree left atrioventricular block | 0.592 |
| **Arrhythmias** |  |
| Atrial fibrillation | 0.568 |
| Atrial fibrillation or flutter | 0.085 |
| Ventricular extrasystoles | 0.352 |
| Supraventricular extrasystoles | 0.496 |
| **Other** |  |
| Low voltage QRS | 0.715 |
