# Supplementary material for: Electrocardiographic abnormalities in Chagas disease in the general population: A systematic review and meta-analysis
Source: PLoS Negl Trop Dis. 2018 Jun 13;12(6):e0006567. doi: 10.1371/journal.pntd.0006567 (PMC5999094; doi:10.1371/journal.pntd.0006567)
Supplement: S8 Table — (DOCX) [file pntd.0006567.s012.docx]

| **Characteristics** | **Number of studies (n)** | **True Positive (n)** | **False Positive (n)** | **False Negative (n)** | **True Negative (n)** | **Sensitivity (%)**  **95% CI** | **Specificity (%)**  **95% CI** | **Positive Predictive Value (%) 95% CI** | **Negative Predictive Value (%) 95% CI** |
| --- | --- | --- | --- | --- | --- | --- | --- | --- | --- |
| ECG abnormalities | 49 | 4922 | 5243 | 7354 | 16504 | 40.09  (39.23-40.97) | 75.89  (75.32-76.46) | 48.42  (47.62-49.22) | 69.18  (68.83-69.52) |
| **Specific ECG abnormalities** | | | | | | | | | |
| Ventricular extrasystoles | 25 | 588 | 366 | 2554 | 3383 | 18.71  (17.36-20.12) | 90.24  (89.24-91.17) | 61.64  (58.72-64.47) | 56.98  (56.50-57.47) |
| LAFB | 30 | 734 | 419 | 3185 | 3625 | 18.73  (17.52-19.99) | 89.63  (88.65-90.55) | 63.66  (61.04-66.20) | 53.20  (52.75-53.66) |
| Atrial fibrillation or flutter | 13 | 78 | 40 | 2576 | 3710 | 2.94  (2.33-3.65) | 98.93  (98.55-99.24) | 66.10  (57.20-74.00) | 59.02  (58.84-59.20) |
| The first AV-B | 21 | 161 | 77 | 2951 | 3380 | 5.17  (4.42-6.01) | 97.77  (97.22-98.24) | 67.65  (61.55-73.20) | 53.39  (53.15-53.63) |
| Complete RBBB | 24 | 710 | 169 | 1926 | 3191 | 26.93  (25.25-28.67) | 94.97  (94.18-95.68) | 80.77  (78.17-83.13) | 62.36  (61.79-62.93) |
| Complete RBBB+LAFB | 15 | 167 | 34 | 1724 | 1574 | 8.83 (7.59-10.20) | 97.89 (97.06-98.53) | 83.08 (77.36-87.59) | 47.73 (47.33-48.12) |

CI= Confidence interval; ECG=Electrocardiogram; LAFB=Left anterior fascicular block; AV- B=Atrioventricular block; RBBB=Right bundle branch block.
