# Supplementary figures and images for: Electrocardiographic abnormalities in Chagas disease in the general population: A systematic review and meta-analysis
Source: PLoS Negl Trop Dis. 2018 Jun 13;12(6):e0006567. doi: 10.1371/journal.pntd.0006567 (PMC5999094; doi:10.1371/journal.pntd.0006567)

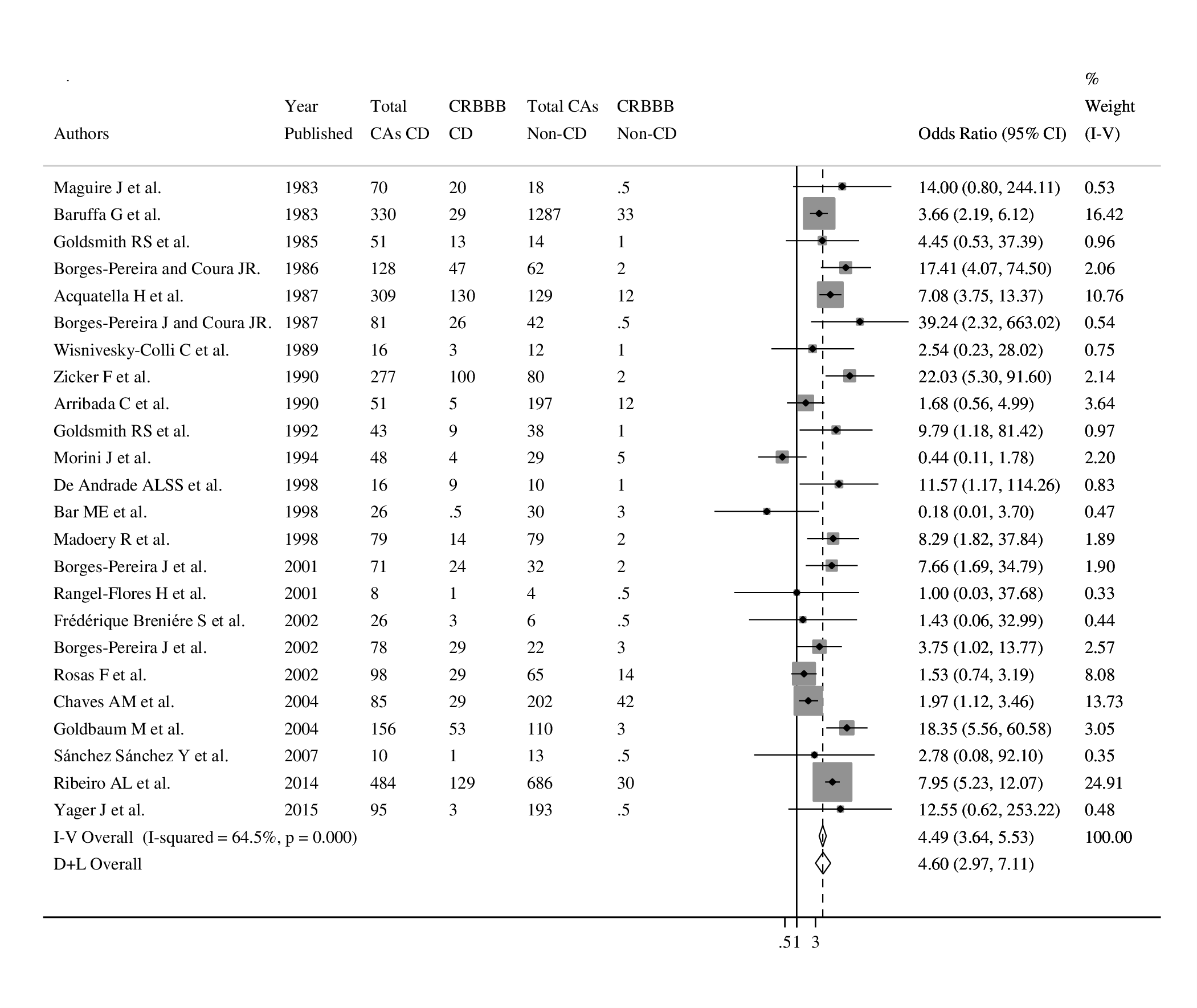

Supplement: S1 Fig — (TIF) [file pntd.0006567.s013.tif]

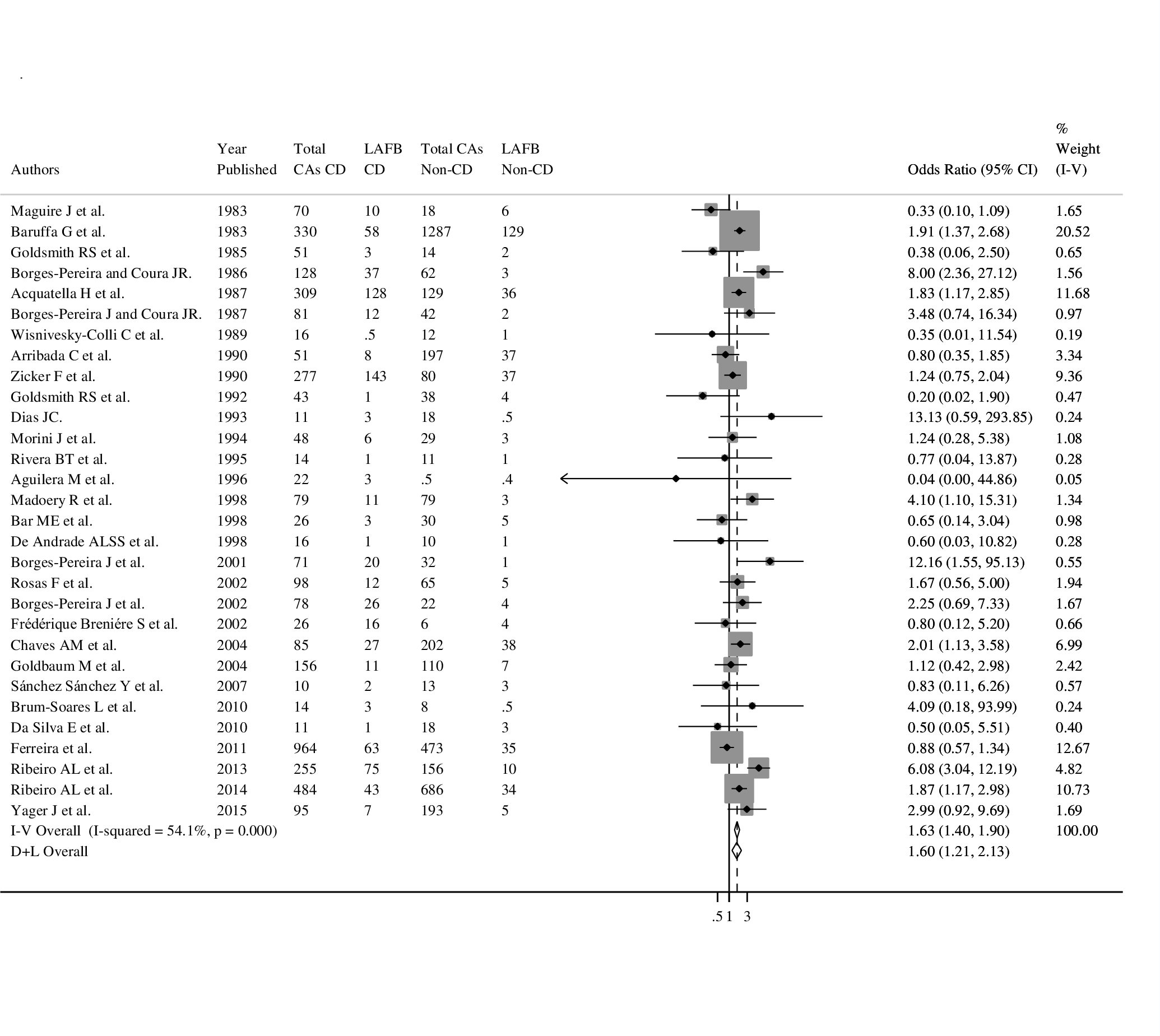

Supplement: S2 Fig — (TIF) [file pntd.0006567.s014.tif]

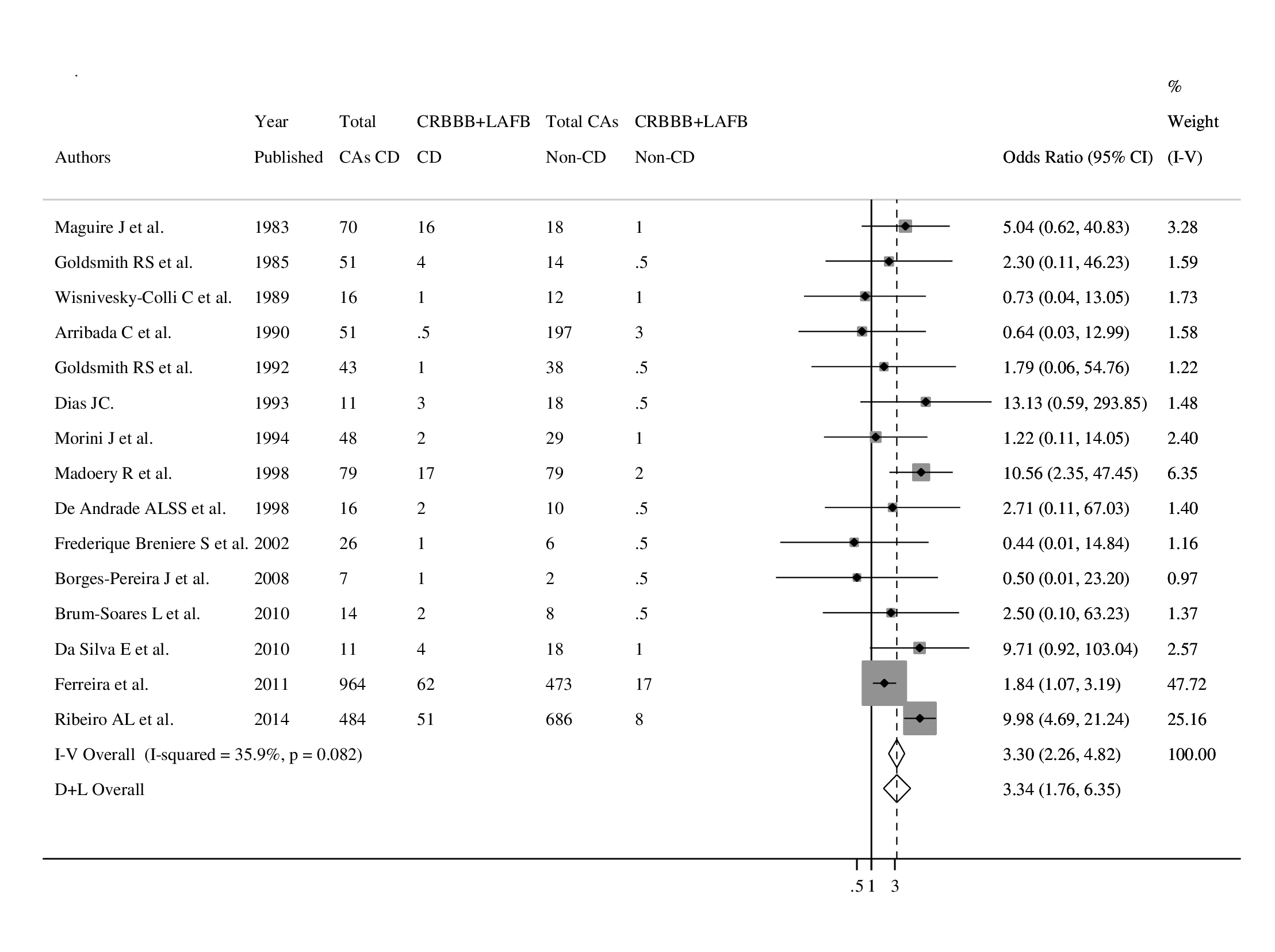

Supplement: S3 Fig — (TIF) [file pntd.0006567.s015.tif]

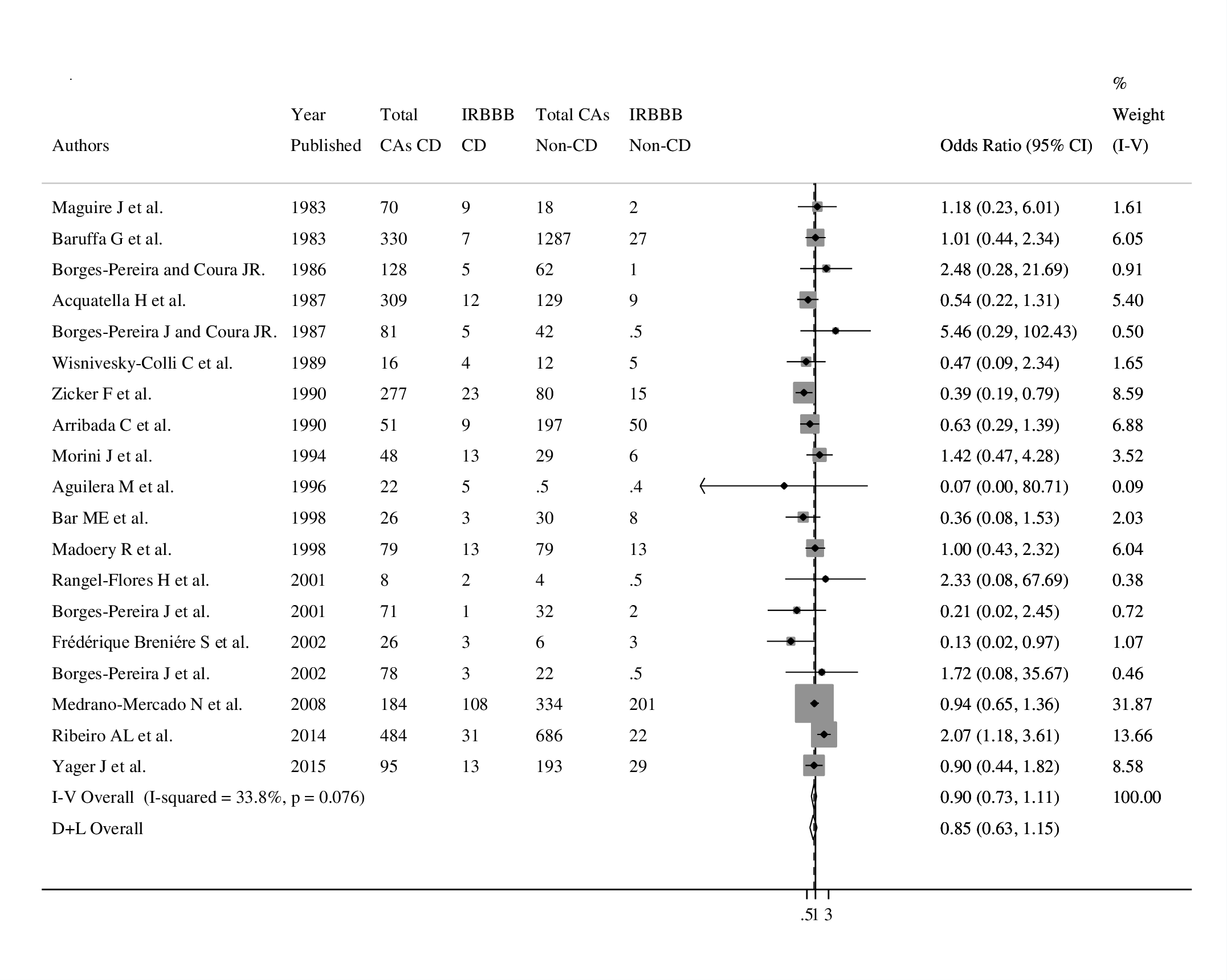

Supplement: S4 Fig — (TIF) [file pntd.0006567.s016.tif]

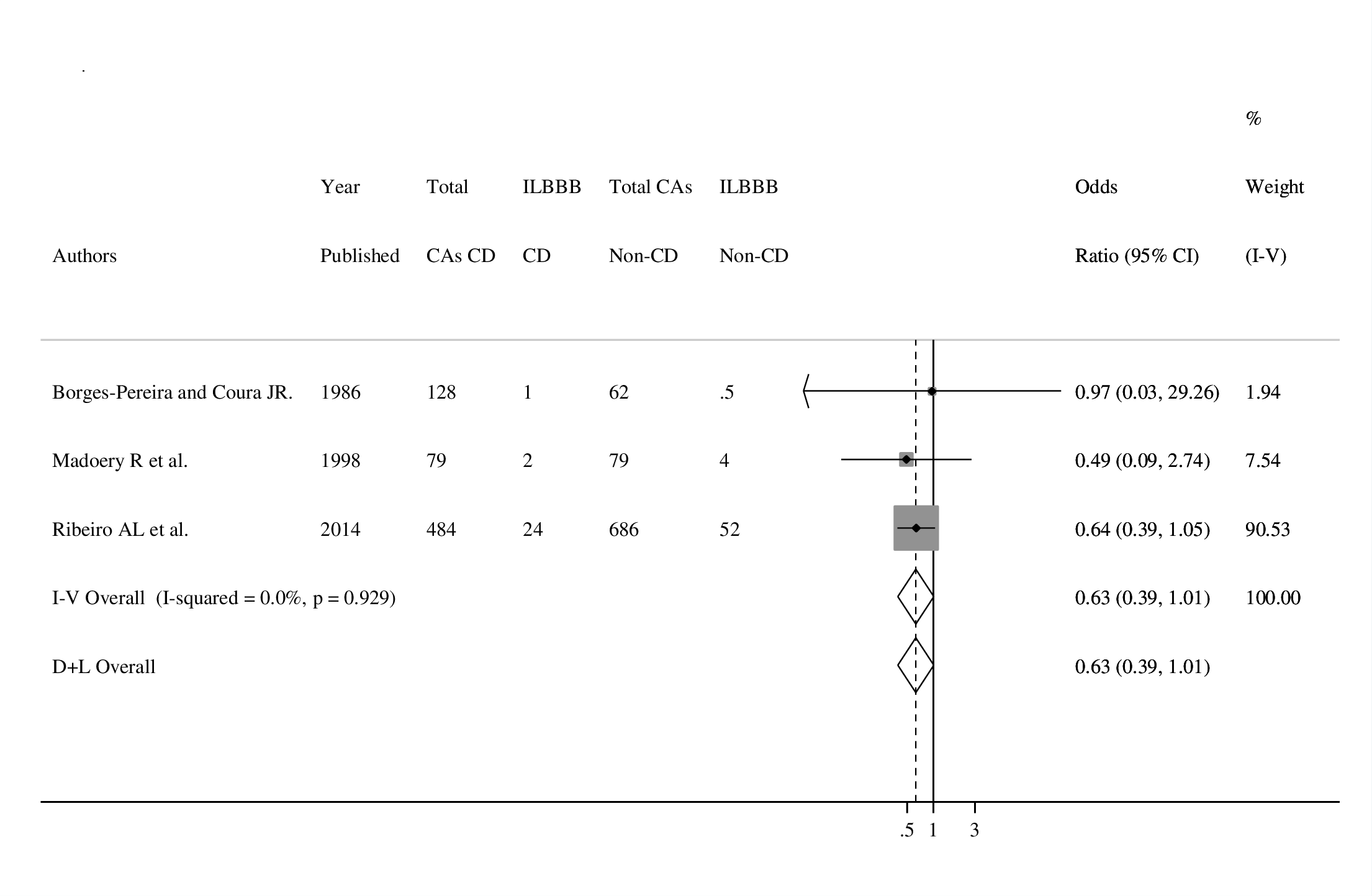

Supplement: S5 Fig — (TIF) [file pntd.0006567.s017.tif]

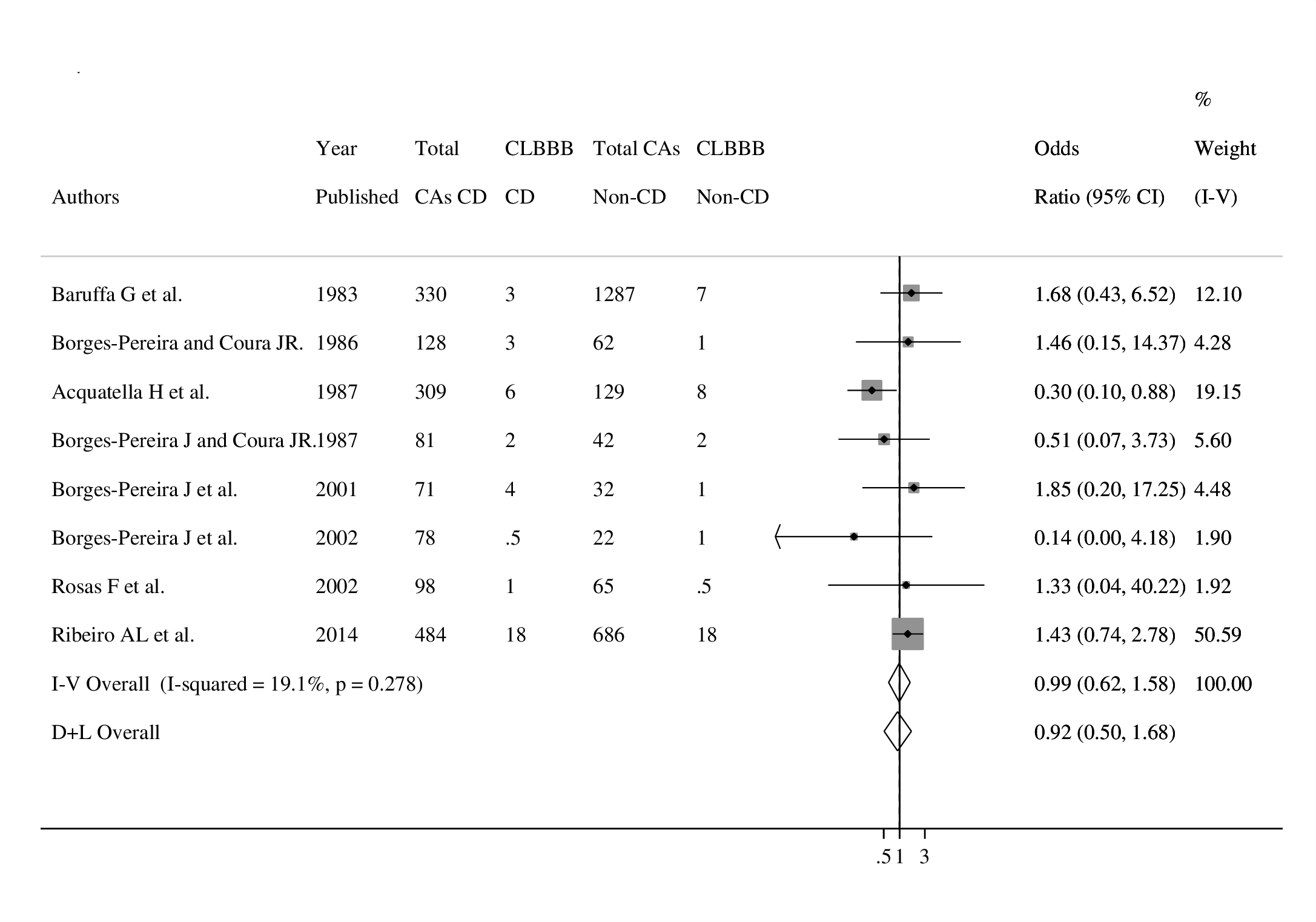

Supplement: S6 Fig — (TIF) [file pntd.0006567.s018.tif]

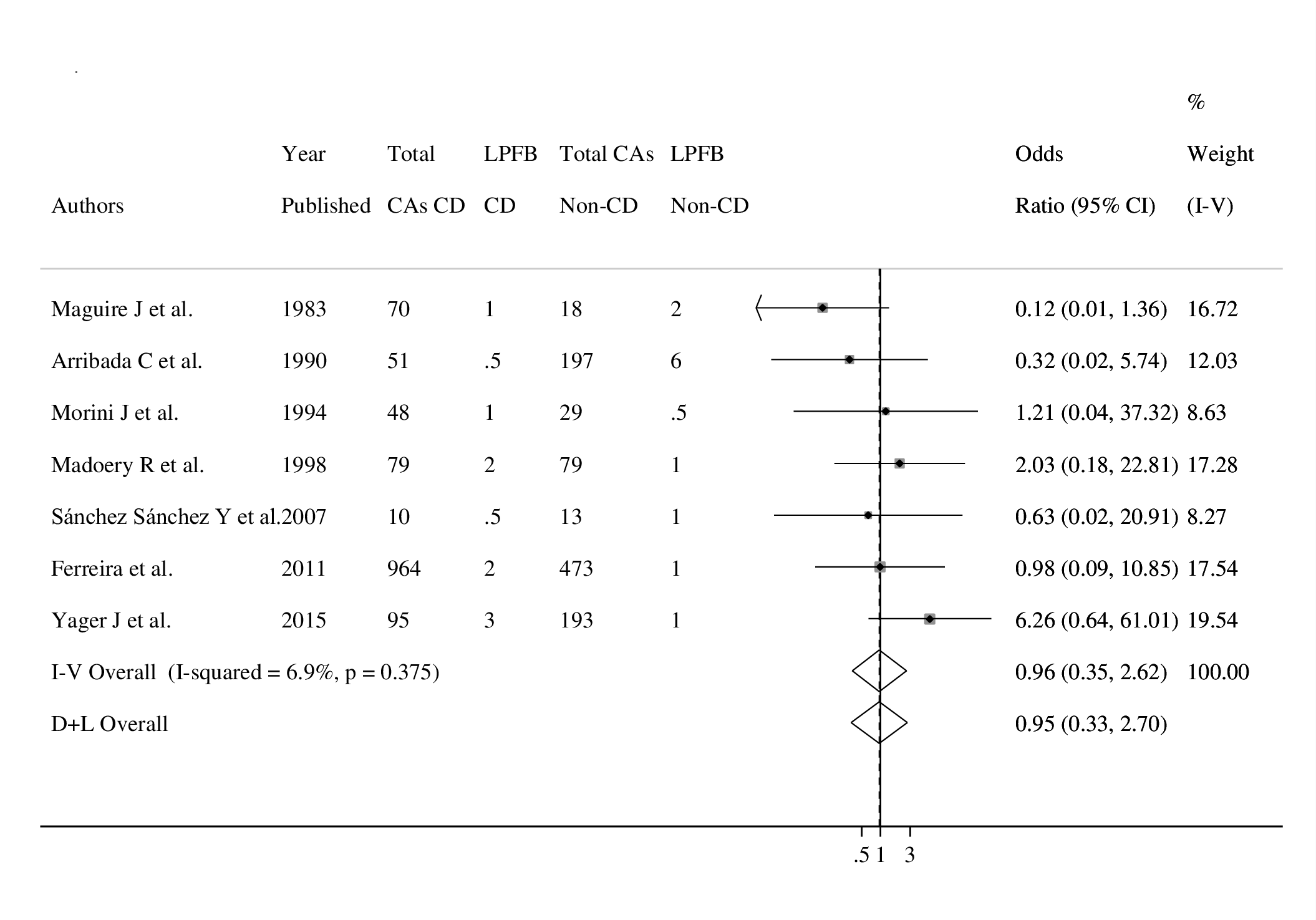

Supplement: S7 Fig — (TIF) [file pntd.0006567.s019.tif]

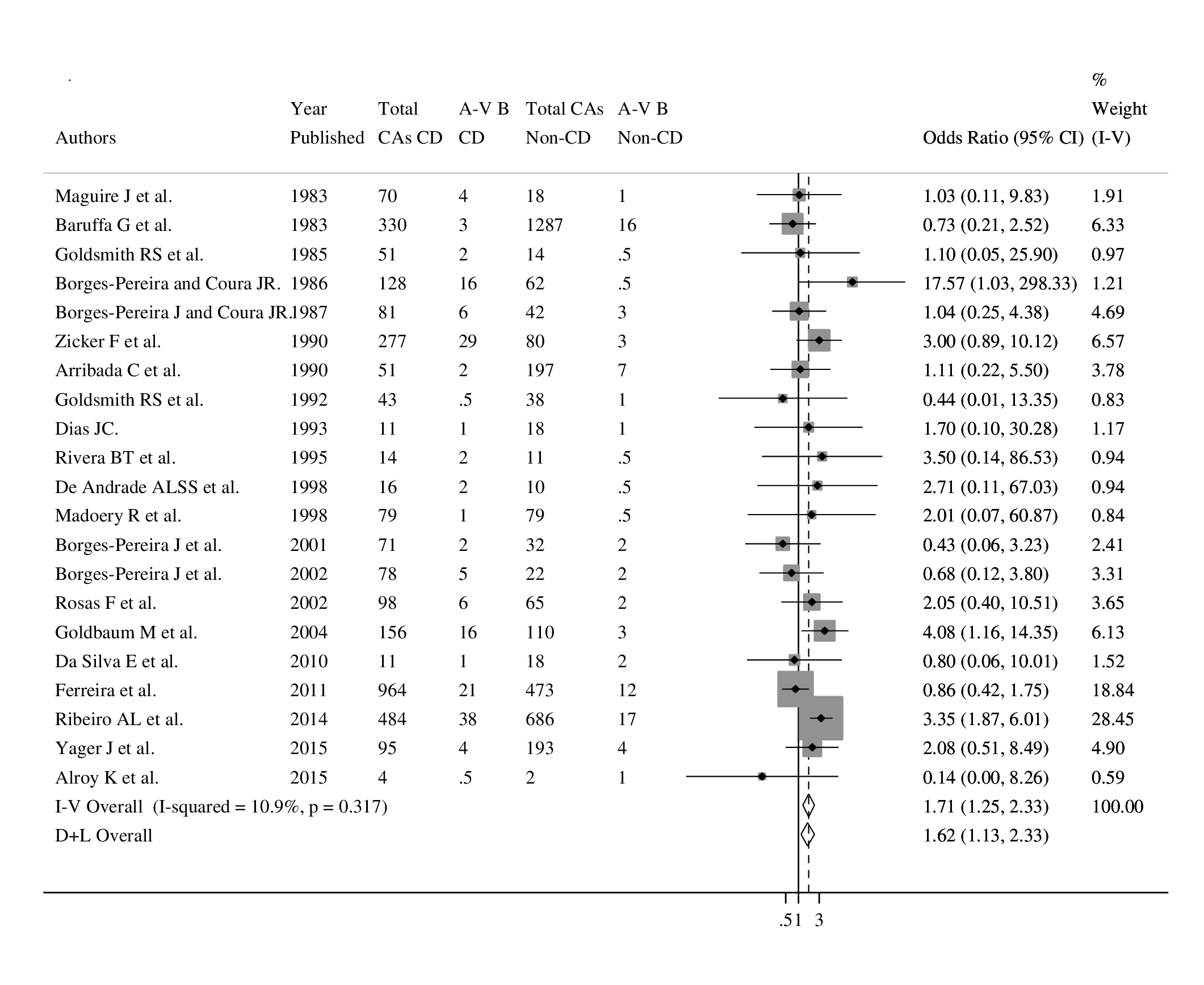

Supplement: S8 Fig — (TIF) [file pntd.0006567.s020.tif]

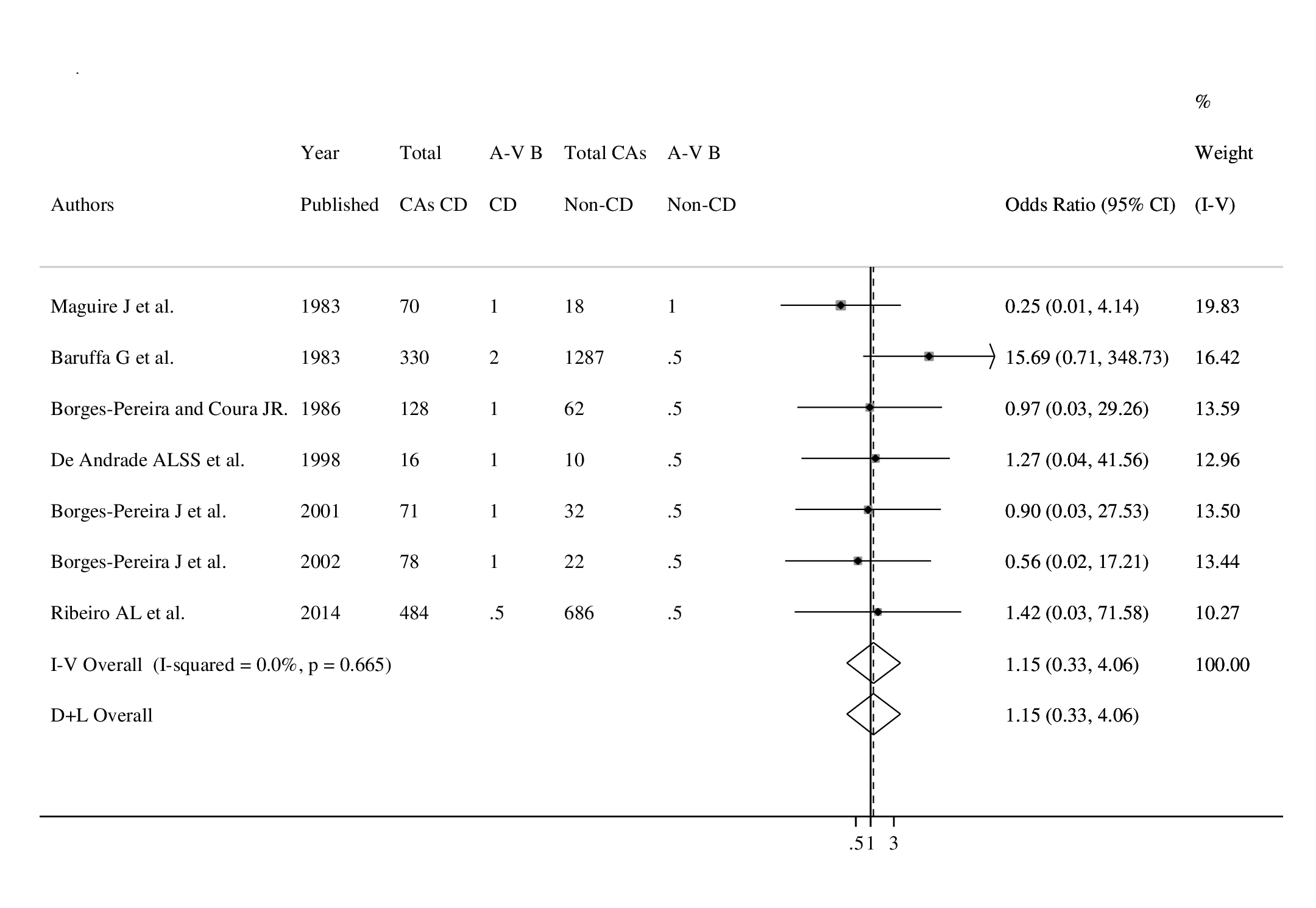

Supplement: S9 Fig — (TIF) [file pntd.0006567.s021.tif]

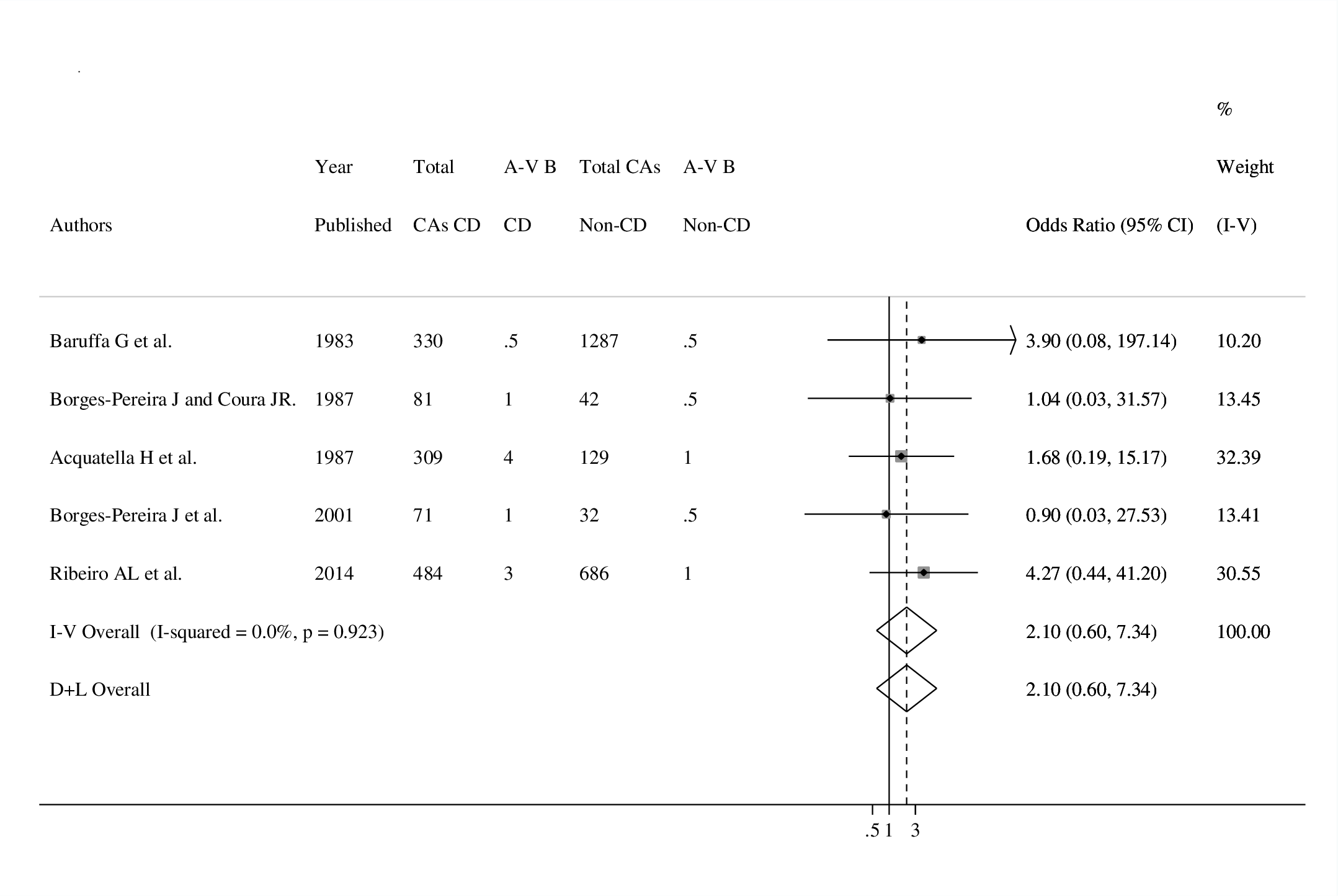

Supplement: S10 Fig — (TIF) [file pntd.0006567.s022.tif]

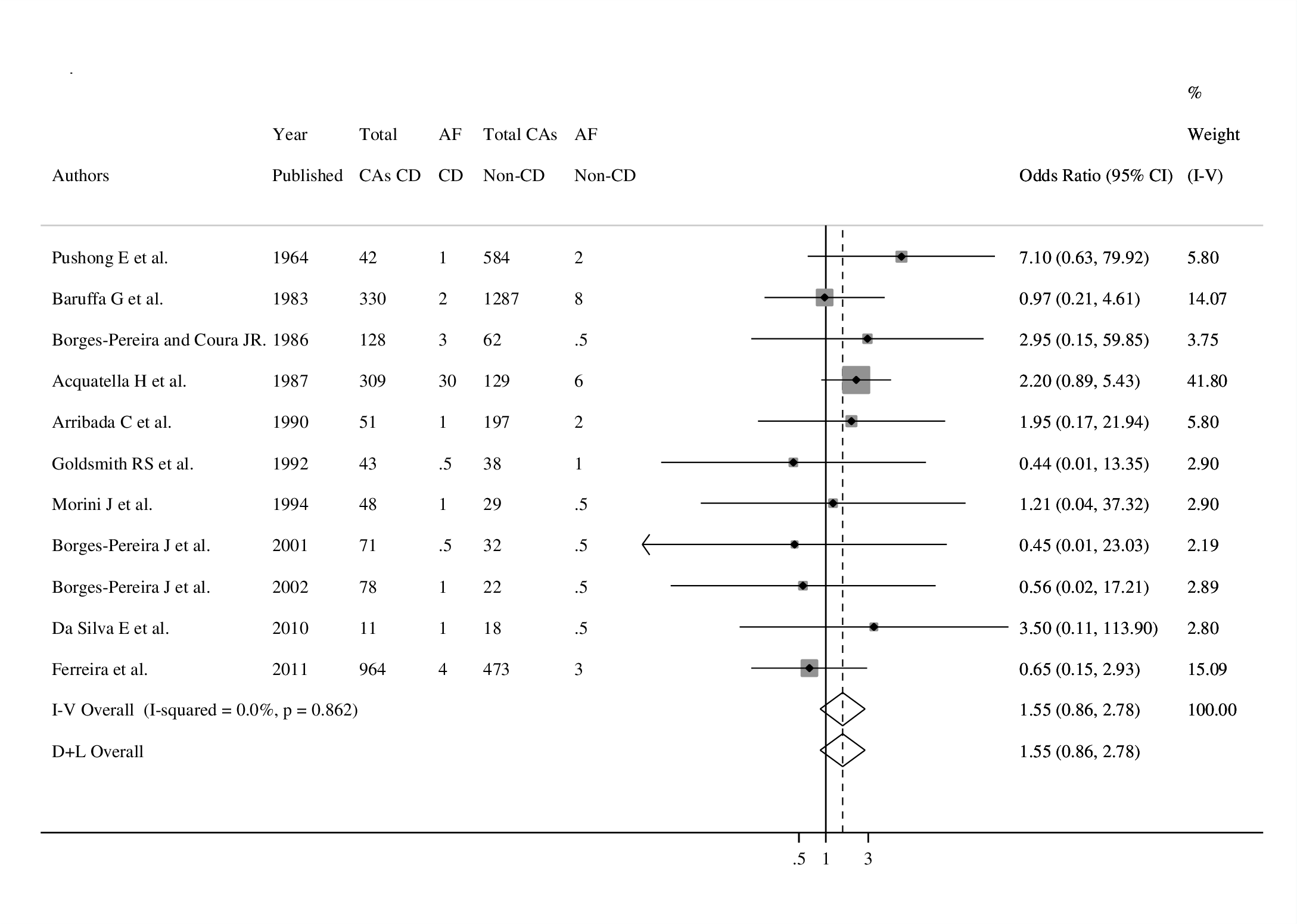

Supplement: S11 Fig — (TIF) [file pntd.0006567.s023.tif]

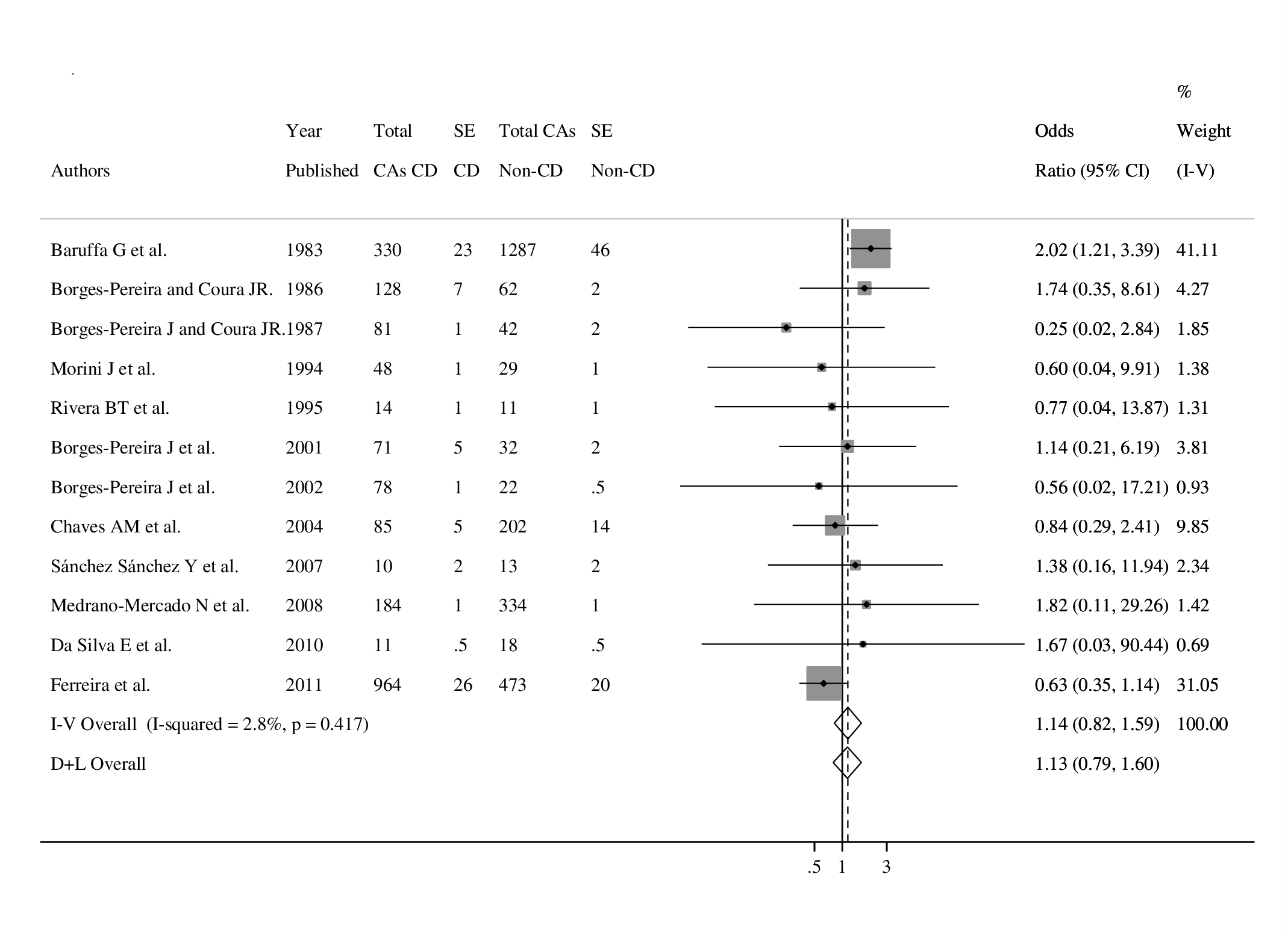

Supplement: S12 Fig — (TIF) [file pntd.0006567.s024.tif]

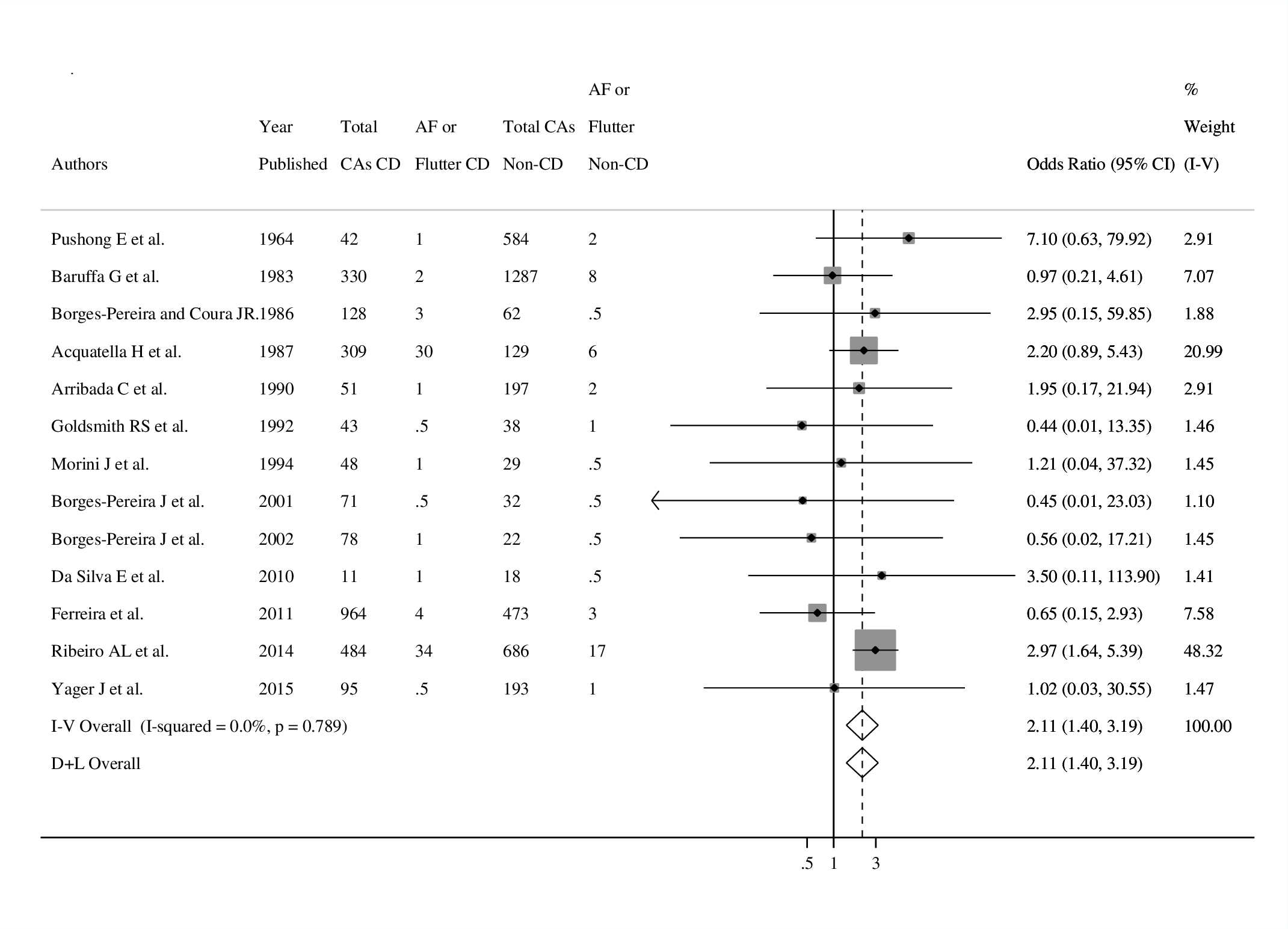

Supplement: S13 Fig — (TIF) [file pntd.0006567.s025.tif]

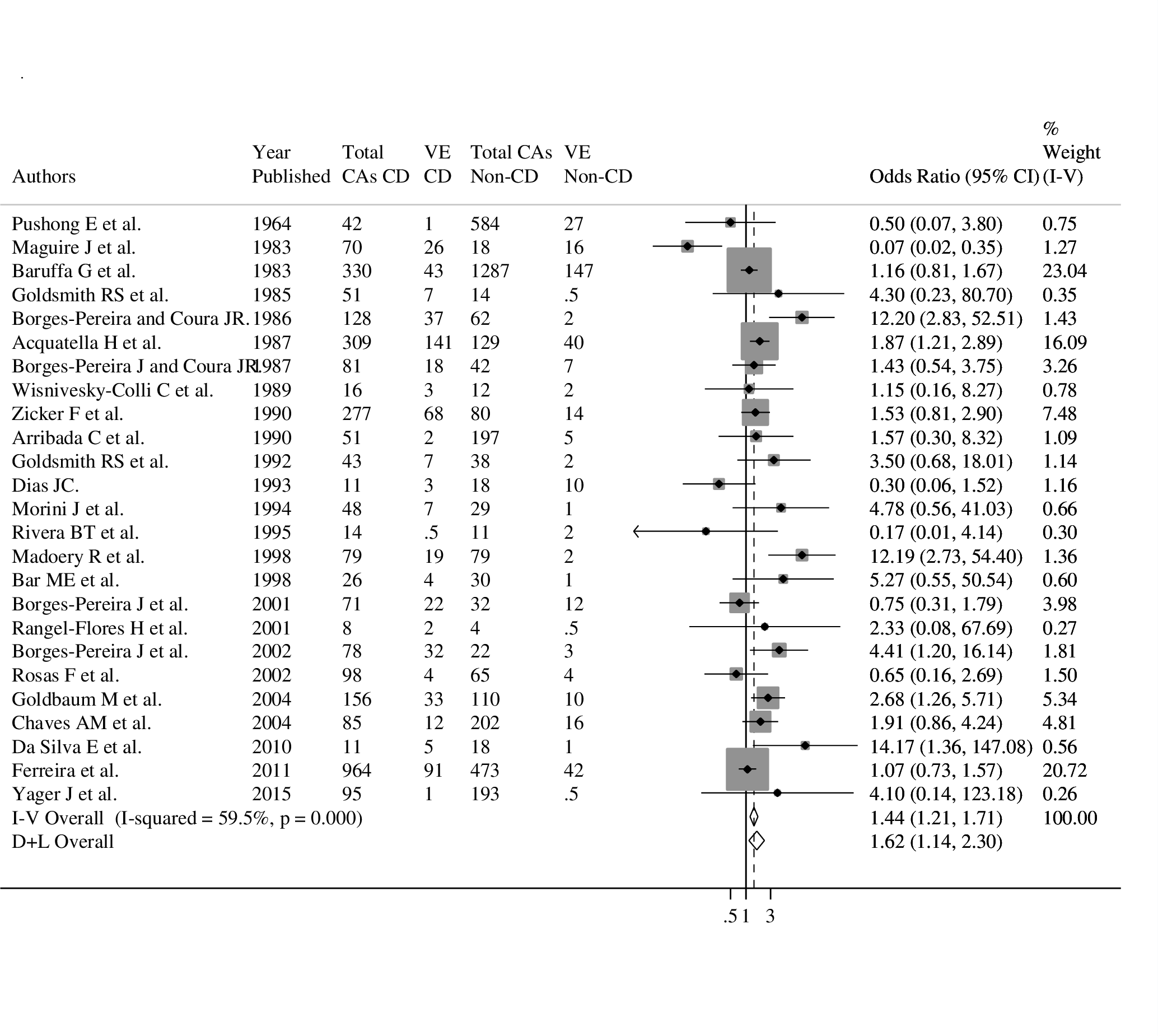

Supplement: S14 Fig — (TIF) [file pntd.0006567.s026.tif]

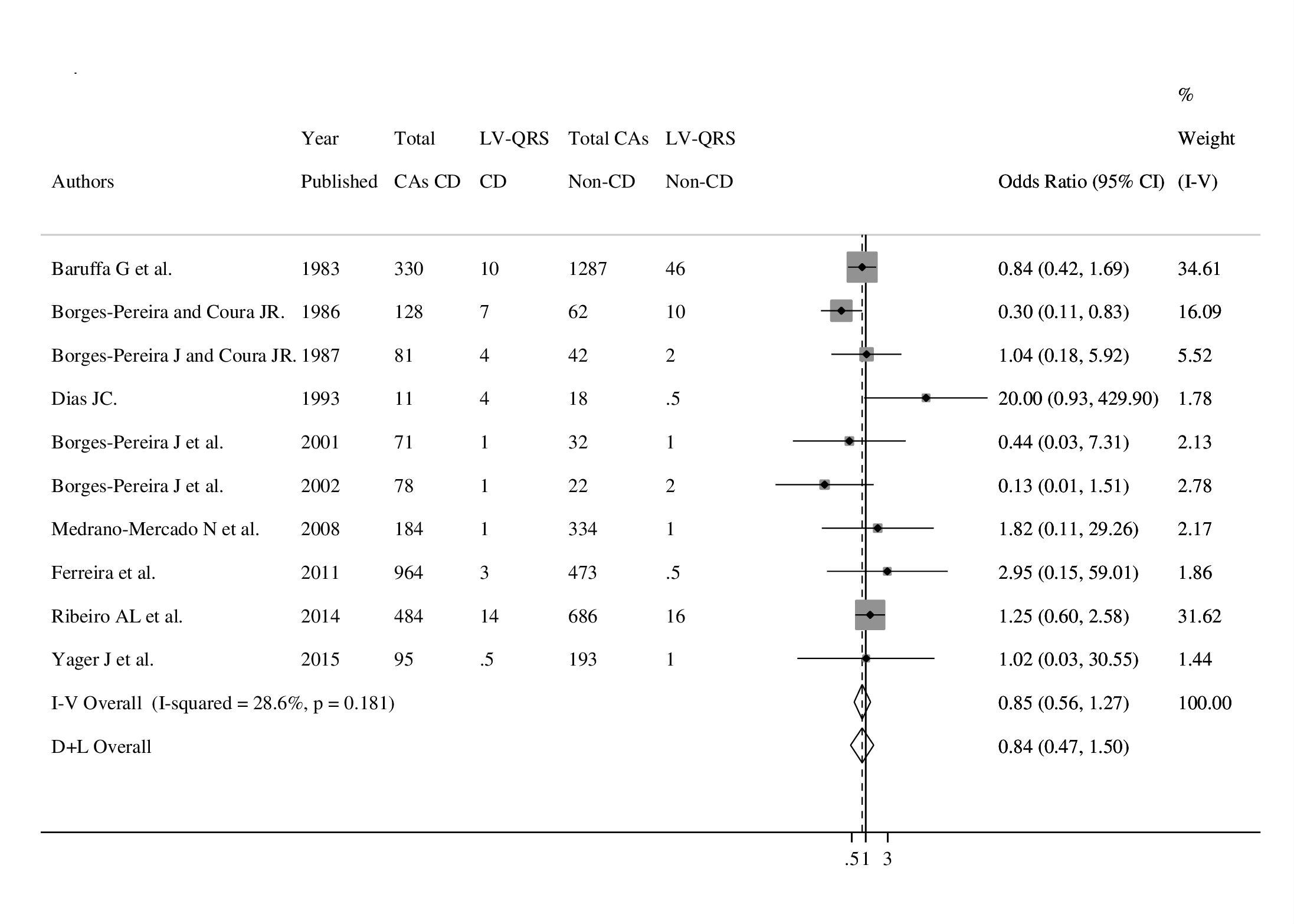

Supplement: S15 Fig — (TIF) [file pntd.0006567.s027.tif]

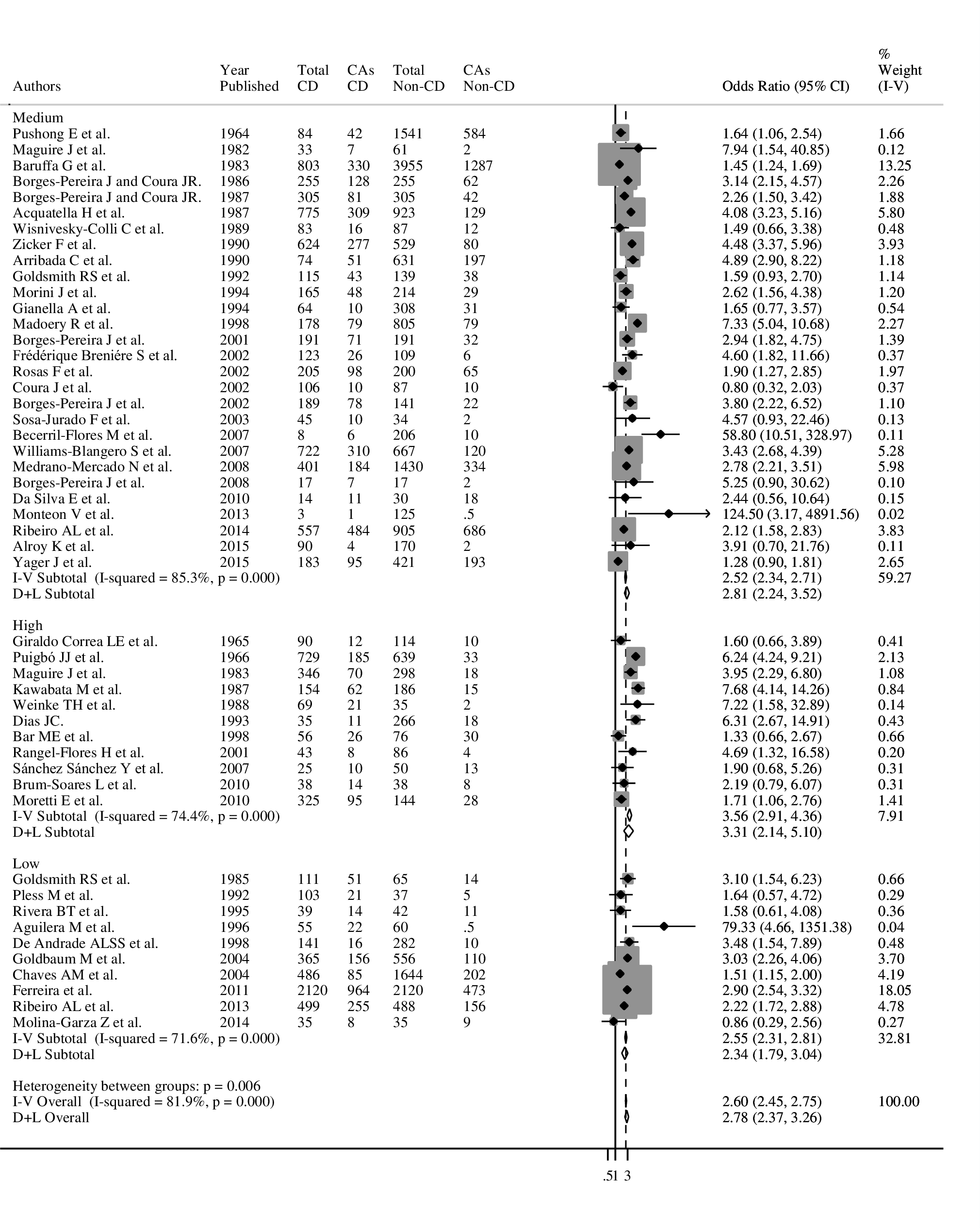

Supplement: S16 Fig — (TIF) [file pntd.0006567.s028.tif]

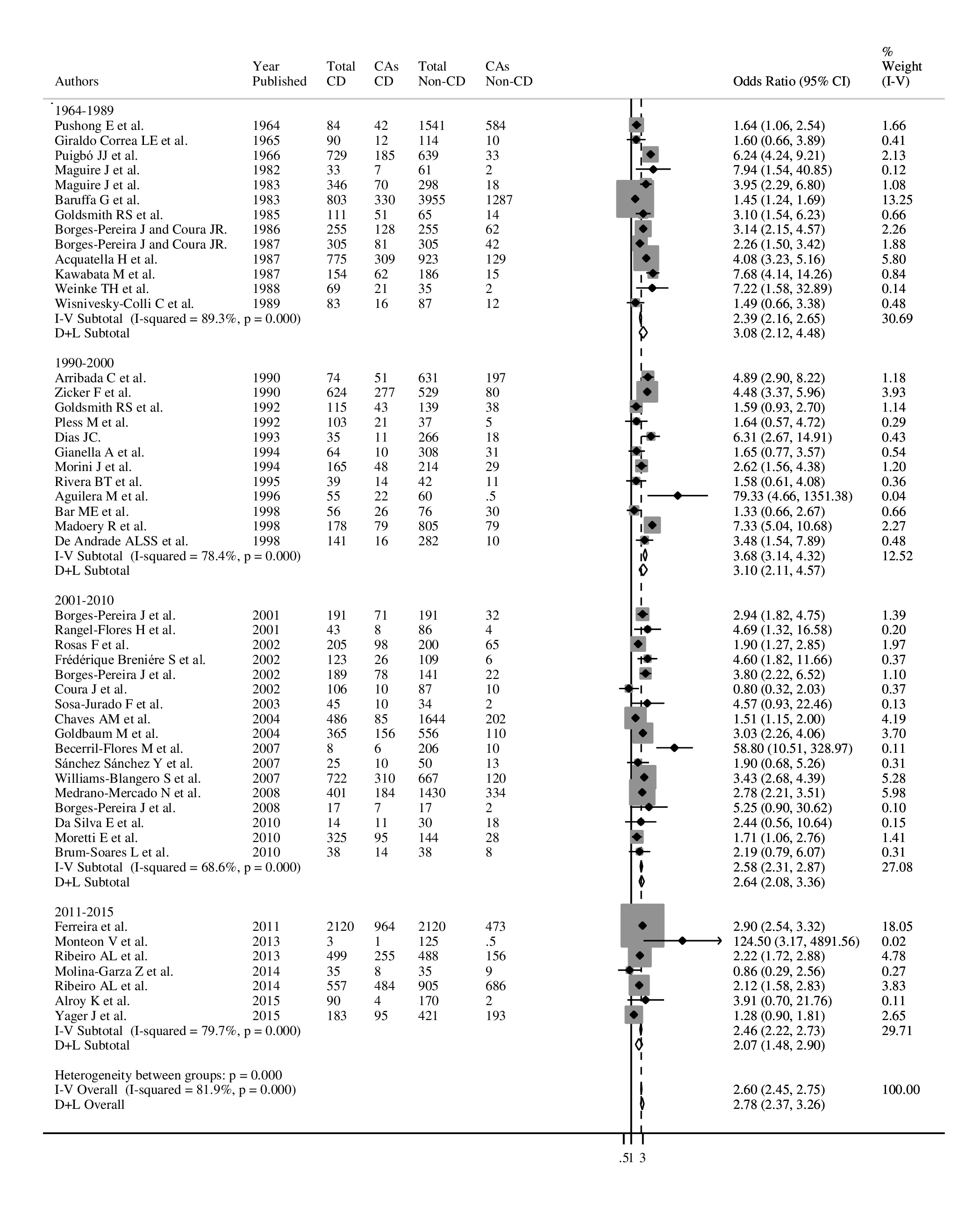

Supplement: S17 Fig — (TIF) [file pntd.0006567.s029.tif]

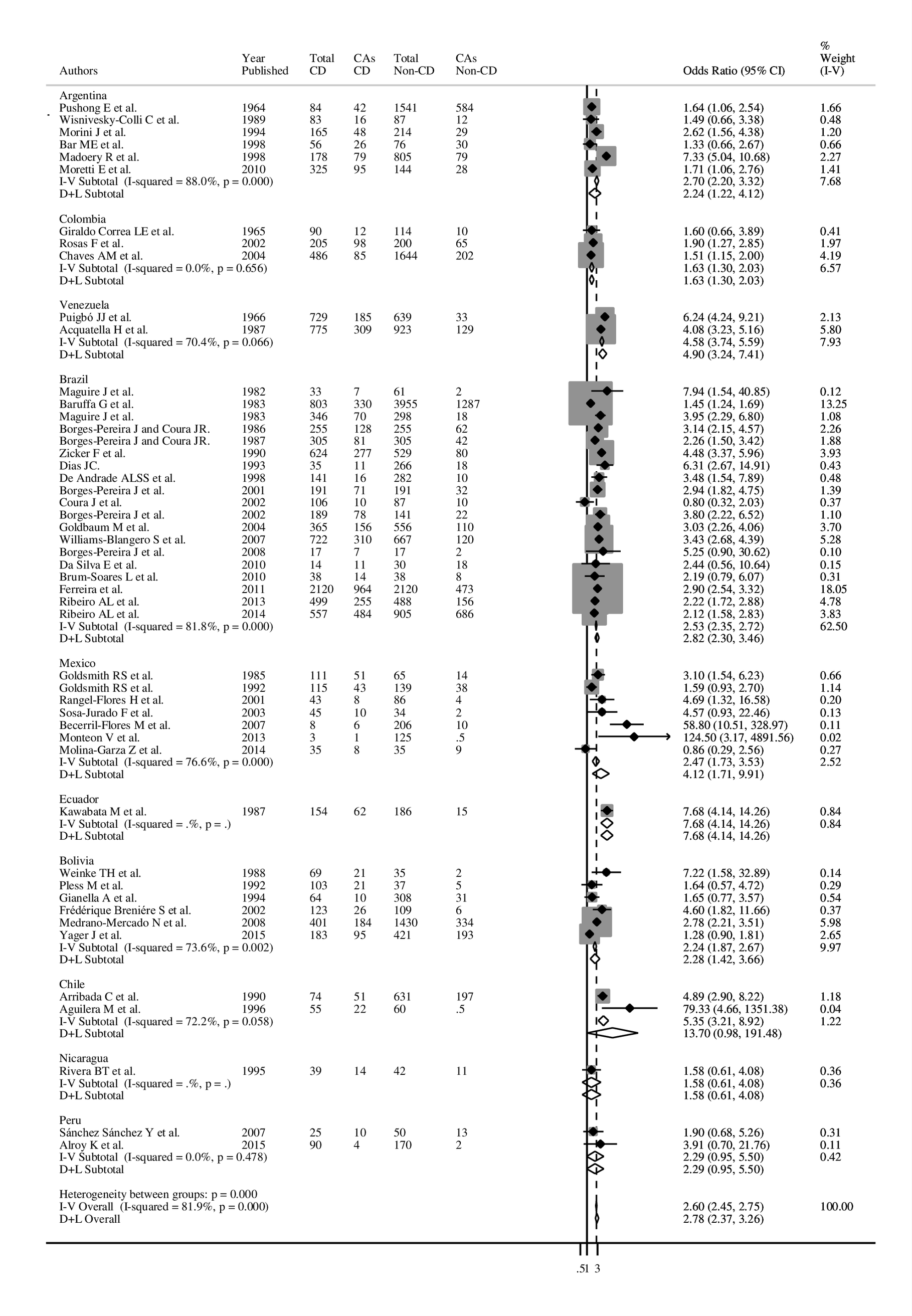

Supplement: S18 Fig — (TIF) [file pntd.0006567.s030.tif]

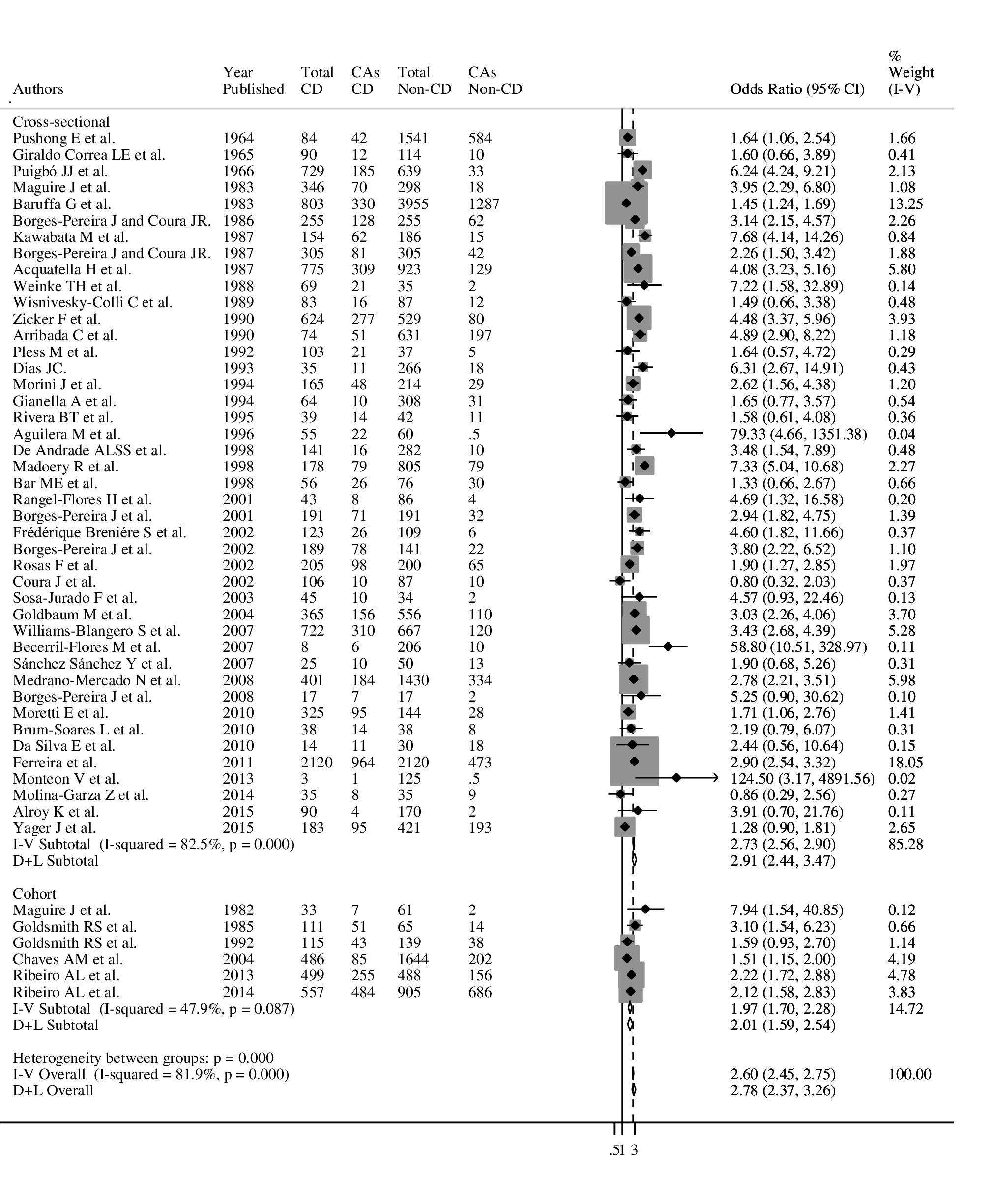

Supplement: S19 Fig — (TIF) [file pntd.0006567.s031.tif]

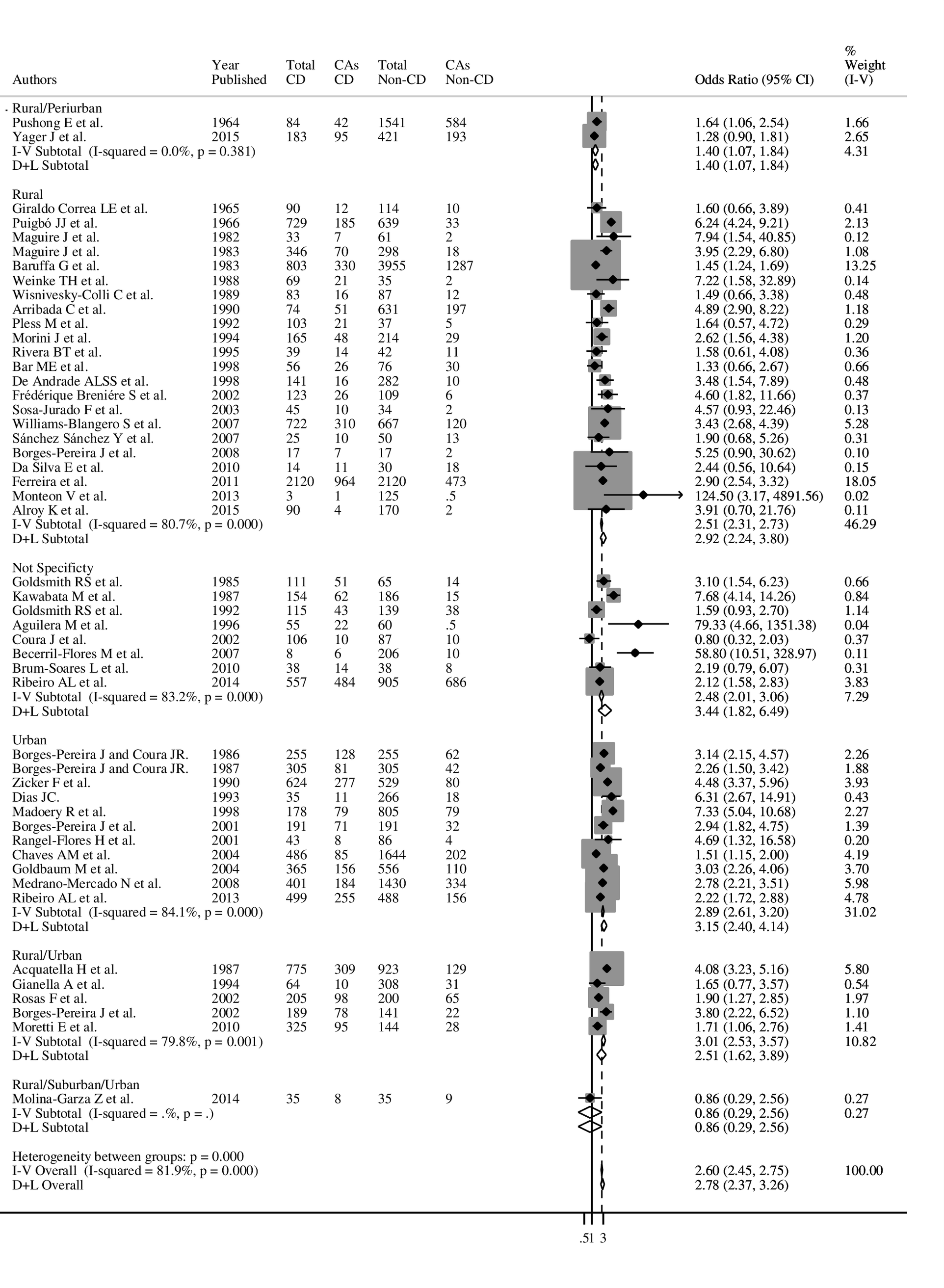

Supplement: S20 Fig — (TIF) [file pntd.0006567.s032.tif]

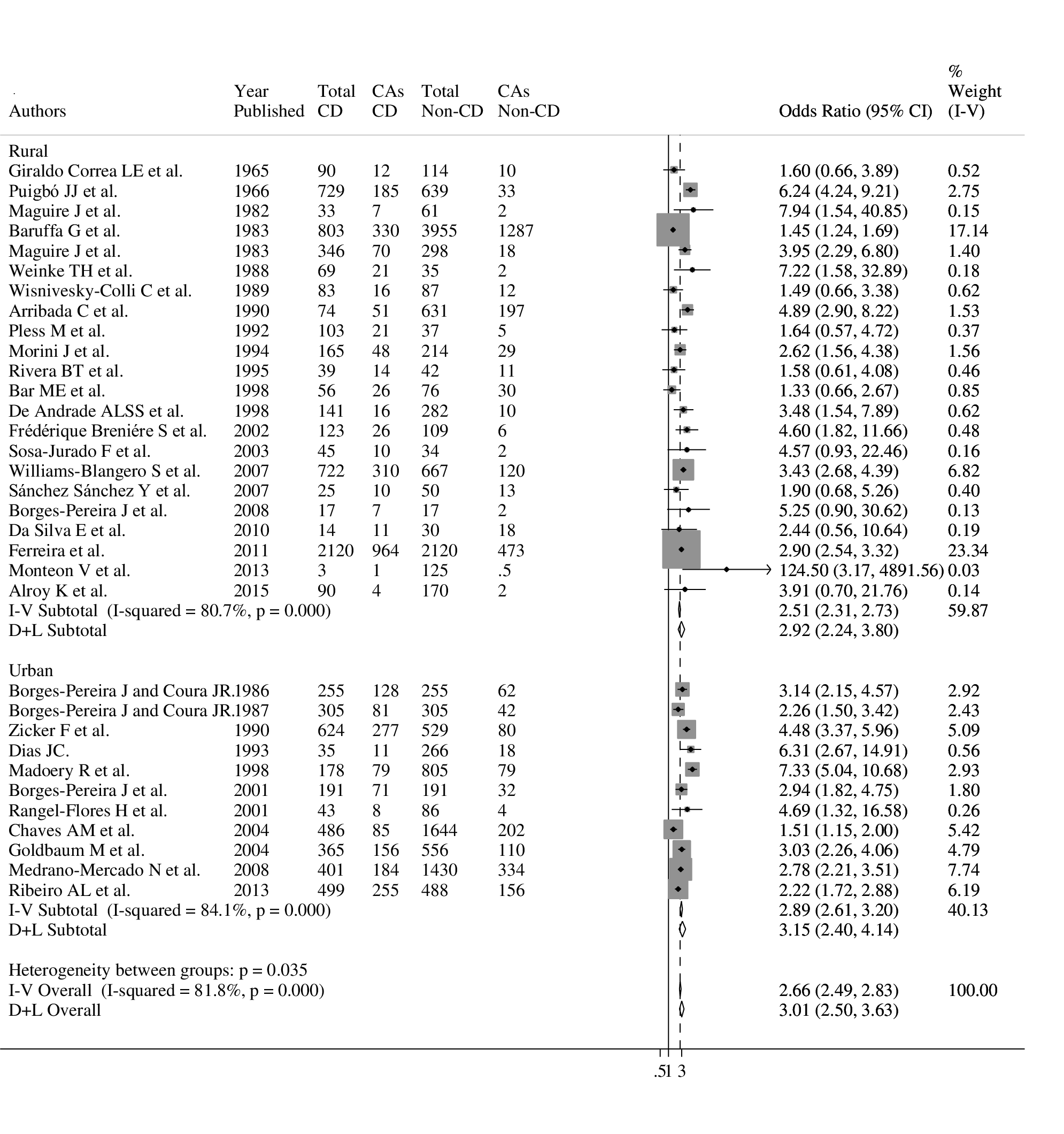

Supplement: S21 Fig — (TIF) [file pntd.0006567.s033.tif]

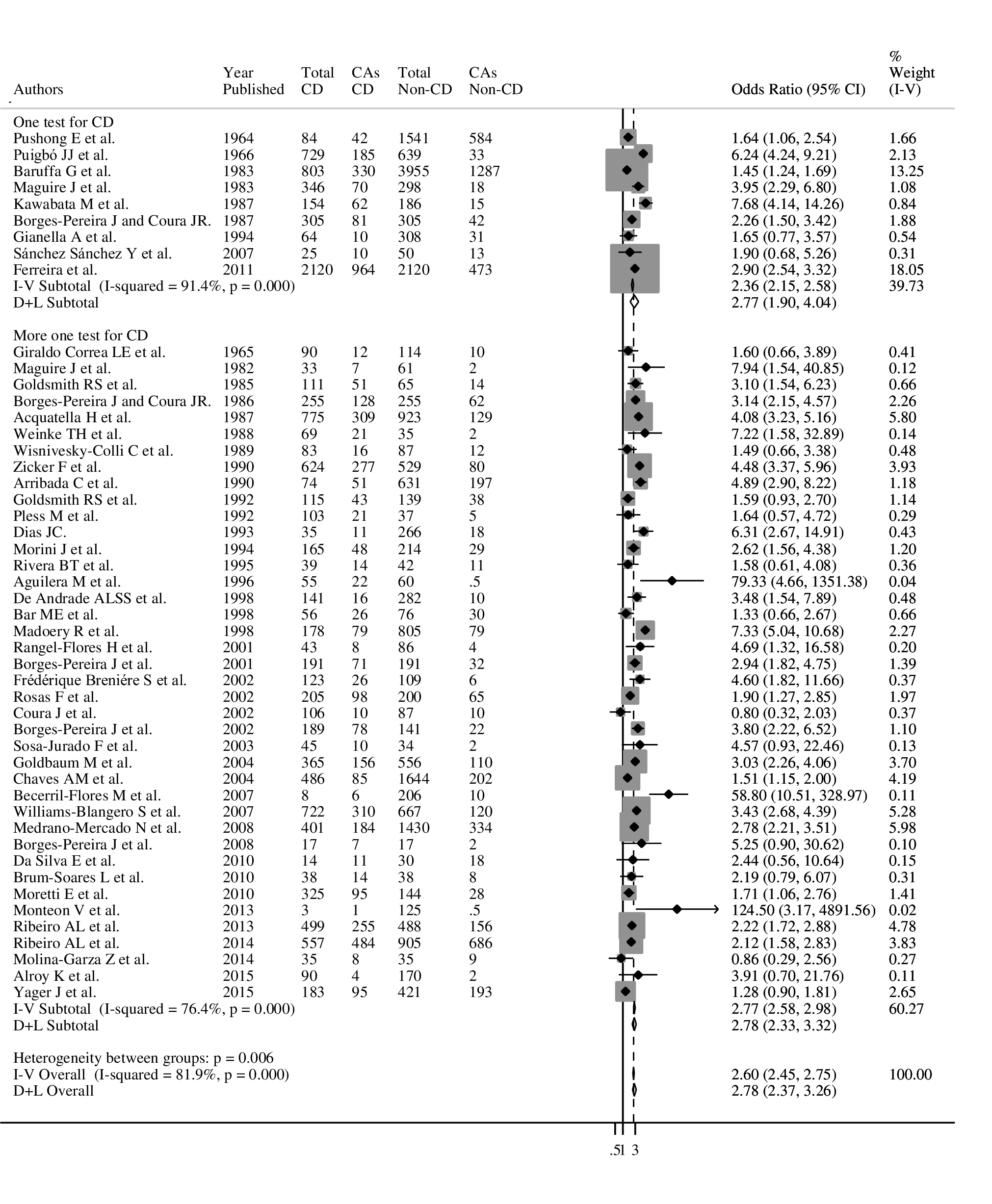

Supplement: S22 Fig — (TIF) [file pntd.0006567.s034.tif]

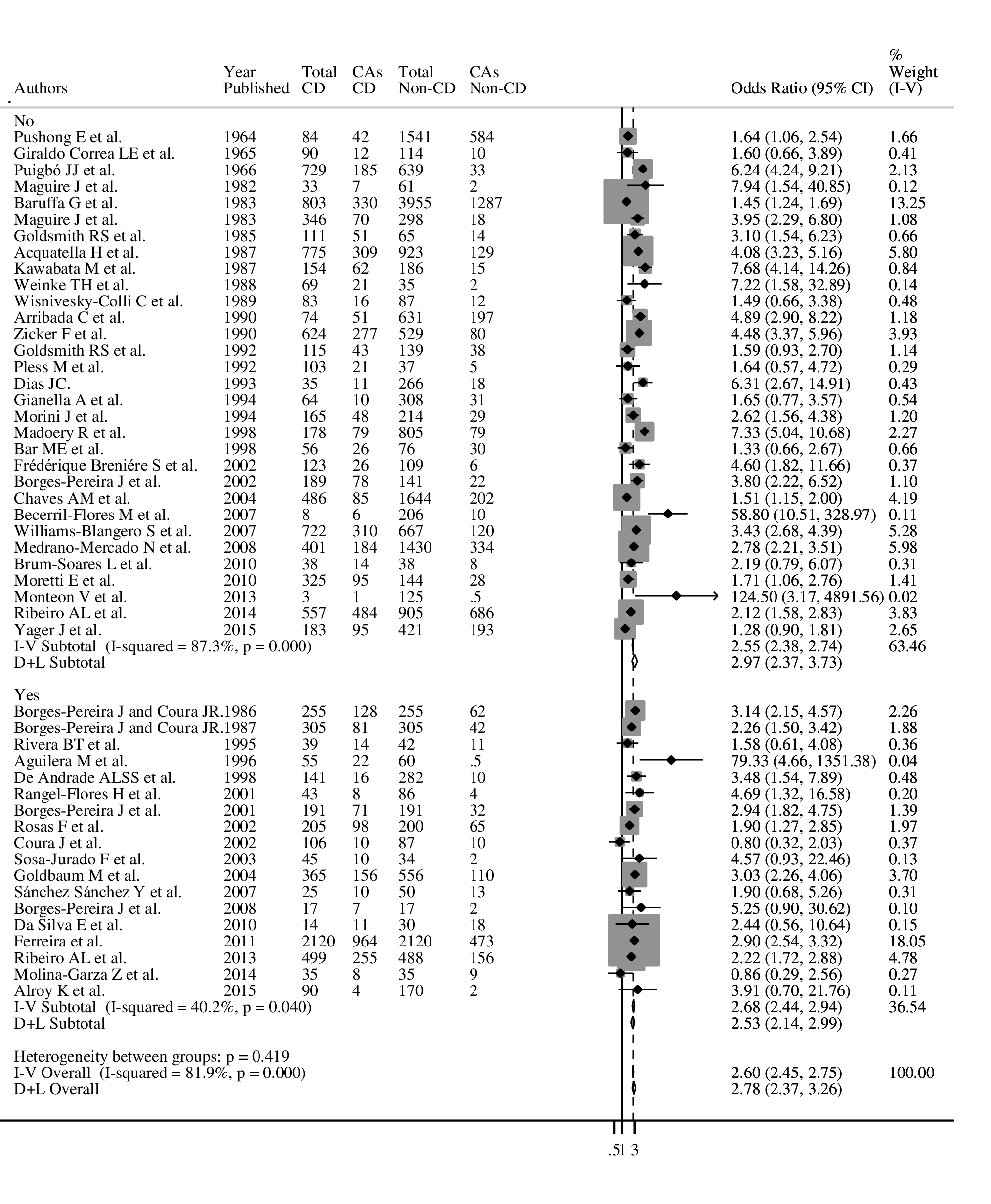

Supplement: S23 Fig — (TIF) [file pntd.0006567.s035.tif]

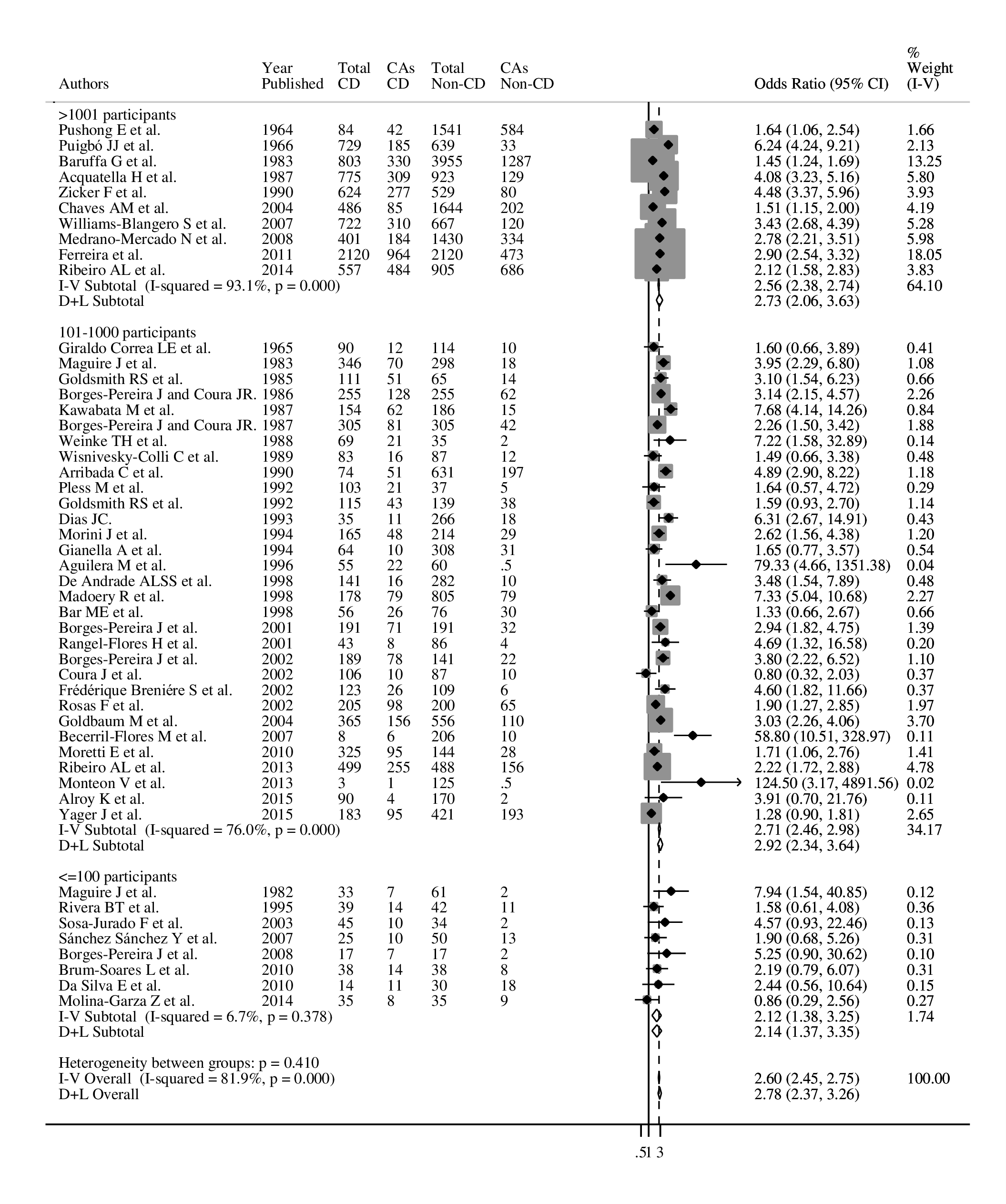

Supplement: S24 Fig — (TIF) [file pntd.0006567.s036.tif]

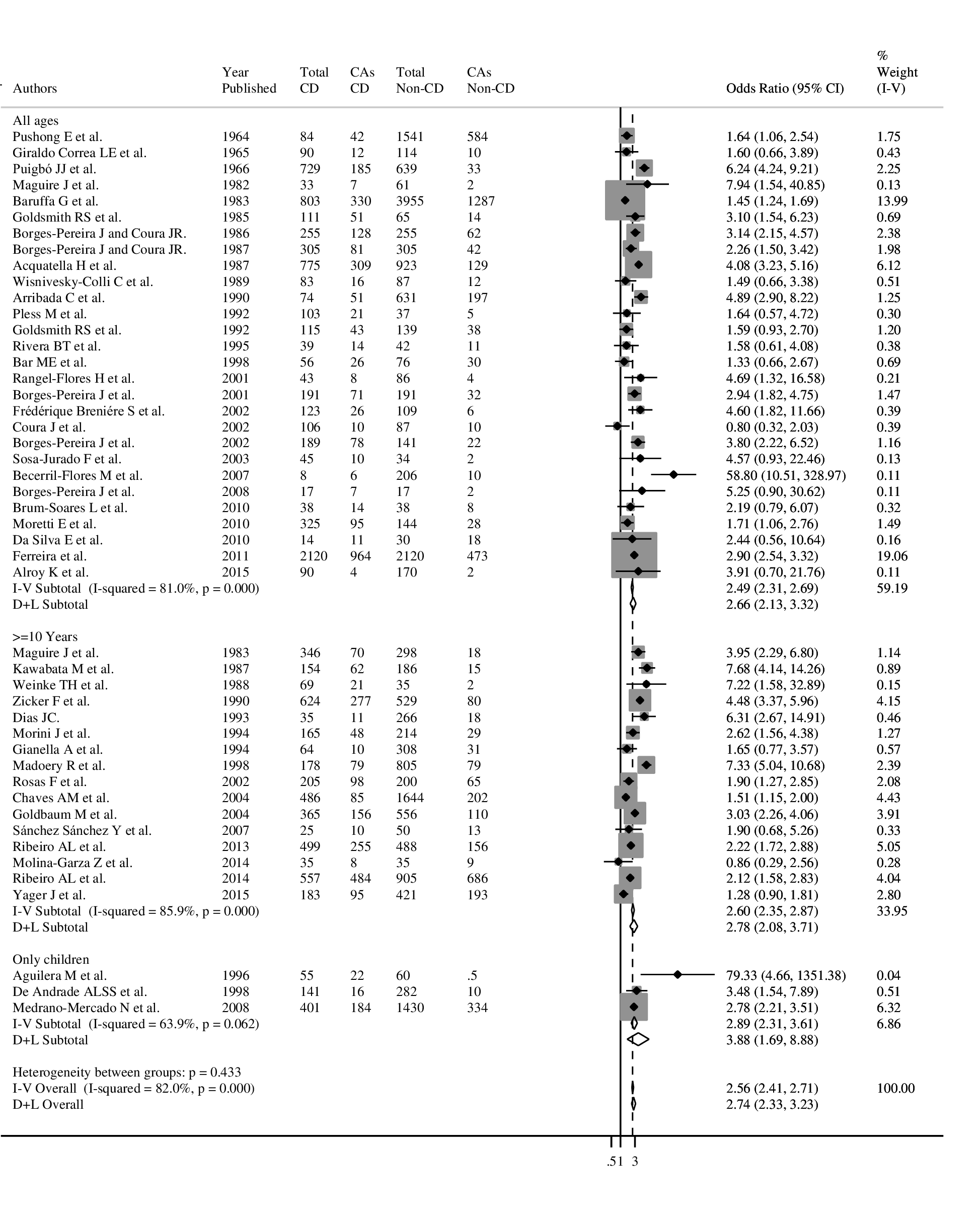

Supplement: S25 Fig — (TIF) [file pntd.0006567.s037.tif]

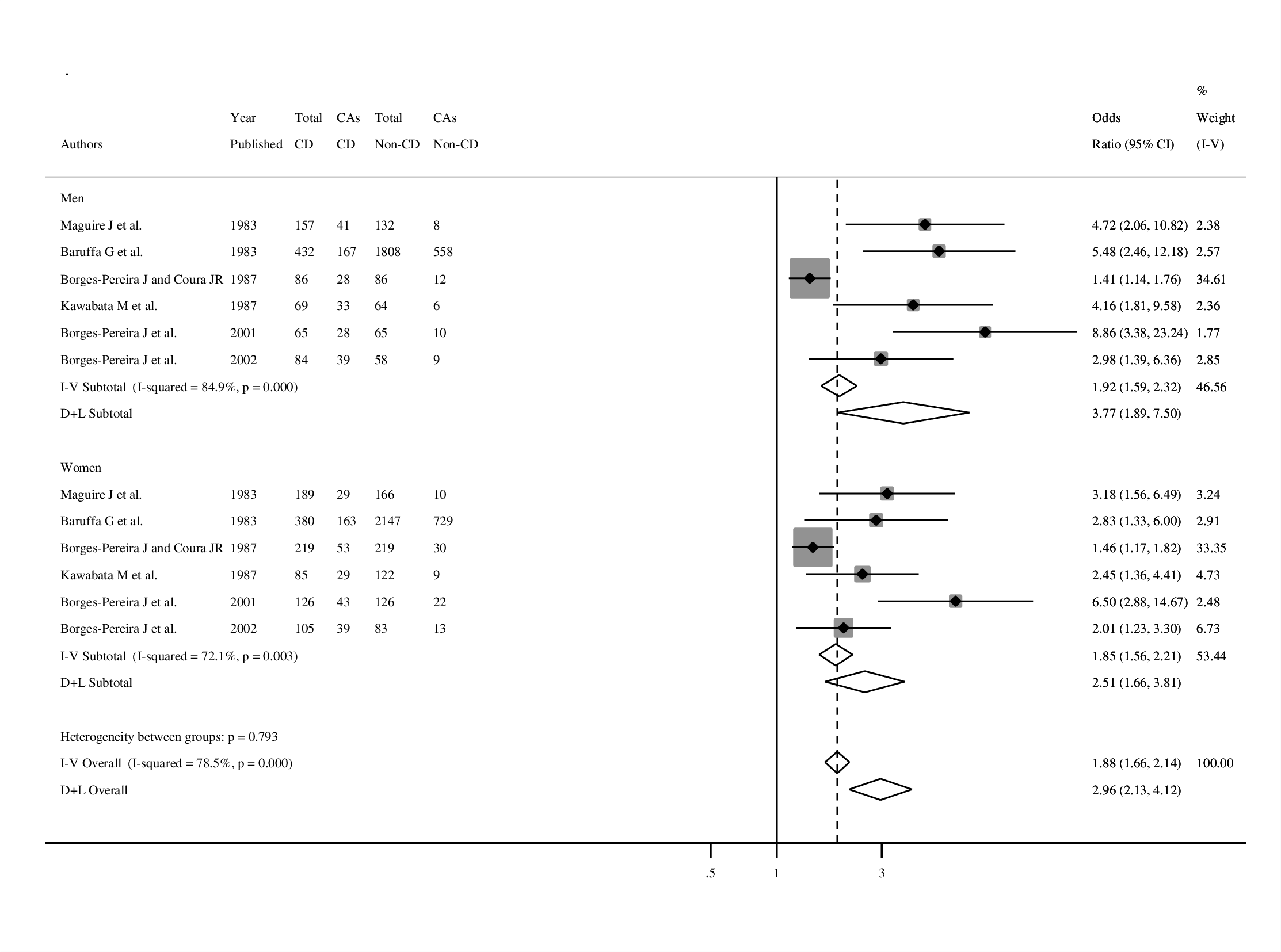

Supplement: S26 Fig — (TIF) [file pntd.0006567.s038.tif]

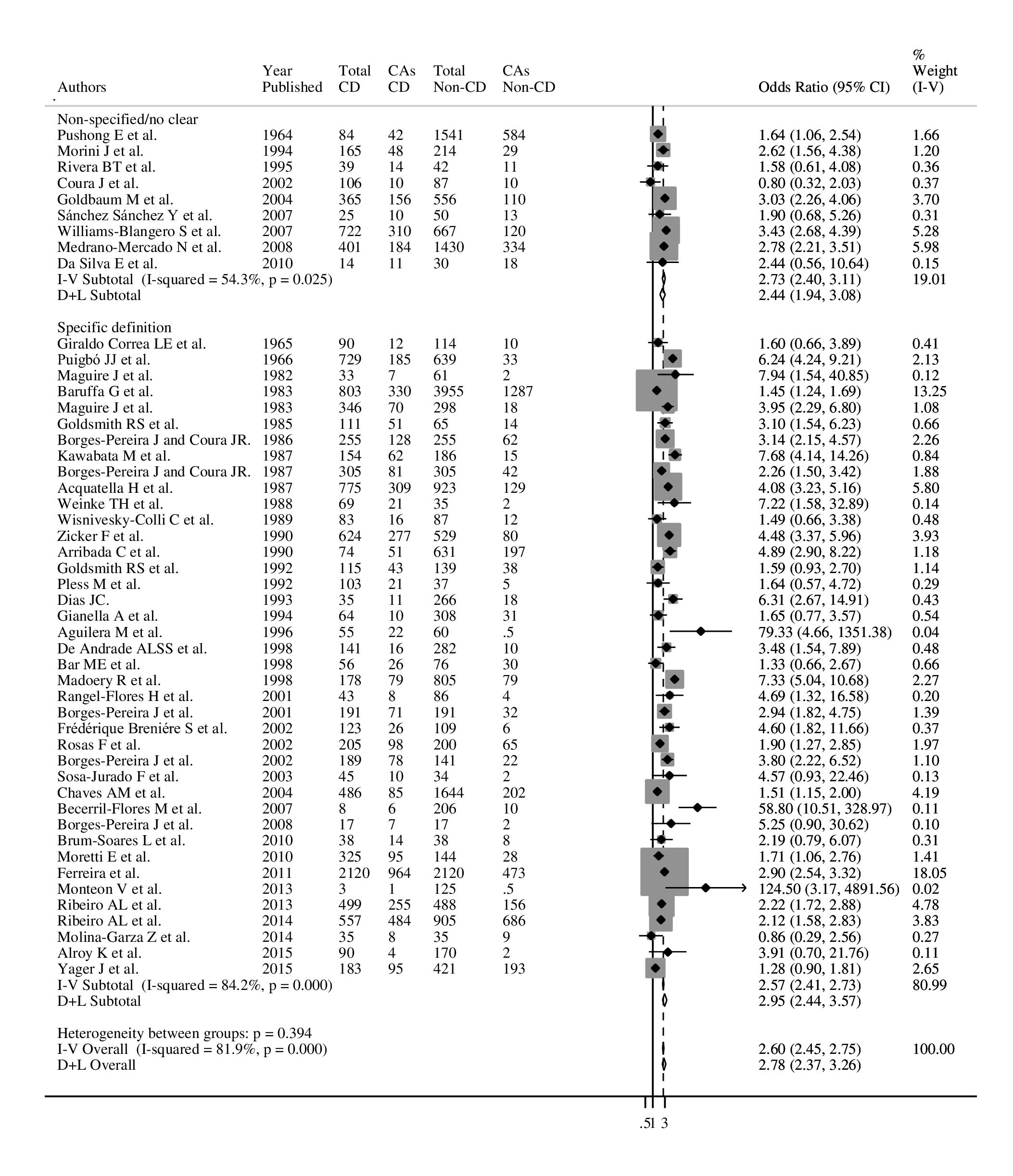

Supplement: S27 Fig — (TIF) [file pntd.0006567.s039.tif]

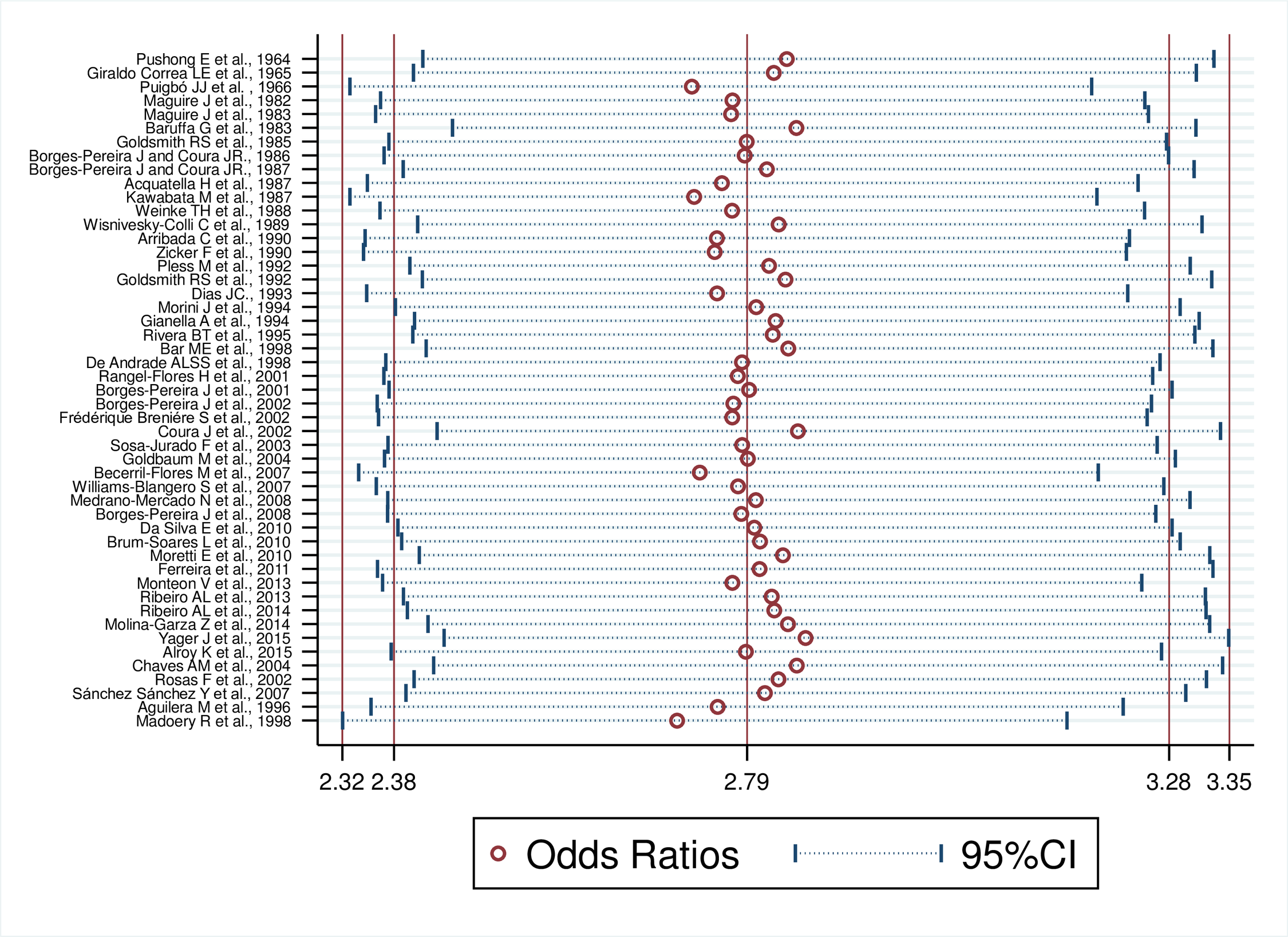

Supplement: S28 Fig — (TIF) [file pntd.0006567.s040.tif]

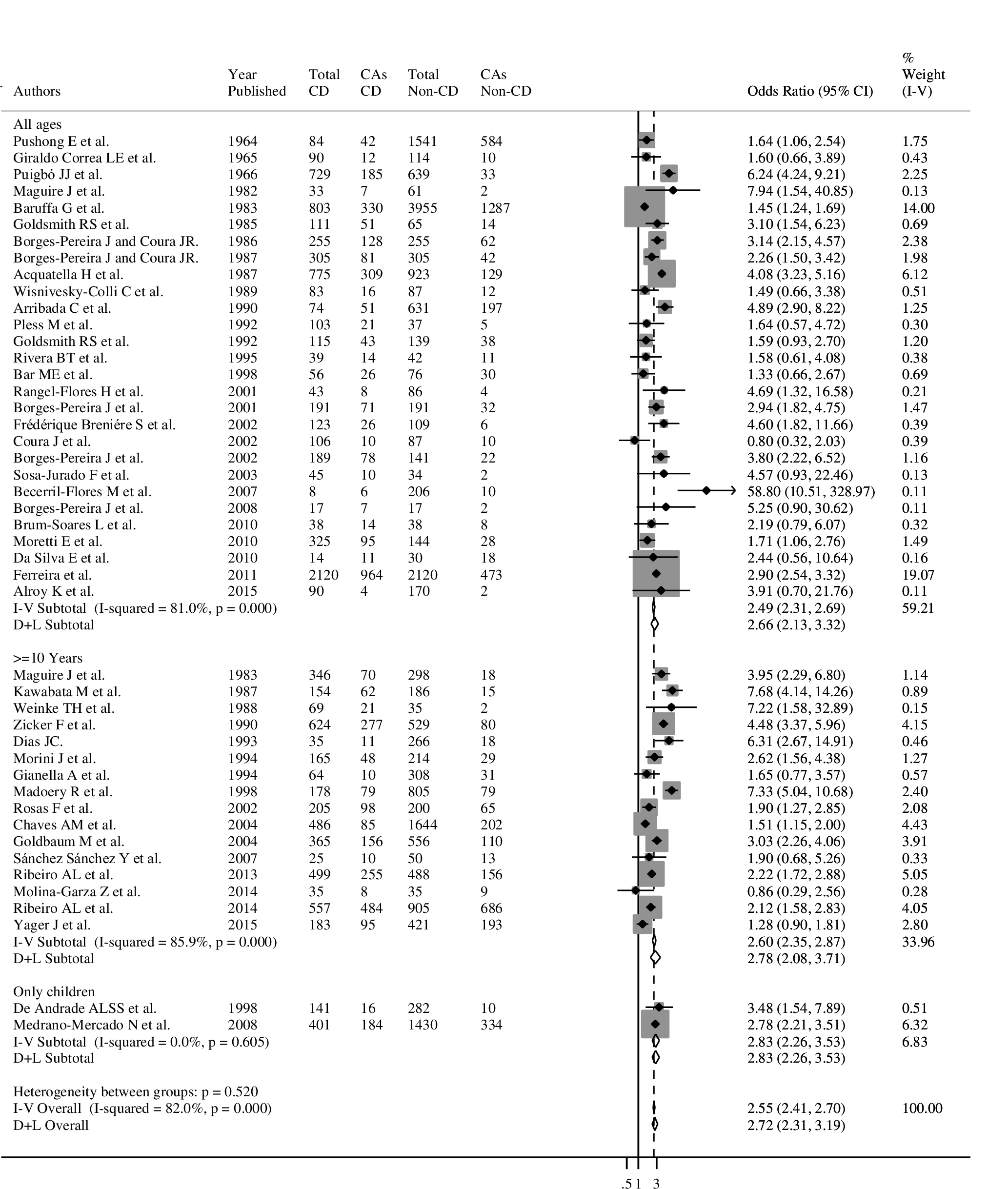

Supplement: S29 Fig — (TIF) [file pntd.0006567.s041.tif]
