# Supplementary material for: Electrocardiographic abnormalities in Chagas disease in the general population: A systematic review and meta-analysis
Source: PLoS Negl Trop Dis. 2018 Jun 13;12(6):e0006567. doi: 10.1371/journal.pntd.0006567 (PMC5999094; doi:10.1371/journal.pntd.0006567)
Supplement: S1 Diagram — (DOC) [file pntd.0006567.s043.doc]

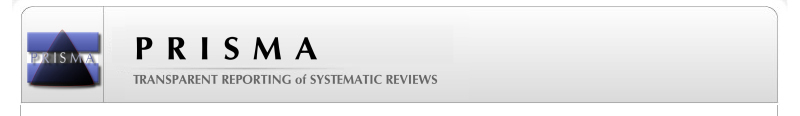
 **PRISMA 2009 Flow Diagram**

**Screening**

**Included**

**Eligibility**

**Identification**

Records identified through database searching
(n=8,799)

Additional records identified through other sources
(n=11)

Records after duplicates removed
(n=5,396)

Records screened
(n=5,396)

Records excluded
(n=5,144)

Full-text articles assessed for eligibility
(n=252)

Full-text articles excluded, with reasons
(n=203)

119 Population not relevant

40 Studies design not relevant

31 Relevant outcome not reported or not clarified

10 Preliminary results or sub-studies

3 Overlapping period and setting of study

Studies included in qualitative synthesis
(n=49)

Studies included in quantitative synthesis (meta-analysis)
(n=49)
