# Supplementary material for: Electrocardiographic abnormalities in Chagas disease in the general population: A systematic review and meta-analysis
Source: PLoS Negl Trop Dis. 2018 Jun 13;12(6):e0006567. doi: 10.1371/journal.pntd.0006567 (PMC5999094; doi:10.1371/journal.pntd.0006567)
Supplement: Supplementary file 15 [file pntd.0006567.s042.docx]

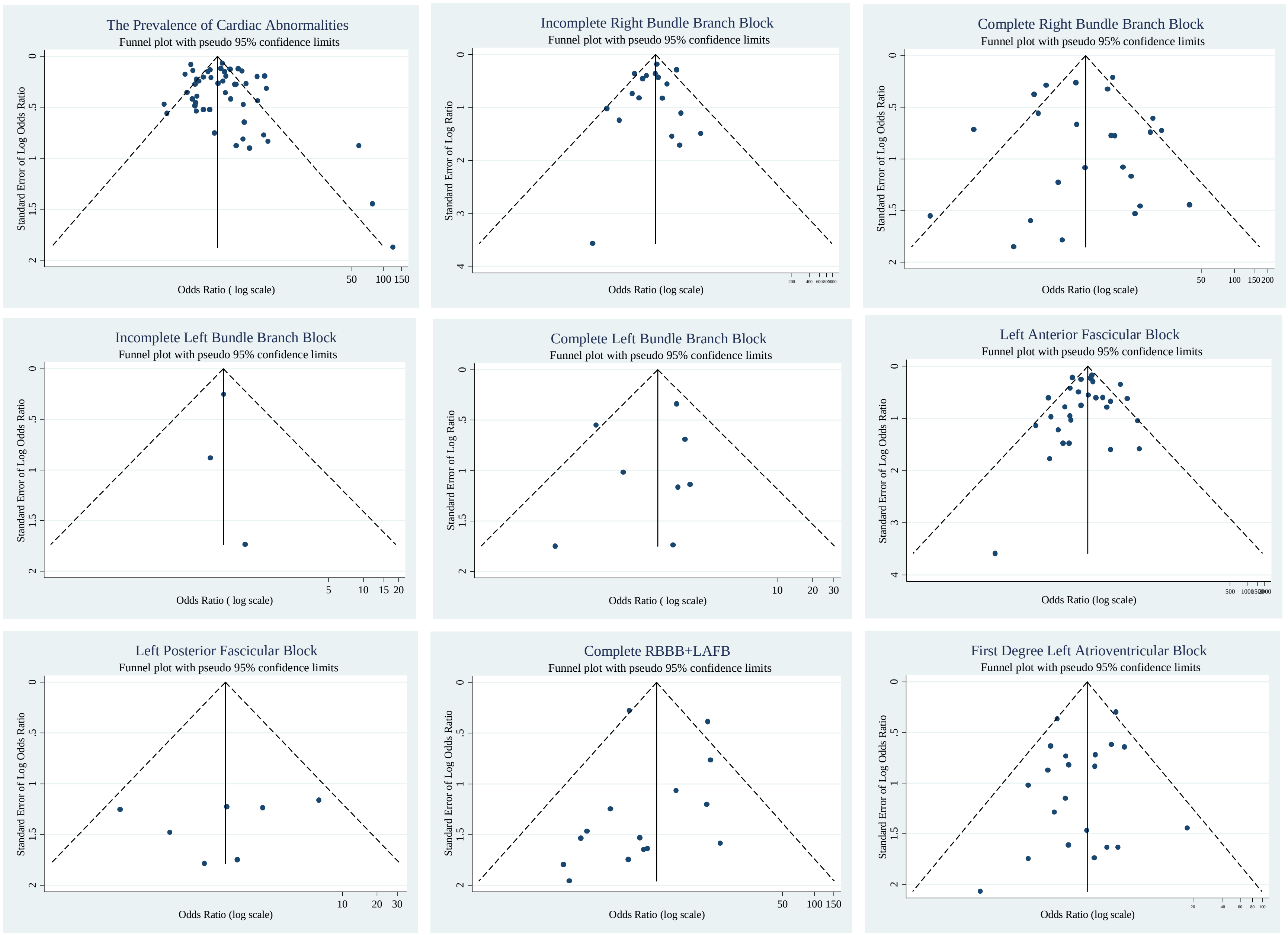
The Prevalence of Cardiac Abnormalities
Funnel plot with pseudo 95% confidence limits
Standard Error of Log Odds Ratio
0
.5
1
1.5
2
50
100
150
Odds Ratio (log scale)
Incomplete Right Bundle Branch Block
Funnel plot with pseudo 95% confidence limits
Standard Error of Log Ratio
0
1
2
3
4
Odds Ratio (log scale)
Complete Right Bundle Branch Block
Funnel plot with pseudo 95% confidence limits
Standard Error of Log Odds Ratio
0
.5
1
1.5
2
50
100
150
200
Odds Ratio (log scale)
Incomplete Left Bundle Branch Block
Funnel plot with pseudo 95% confidence limits
Standard Error of Log Odds Ratio
0
.5
1
1.5
2
5
10
15
20
Odds Ratio (log scale)
Complete Left Bundle Branch Block
Funnel plot with pseudo 95% confidence limits
Standard Error of Log Odds Ratio
0
.5
1
1.5
2
10
20
30
Odds Ratio (log scale)
Left Anterior Fascicular Block
Funnel plot with pseudo 95% confidence limits
Standard Error of Log Odds Ratio
0
1
2
3
4
500
Odds Ratio (log scale)
Left Posterior Fascicular Block
Funnel plot with pseudo 95% confidence limits
Standard Error of Log Odds Ratio
0
.5
1
1.5
2
10
20
30
Odds Ratio (log scale)
Complete RBBB+LAFB
Funnel plot with pseudo 95% confidence limits
Standard Error of Log Odds Ratio
0
.5
1
1.5
2
50
100
150
Odds Ratio (log scale)
First Degree Left Atrioventricular Block
Funnel plot with pseudo 95% confidence limits
Standard Error of Log Odds Ratio
0
.5
1
1.5
2
Odds Ratio (log scale)

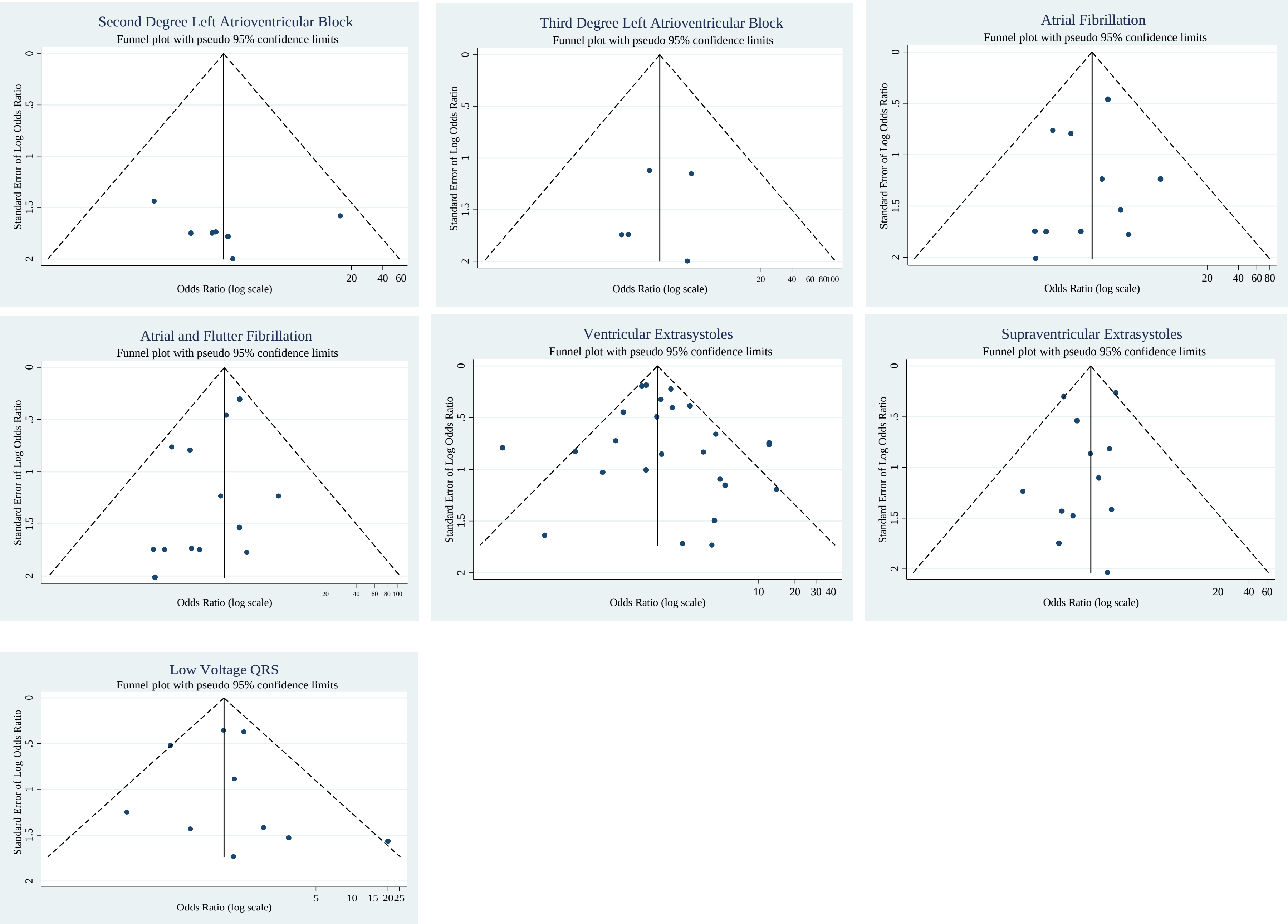
Second Degree Left Atrioventricular Block
Funnel plot with pseudo 95% confidence limits
Standard Error of Log Odds Ratio
0 .5 1 1.5 2
Odds Ratio (log scale)
20 40 60
Third Degree Left Atrioventricular Block
Funnel plot with pseudo 95% confidence limits
Standard Error of Log Odds Ratio
0 .5 1 1.5 2
Odds Ratio (log scale)
20 40 60 80100
Atrial Fibrillation
Funnel plot with pseudo 95% confidence limits
Standard Error of Log Odds Ratio
0 .5 1 1.5 2
Odds Ratio (log scale)
20 40 60 80
Atrial and Flutter Fibrillation
Funnel plot with pseudo 95% confidence limits
Standard Error of Log Odds Ratio
0 .5 1 1.5 2
Odds Ratio (log scale)
20 40 60 80 100
Ventricular Extrasystoles
Funnel plot with pseudo 95% confidence limits
Standard Error of Log Odds Ratio
0 .5 1 1.5 2
Odds Ratio (log scale)
10 20 30 40
Supraventricular Extrasystoles
Funnel plot with pseudo 95% confidence limits
Standard Error of Log Odds Ratio
0 .5 1 1.5 2
Odds Ratio (log scale)
20 40 60
Low Voltage QRS
Funnel plot with pseudo 95% confidence limits
Standard Error of Log Odds Ratio
0 .5 1 1.5 2
Odds Ratio (log scale)
5 10 15 2025
